# The Hawaiian Rhodophyta Biodiversity Survey (2006-2010): a summary of principal findings

**DOI:** 10.1186/1471-2229-10-258

**Published:** 2010-11-22

**Authors:** Alison R Sherwood, Akira Kurihara, Kimberly Y Conklin, Thomas Sauvage, Gernot G Presting

**Affiliations:** 1 Botany Department, 3190 Maile Way, University of Hawaii, Honolulu, HI USA 96822; 2 Kobe University Research Center for Inland Seas, 1-1 Rokkodai, Kobe 657-8501 Japan; 3 Hawaii Institute of Marine Biology, P.O. Box 1346, Kaneohe, HI USA 96744; 4 Department of Molecular Biosciences and Bioengineering, 1955 East-West Rd, University of Hawaii, Honolulu, HI USA 96822

## Abstract

**Background:**

The Hawaiian red algal flora is diverse, isolated, and well studied from a morphological and anatomical perspective, making it an excellent candidate for assessment using a combination of traditional taxonomic and molecular approaches. Acquiring and making these biodiversity data freely available in a timely manner ensures that other researchers can incorporate these baseline findings into phylogeographic studies of Hawaiian red algae or red algae found in other locations.

**Results:**

A total of 1,946 accessions are represented in the collections from 305 different geographical locations in the Hawaiian archipelago. These accessions represent 24 orders, 49 families, 152 genera and 252 species/subspecific taxa of red algae. One order of red algae (the Rhodachlyales) was recognized in Hawaii for the first time and 196 new island distributional records were determined from the survey collections. One family and four genera are reported for the first time from Hawaii, and multiple species descriptions are in progress for newly discovered taxa. A total of 2,418 sequences were generated for Hawaiian red algae in the course of this study - 915 for the nuclear LSU marker, 864 for the plastidial UPA marker, and 639 for the mitochondrial COI marker. These baseline molecular data are presented as neighbor-joining trees to illustrate degrees of divergence within and among taxa. The LSU marker was typically most conserved, followed by UPA and COI. Phylogenetic analysis of a set of concatenated LSU, UPA and COI sequences recovered a tree that broadly resembled the current understanding of florideophyte red algal relationships, but bootstrap support was largely absent above the ordinal level. Phylogeographic trends are reported here for some common taxa within the Hawaiian Islands and include examples of those with, as well as without, intraspecific variation.

**Conclusions:**

The UPA and COI markers were determined to be the most useful of the three and are recommended for inclusion in future algal biodiversity surveys. Molecular data for the survey provide the most extensive assessment of Hawaiian red algal diversity and, in combination with the morphological/anatomical and distributional data collected as part of the project, provide a solid baseline data set for future studies of the flora. The data are freely available *via *the Hawaiian Algal Database (HADB), which was designed and constructed to accommodate the results of the project. We present the first DNA sequence reference collection for a tropical Pacific seaweed flora, whose value extends beyond Hawaii since many Hawaiian taxa are shared with other tropical areas.

## Background

The Rhodophyta, or red algae of Hawaii represent one of the best studied red algal floras worldwide from a morphological perspective [1], making it an ideal flora for molecular characterization. Endemism estimates for the marine (19.5%) and freshwater (20.0%) algal floras of the islands are based on species lists from compiled traditional taxonomic surveys, and the potential for unrecognized genetic diversity is high [1,2]. It is almost certain that these estimates will change as molecular techniques are applied on a more routine basis to the Hawaiian flora and in other areas of the tropical Pacific. Until this survey, only a small proportion of the published studies of Hawaiian Rhodophyta employed molecular approaches, although those few resulted in substantial taxonomic revision to the Hawaiian representatives of the genera *Akalaphycus*, *Galaxaura*, *Ganonema *and *Liagora *[3-5]. Additionally, a new red algal order (the Pihiellales) was described from Hawaiian material as a result of molecular phylogenetic analyses [6].

There has also been a bias toward the study of large, conspicuous members of the Hawaiian seaweed flora [1]. The red algae, however, display a vast array of morphologies and reproductive strategies [7], and the breadth of this diversity is often overlooked in general survey efforts. It is certain that more concentrated study of the less conspicuous turf and crustose morphologies, as well as the epiphytic and parasitic representatives, will yield unrecognized diversity [1,8]. Indeed, examination of a number of red algal taxa using molecular techniques has revealed cryptic diversity that was previously unrecognized based on morphology and anatomy, e.g. [9,10].

Red algal systematics and taxonomy are fraught with a number of issues, often making identification difficult. Since the end of the 19^th ^century, the classification of red algae has been largely based on female reproductive anatomy and post-fertilization events [7,11,12]. Later additions to the suite of taxonomic characters included the ultrastructure of pit connections between cells [13] and the use of DNA sequence data [14,15]. Current red algal systematics usually employs a blend of biological, morphological and evolutionary species concepts, depending on the subgroup under study. For example, although the ability to interbreed and produce fertile offspring is not usually explicitly tested in phycological studies, some researchers have shown the biological species concept to be applicable [16,17]. Morphology continues to play a key role in red algal systematics, although these data are now typically viewed in an evolutionary context, and the inclusion of molecular data has become commonplace in the last two decades.

The traditional system of relying exclusively on reproductive characters for taxonomic purposes has several disadvantages. These characters can be evaluated only for those taxa that undergo sexual reproduction, which may be encountered rarely in some taxa. Furthermore, a number of red algae from Hawaii are believed to be strictly asexual, including the genera *Stylonema*, *Chroodactylon*, *Erythrocladia*, *Erythrotrichia*, *Compsopogon *and *Hildenbrandia *[1,18,19]. For others, only asexual stages have been found in Hawaii, even though they are known to be sexually reproductive in other geographical regions (e.g. *Peyssonnelia *[1]). Some taxa are also highly seasonal in their timing of reproduction, meaning that collections must be made in a narrow window of the year to observe the necessary characters (e.g. *Acrosymphyton*, *Dudresnaya *and *Naccaria *[1]). Still other taxa rarely undergo sexual reproduction, with no apparent seasonal pattern (e.g. *Acrochaetium *[1]).

Heteromorphic taxa (those with morphologically distinct gametophyte and sporophyte life history phases) present another challenge for red algal systematics and, in Hawaii, include members of the Bangiales, Batrachospermales, Bonnemaisoniales, Gigartinales and some members of the Nemaliales [1,2,20]. Until recently, researchers have relied on culturing methods to link heteromorphic counterparts of the same taxon, and this approach has had profound impacts on the taxonomy of red algae [21,22]. A number of taxa in the Hawaiian Islands are known for which these two phases are spatially separated and morphologically distinguishable, and collecting often results in the recovery of only one of these phases. Often the gametophyte is the more conspicuous of the two generations, as an upright phase, while the tetrasporophyte is an overlooked filamentous or crustose stage [1,23]. In cases like these, DNA sequences associated with each morphological specimen can reveal molecular identity of morphologically distinct life history stages.

Biodiversity research is based on the ability to apply taxonomic names to collections. This is often not simple for the red algae, for reasons described above. DNA barcoding is a more recent development in biodiversity studies that has the potential to allow greater discrimination among cryptic species than traditional taxonomic methods. DNA barcodes are short, species-specific DNA sequences from one or more genetic loci, allowing for differentiation of individuals at the species level [24]. The purpose of a DNA barcode is to assign unknown individuals to species and to enhance the discovery of new species [25,26], both of which are fundamental aims of this project. One of the major components of the present study is the acquisition and analysis of short DNA marker sequences from the nuclear (partial 28 S rRNA gene, or LSU [27]), plastidial (partial 23 S rRNA gene, Universal Plastid Amplicon, or UPA [28,29]) and mitochondrial (partial cytochrome c oxidase I gene, or COI [9]) genomes of Hawaiian red algae to assess intraspecific variation and to provide a DNA sequence framework for the Hawaiian Rhodophyta.

The Hawaiian Rhodophyta Biodiversity Survey (2006-2010) was undertaken to document and archive the marine, freshwater and terrestrial red algae of the Hawaiian Islands with an unprecedented depth of analysis and coverage by vouchering morphological samples and total genomic DNA extracts and undertaking an assessment of DNA sequence diversity for as many samples as possible. Compilations of freely available taxonomic data sets such as this fulfil the mandate of the "Biodiversity Commons" [30]. Resulting archived collections are invaluable for studies into the phylogenetic relationships among taxa, species-level descriptions of the flora, preliminary population genetic analyses, and biogeographic studies within the Hawaiian Islands and among other tropical Pacific island systems.

The Hawaiian archipelago extends in a northwesterly direction for almost 2,400 km, includes a series of small islands, atolls and seamounts [31], and is well known as a center of endemism and biological uniqueness [32]. In 2006 the Northwestern Hawaiian Islands were declared the Papahanaumokuakea National Marine Monument, and this large portion of the archipelago is understudied for most algae. An imminent need exists to document the biodiversity of this unique island chain in the face of threats from ongoing alien and/or invasive species, algal blooms, climate change and corresponding sea-level rise [33,34]. Characterizing native biodiversity provides essential baseline data for future detection of algal introductions, and for monitoring changes in assemblages at particular sites. To this end, we undertook a series of collecting expeditions and analyses to document the Hawaiian red algal diversity, and here present a summary of our findings.

## Results

### Diversity of sampling locations

**Figure 1:**
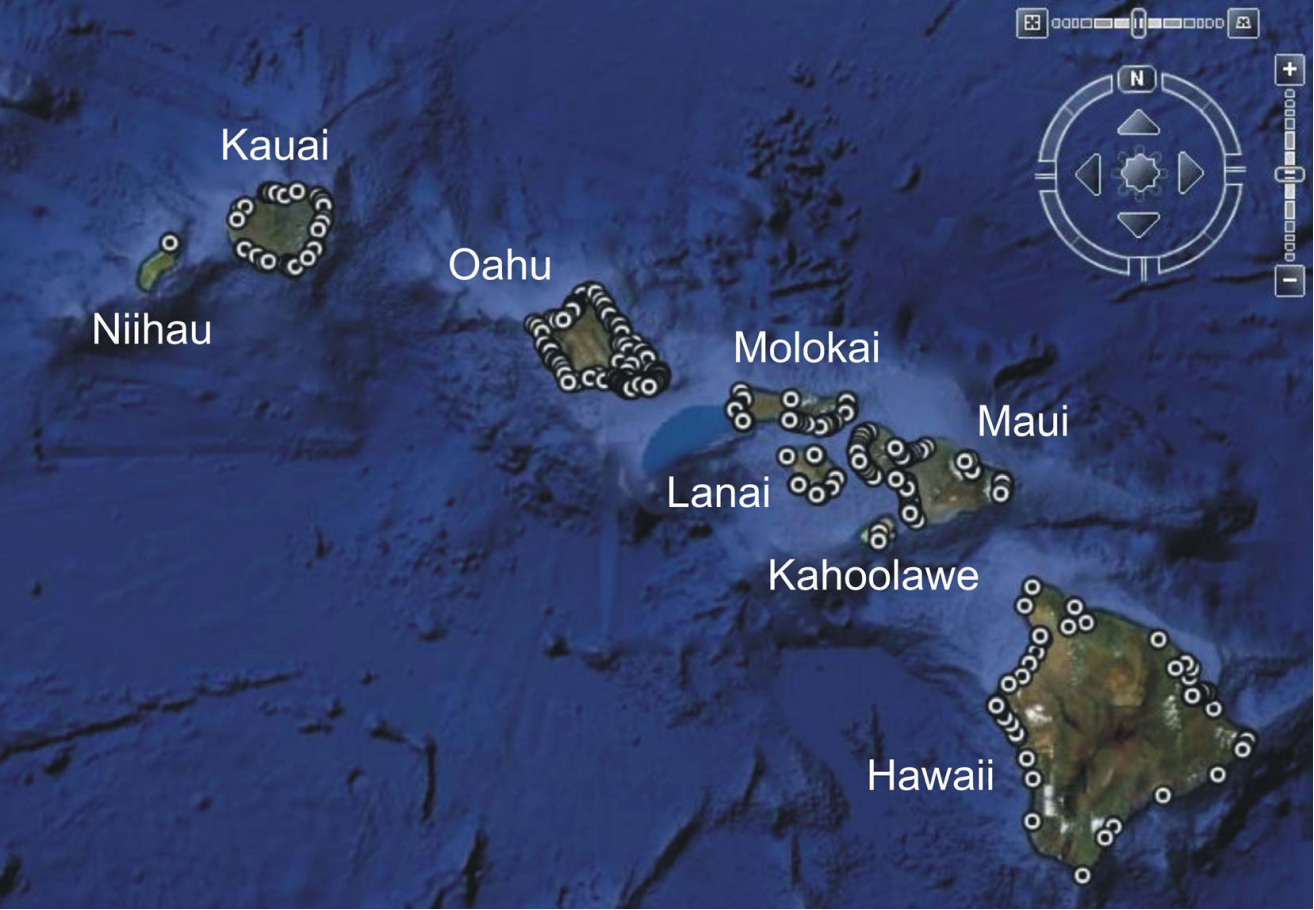
**Survey locations for the Hawaiian Rhodophyta Biodiversity Project**. Map of the Main Hawaiian Islands showing the distribution of 295 collecting sites from the Hawaiian Rhodophyta Biodiversity Project (12 sites from the Northwestern Hawaiian Islands were also sampled but are not illustrated on this map).

A total of 305 sites were sampled in the Hawaiian Rhodophyta Biodiversity Project; 293 from the Main Hawaiian Islands (MHI) and 12 from the Northwestern Hawaiian Islands (NWHI) (Figure 1, Additional file 1). The vast majority of the collecting sites were marine or brackish (290), while a much smaller number of freshwater (13) and terrestrial (2) locations were also included. Marine and brackish water sites were typically located in the intertidal and shallow subtidal areas, although some collections from SCUBA depth (up to ca. 60 m) were also included. Hawaii Undersea Research Laboratory (HURL) submersible collections (a total of 20 "sites", or container collections) were labelled according to dive and container data and provided representation from Hawaiian deep water habitats, but are not included in the above site totals since they are not accompanied by GPS data.

**Figure 2:**
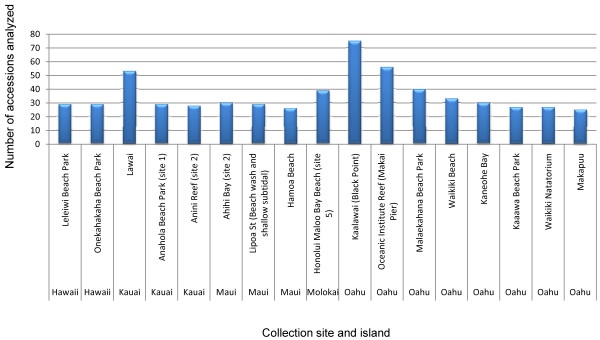
**Sites yielding large numbers of accessions**. Collecting sites with greater than (or equal to) 25 accessions are listed, by island, with their total number of accessions.

Seventeen sites yielded large numbers (≥25) of accessions (red algal individuals), including two from the island of Hawaii, three from Kauai, three from Maui, one from Molokai and eight from Oahu (Figure 2). Although the high number of accessions from these sites is partly based on accessibility (especially those from Oahu, which is where the authors' laboratories are based), they are still highly diverse locations that, in most cases, were surveyed multiple times because of their impressive numbers of taxa.

### Summary of Rhodophyta collections

The vast majority of accessions analyzed were from marine and brackish locations (1,928), while 16 were from freshwater habitats and two were from terrestrial locations. A total of 24 orders, 49 families, 152 genera and 252 species/subspecific taxa of red algae are represented in the collections (species counts do not include those identified only to genus as "sp."). Additional file 2 presents a checklist of taxa collected, identified and analyzed as part of the biodiversity survey, including new island and state records.

New records reported here do not include most of the coralline red algae (orders Corallinales and Sporolithales), which are being studied in detail by other researchers. In our new records, we included specimens from the Bernice P. Bishop Museum (BISH) that were not reported in Abbott's 1999 [1] assessment of distributional records, and also compared our distributional data to publications since this treatise and to those not covered therein. Included are those for freshwater red algae [35-39], descriptions of more recently recognized species of marine algae (e.g. *Dasya atropurpurea *[40]; the order Pihiellales [6]) and reports of previously unrecognized taxa (e.g. marine species of the genus *Hildenbrandia *[19]).

One new order of red algae (the Rhodachlyales) was recorded from our survey collections, although a number of additional new ordinal names are applied compared to those listed in Abbott [1] due to nomenclatural revision and inclusion of previously unreported taxa (i.e. the orders Acrosymphytales, Batrachospermales, Colaconematales, Compsopogonales, Halymeniales, Hildenbrandiales, Nemastomatales, Peyssonneliales, Pihiellales, Plocamiales, Rhodachlyales, Sporolithales, Stylonematales and Thoreales). The family Rhodachlyaceae is reported for the first time, as are the genera *Chroothece*, *Madagascaria*, *Pseudobangia *and *Rhodachlya*. Species designations for most of these newly reported genera are still being assessed, but it is likely that many of them represent undescribed species. Many new island distributional records (196 in total) were determined from the survey collections (Additional file 2).

Abbott [1] listed 343 species of marine red algae in the Hawaiian Islands, of which 218 were also identified from our biodiversity survey collections. The remaining 125 taxa, which included large numbers of species in the genera *Colaconema*, *Ceramium*/*Gayliella*, *Herposiphonia *and *Polysiphonia*/*Neosiphonia*, were not identified during our survey; however, these are primarily diminutive taxa that require a great deal of specialized morphological expertise for identification, and were not surveyed as readily as the more conspicuous macrophyte taxa. It is also possible that some of these taxa are not permanent members of the Hawaiian red algal flora since attempts to collect from reported localities have not yielded examples of these taxa.

### Summary of DNA sequence data

**Figure 3:**
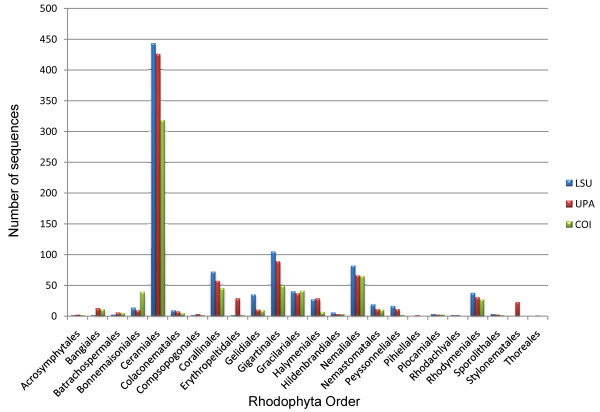
**Sequence counts by order**. The number of sequences for the LSU (blue), UPA (red) and COI (green) markers for each red algal order studied as part of the Hawaiian Rhodophyta Biodiversity Survey.

**Figure 4:**
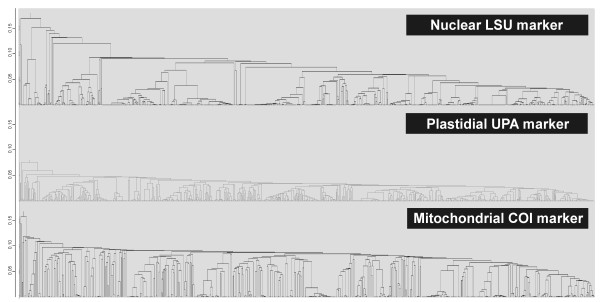
**Comparison of sequence marker data for biodiversity assessment**. UPGMA phenograms for Hawaiian Rhodophyta based on nuclear (top panel), plastidial (center panel) and mitochondrial sequences (bottom panel). Differences in the resolution of the three markers are evident. Scale on y-axis = evolutionary distances based on the Maximum Composite Likelihood method.

A total of 2,418 sequences were generated for Hawaiian red algae through this study - 915 for the nuclear LSU marker [GenBank: FJ554841-FJ554852, GU223830-GU223850, HQ421677-HQ422584], 864 for the plastidial UPA marker [GenBank: EU628656, FJ554863-FJ554875, HM582901-HM582908, EF426600-EF426661, HQ420906-HQ421676], and 639 for the mitochondrial COI marker [GenBank: EU636722, FJ554853-FJ554858, HM582889-HM582900, EU146155-EU146190, GU223879-GU223896, HQ422585-HQ423135] (Figure 3, Additional file 3). The LSU and UPA markers amplified and sequenced across the red algae with the greatest ease, as illustrated by the total number of sequences obtained. However, the resolution of the three markers was quite different, with the COI marker being the least conserved (as evidenced by the abundance of high divergences, or long branches), the LSU marker being relatively divergent at higher taxonomic levels but conserved for closely related species, and the UPA marker being approximately twice as conserved as COI, but with a similar topological pattern (Figure 4).

### DNA sequence diversity of Hawaiian Rhodophyta

Sequence data for each of the three markers sequenced (UPA, COI and LSU) were employed to investigate the molecular diversity of Hawaiian red algae. Sequence diversity for each sampled taxon for the three markers is presented as a series of neighbor-joining (NJ) trees in Figures 5, 6, 7, 8, 9, 10, 11, 12, 13, 14, 15, 16, 17, 18, 19, 20, 21, 22, 23, 24, 25, 26, 27, 28, 29, 30, 31, 32, 33, 34, 35, 36, 37, 38 and 39 (except for the LSU marker for the Bangiales, Compsopogonales, Erythropeltidales and Stylonematales, since very few sequences were obtained). Taxa are grouped by order (or groups of related orders), or by family (or parts of families, or groups of families) in the case of the Ceramiales (due to tree size). Since only short molecular markers were used (COI, which is employed as the DNA barcode for red algae [9,41] and UPA and LSU, which are also <700 bp in length), we did not attempt to reconstruct phylogenetic relationships among our collections for the individual marker data sets, nor assess branch support, but instead focused on patterns of diversity for species and groups of closely related species (i.e. DNA barcoding analyses [9,41,42]). Thus, higher order relationships in the NJ sequence diversity trees should not be evaluated.

**Figure 5:**
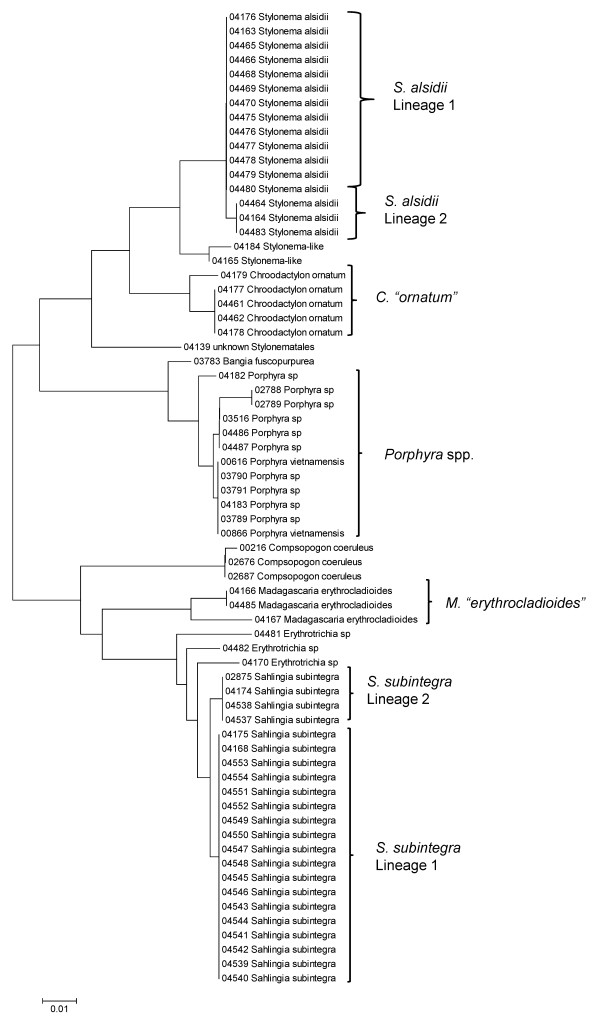
**Neighbor-joining tree of UPA sequences of the Bangiales, Compsopogonales, Erythropeltidales and Stylonematales**. Sequence diversity based on the UPA plastid marker for surveyed Rhodophyta specimens belonging to the orders Bangiales, Compsopogonales, Erythropeltidales and Stylonematales. Neighbor-joining tree is based on the Maximum Composite Likelihood nucleotide model in MEGA 4.0. Scale bar = substitutions per site.

**Figure 6:**
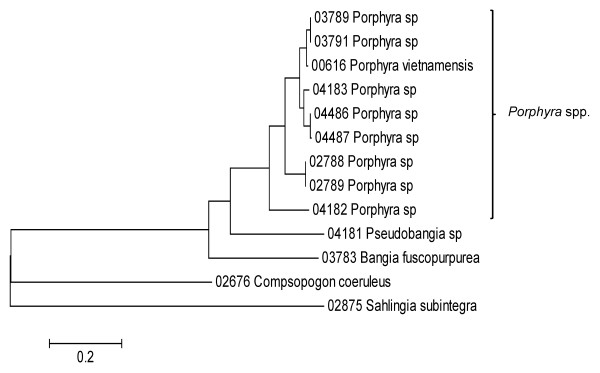
**Neighbor-joining tree of COI sequences of the Bangiales, Compsopogonales and Erythropeltidales**. Sequence diversity based on the COI mitochondrial marker for surveyed Rhodophyta specimens belonging to the orders Bangiales, Compsopogonales and Erythropeltidales. Neighbor-joining tree is based on the Maximum Composite Likelihood nucleotide model in MEGA 4.0. Scale bar = substitutions per site.

**Figure 7:**
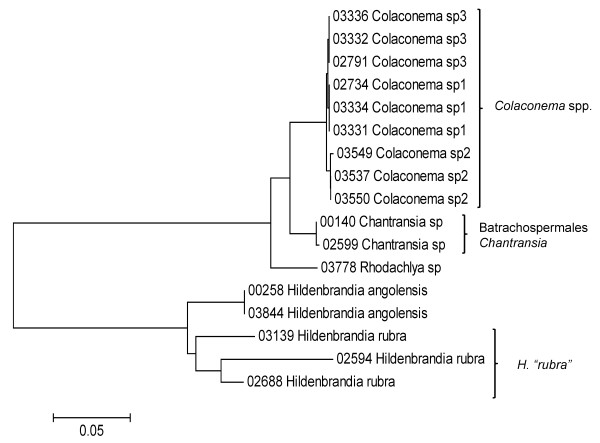
**Neighbor-joining tree of LSU sequences of the Batrachospermales, Colaconematales, Hildenbrandiales, Rhodachlyales and Thoreales**. Sequence diversity based on the LSU nuclear marker for surveyed Rhodophyta specimens belonging to the orders Batrachospermales, Colaconematales, Hildenbrandiales, Rhodachlyales and Thoreales. Neighbor-joining tree is based on the Maximum Composite Likelihood nucleotide model in MEGA 4.0. Scale bar = substitutions per site.

**Figure 8:**
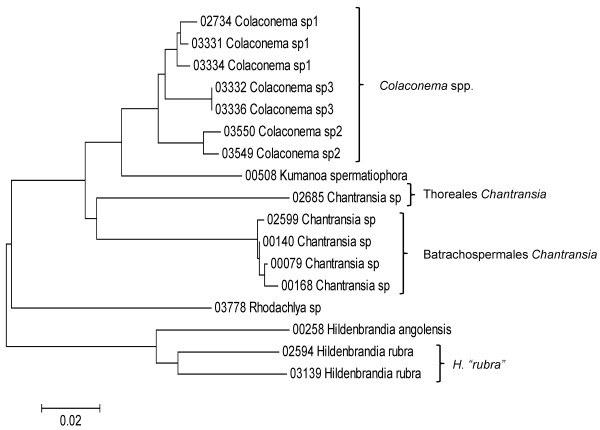
**Neighbor-joining tree of UPA sequences of the Batrachospermales, Colaconematales, Hildenbrandiales and Thoreales**. Sequence diversity based on the UPA plastid marker for surveyed Rhodophyta specimens belonging to the orders Batrachospermales, Colaconematales, Hildenbrandiales and Thoreales. Neighbor-joining tree is based on the Maximum Composite Likelihood nucleotide model in MEGA 4.0. Scale bar = substitutions per site.

**Figure 9:**
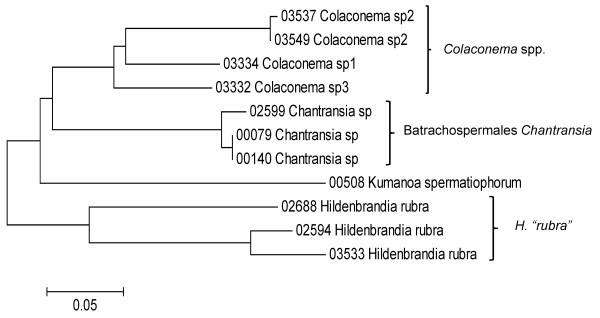
**Neighbor-joining tree of COI sequences of the Batrachospermales, Colaconematales, Hildenbrandiales and Thoreales**. Sequence diversity based on the COI mitochondrial marker for surveyed Rhodophyta specimens belonging to the orders Batrachospermales, Colaconematales, Hildenbrandiales and Thoreales. Neighbor-joining tree is based on the Maximum Composite Likelihood nucleotide model in MEGA 4.0. Scale bar = substitutions per site.

**Figure 10:**
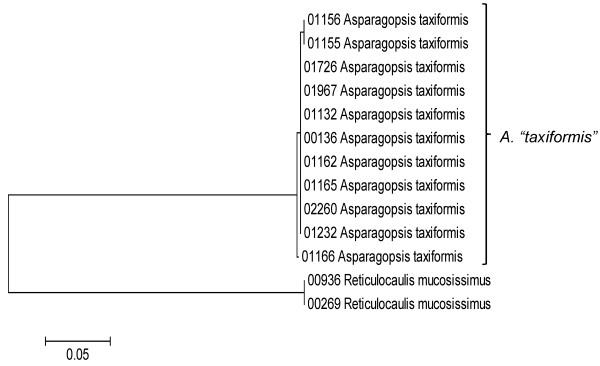
**Neighbor-joining tree of LSU sequences of the Bonnemaisoniales**. Sequence diversity based on the LSU nuclear marker for surveyed Rhodophyta specimens belonging to the order Bonnemaisoniales. Neighbor-joining tree is based on the Maximum Composite Likelihood nucleotide model in MEGA 4.0. Scale bar = substitutions per site.

**Figure 11:**
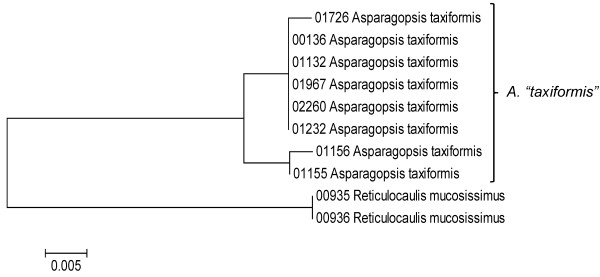
**Neighbor-joining tree of UPA sequences of the Bonnemaisoniales**. Sequence diversity based on the UPA plastid marker for surveyed Rhodophyta specimens belonging to the order Bonnemaisoniales. Neighbor-joining tree is based on the Maximum Composite Likelihood nucleotide model in MEGA 4.0. Scale bar = substitutions per site.

**Figure 12:**
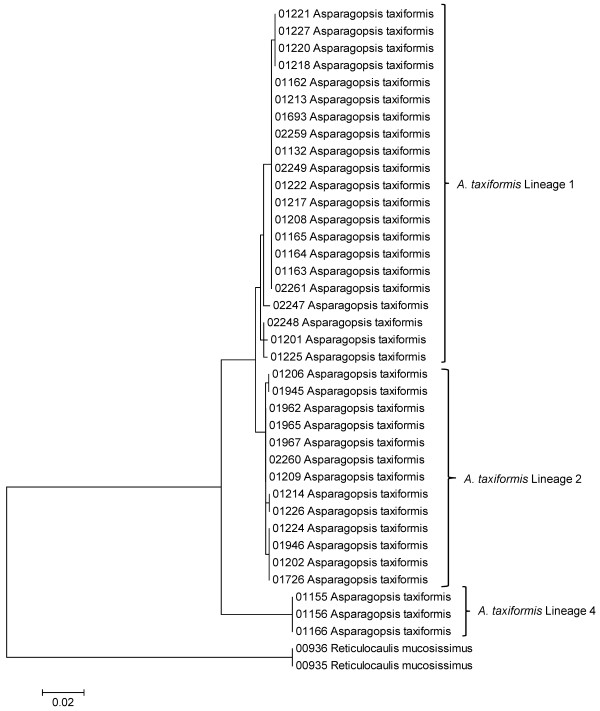
**Neighbor-joining tree of COI sequences of the Bonnemaisoniales**. Sequence diversity based on the COI mitochondrial marker for surveyed Rhodophyta specimens belonging to the order Bonnemaisoniales. Mitochondrial lineages 1-3 of *Asparagopsis taxiformis *are labelled following the convention of [43,45]. Neighbor-joining tree is based on the Maximum Composite Likelihood nucleotide model in MEGA 4.0. Scale bar = substitutions per site.

**Figure 13:**
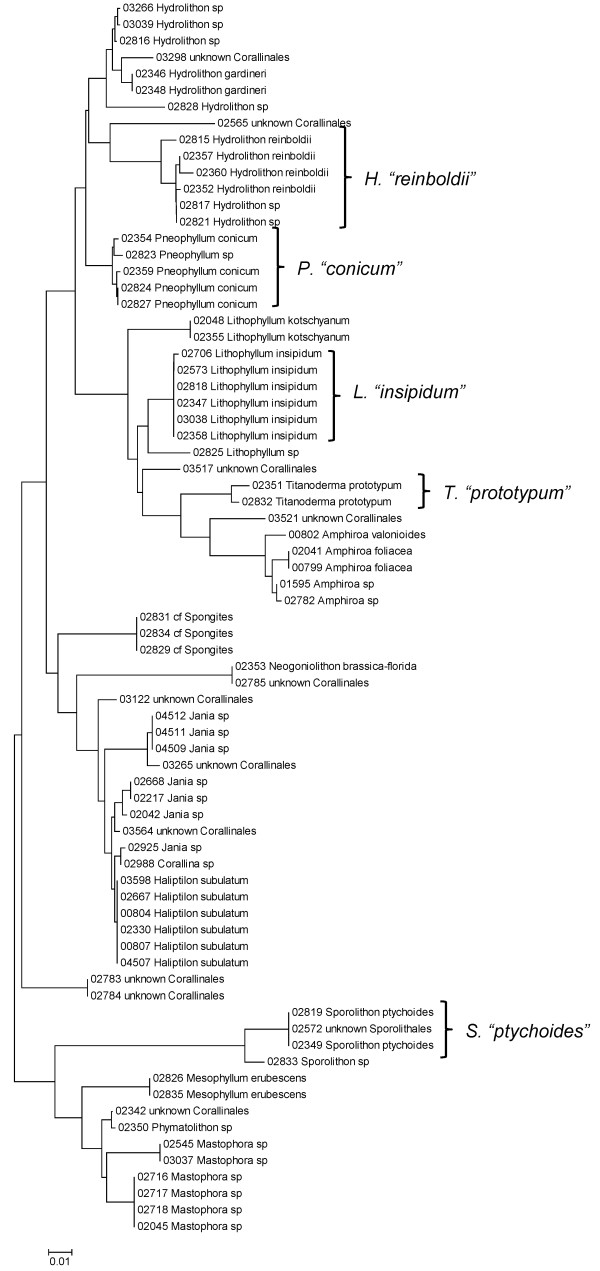
**Neighbor-joining tree of LSU sequences of the Corallinales and Sporolithales**. Sequence diversity based on the LSU nuclear marker for surveyed Rhodophyta specimens belonging to the orders Corallinales and Sporolithales. Neighbor-joining tree is based on the Maximum Composite Likelihood nucleotide model in MEGA 4.0. Scale bar = substitutions per site.

**Figure 14:**
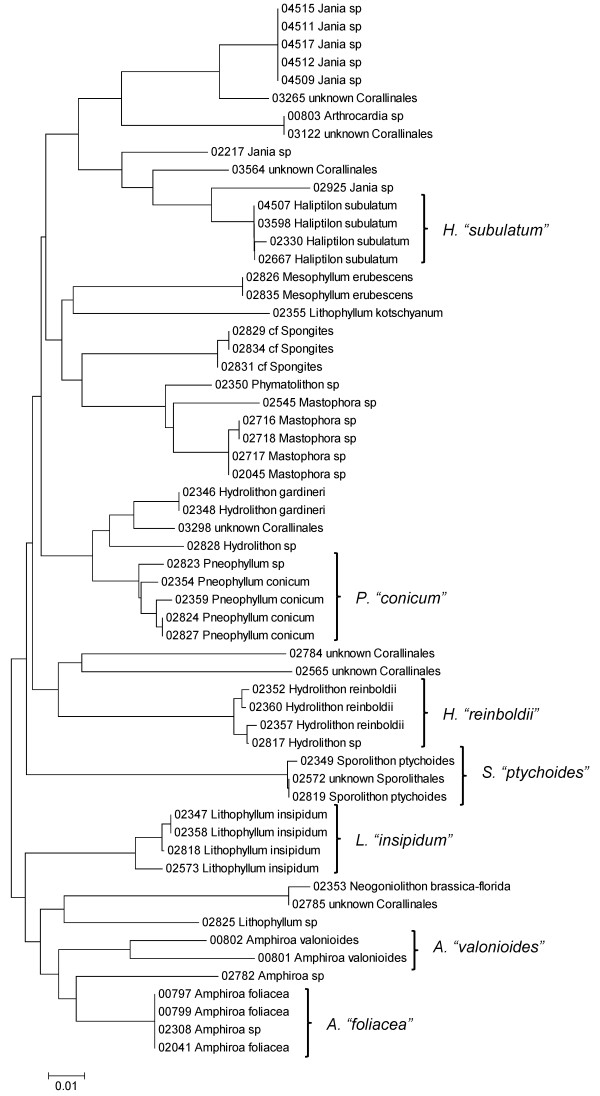
**Neighbor-joining tree of UPA sequences of the Corallinales and Sporolithales**. Sequence diversity based on the UPA plastid marker for surveyed Rhodophyta specimens belonging to the orders Corallinales and Sporolithales. Neighbor-joining tree is based on the Maximum Composite Likelihood nucleotide model in MEGA 4.0. Scale bar = substitutions per site.

**Figure 15:**
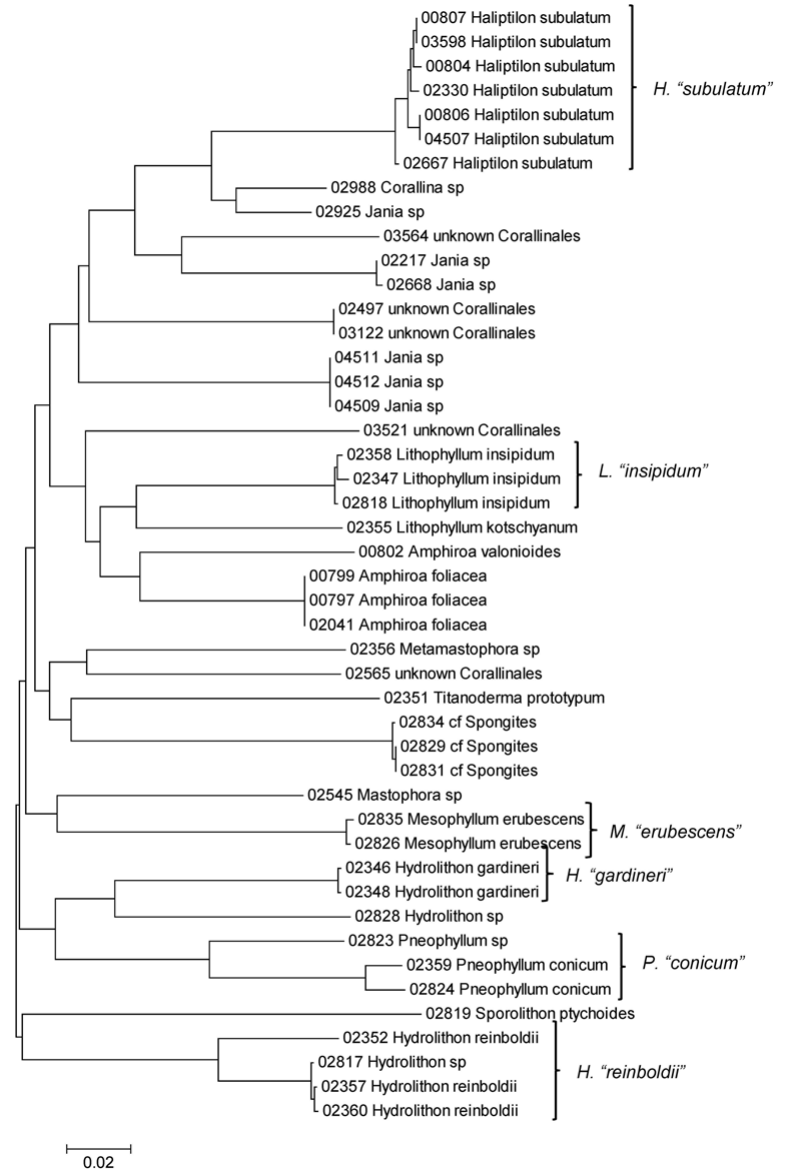
**Neighbor-joining tree of COI sequences of the Corallinales and Sporolithales**. Sequence diversity based on the COI mitochondrial marker for surveyed Rhodophyta specimens belonging to the orders Corallinales and Sporolithales. Neighbor-joining tree is based on the Maximum Composite Likelihood nucleotide model in MEGA 4.0. Scale bar = substitutions per site.

**Figure 16:**
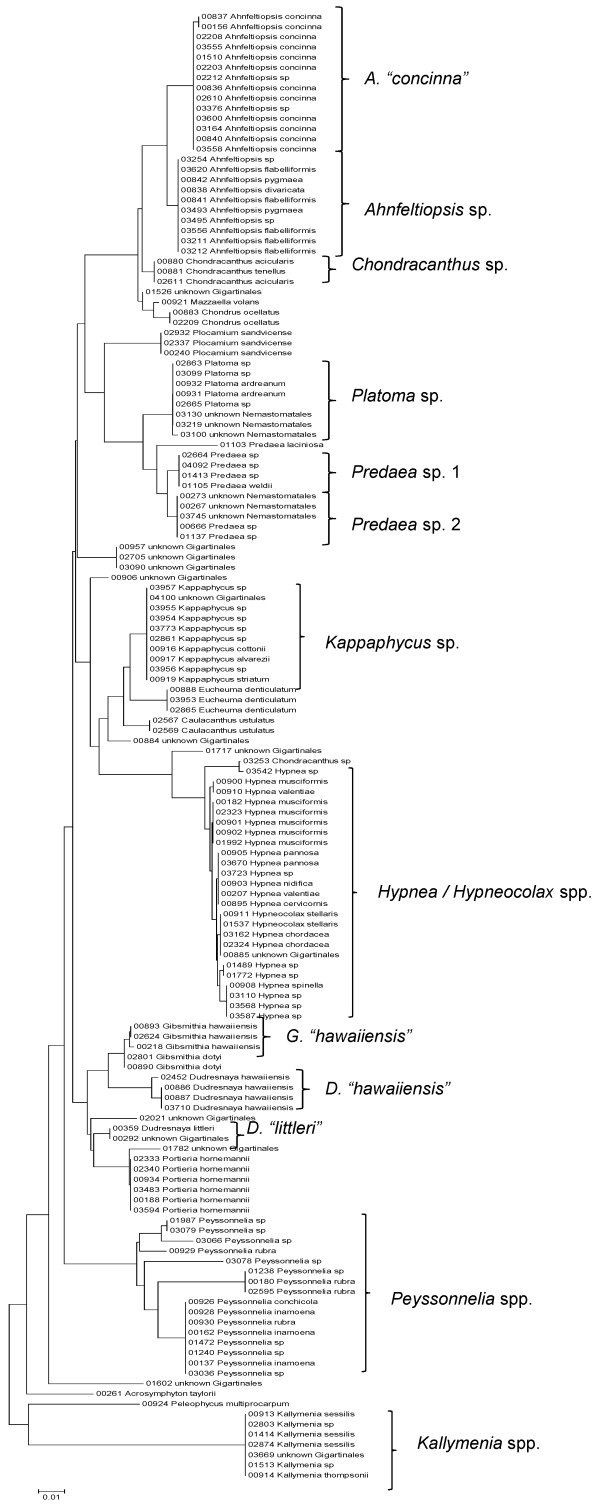
**Neighbor-joining tree of LSU sequences of the Acrosymphytales, Gigartinales, Nemastomatales, Peyssonneliales and Plocamiales**. Sequence diversity based on the LSU nuclear marker for surveyed Rhodophyta specimens belonging to the orders Acrosymphytales, Gigartinales, Nemastomatales, Peyssonneliales and Plocamiales. Neighbor-joining tree is based on the Maximum Composite Likelihood nucleotide model in MEGA 4.0. Scale bar = substitutions per site.

**Figure 17:**
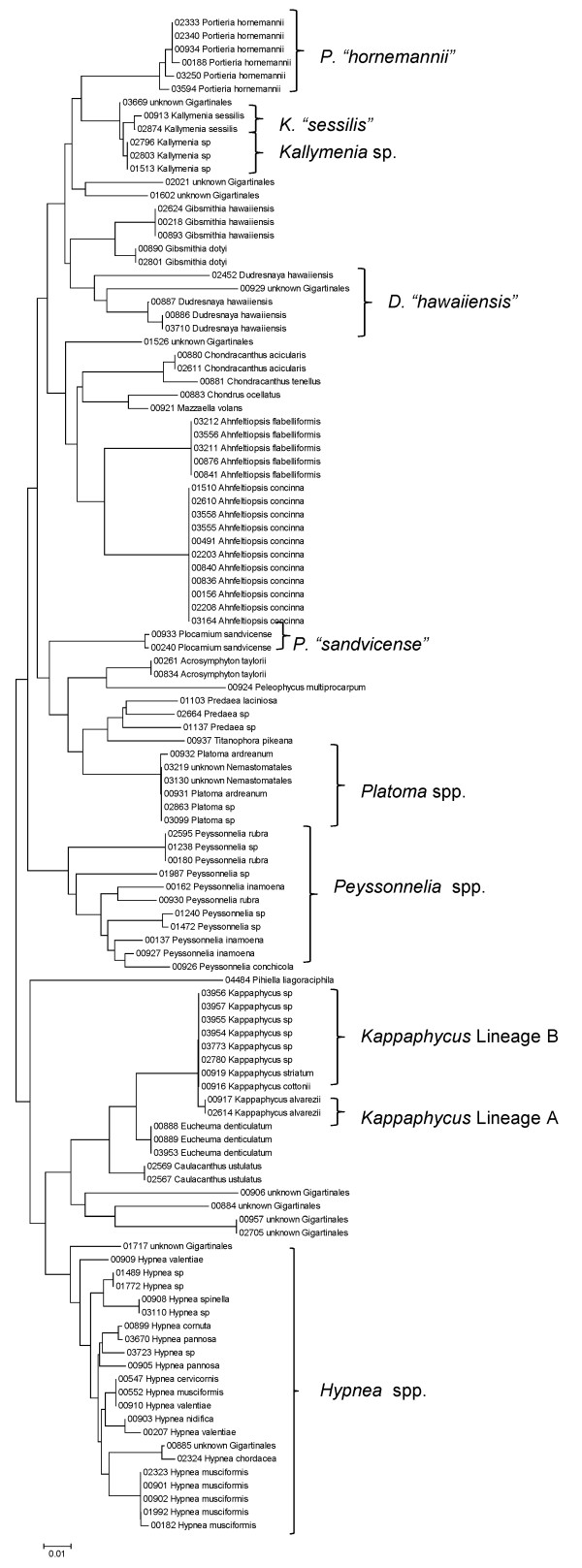
**Neighbor-joining tree of UPA sequences of the Acrosymphytales, Gigartinales, Nemastomatales, Peyssonneliales, Pihiellales and Plocamiales**. Sequence diversity based on the UPA plastid marker for surveyed Rhodophyta specimens belonging to the orders Acrosymphytales, Gigartinales, Nemastomatales, Peyssonneliales, Pihiellales and Plocamiales. UPA lineages A and B of *Kappaphycus *are labelled following the convention of [27]. Neighbor-joining tree is based on the Maximum Composite Likelihood nucleotide model in MEGA 4.0. Scale bar = substitutions per site.

**Figure 18:**
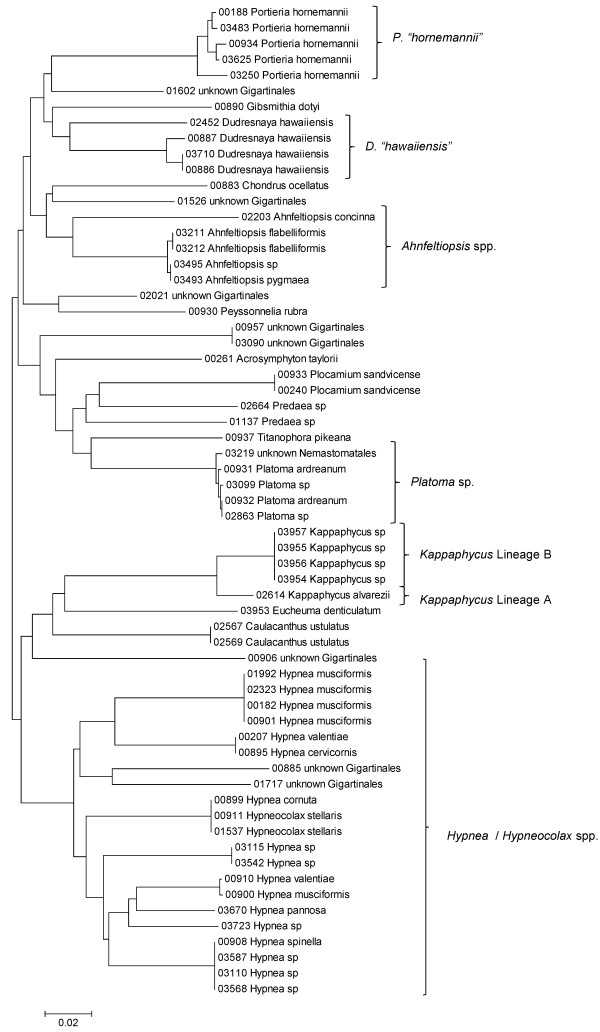
**Neighbor-joining tree of COI sequences of the Acrosymphytales, Gigartinales, Nemastomatales, Peyssonneliales and Plocamiales**. Sequence diversity based on the COI mitochondrial marker for surveyed Rhodophyta specimens belonging to the orders Acrosymphytales, Gigartinales, Nemastomatales, Peyssonneliales and Plocamiales. COI lineages A and B of *Kappaphycus *are labelled following the convention of [27]. Neighbor-joining tree is based on the Maximum Composite Likelihood nucleotide model in MEGA 4.0. Scale bar = substitutions per site.

**Figure 19:**
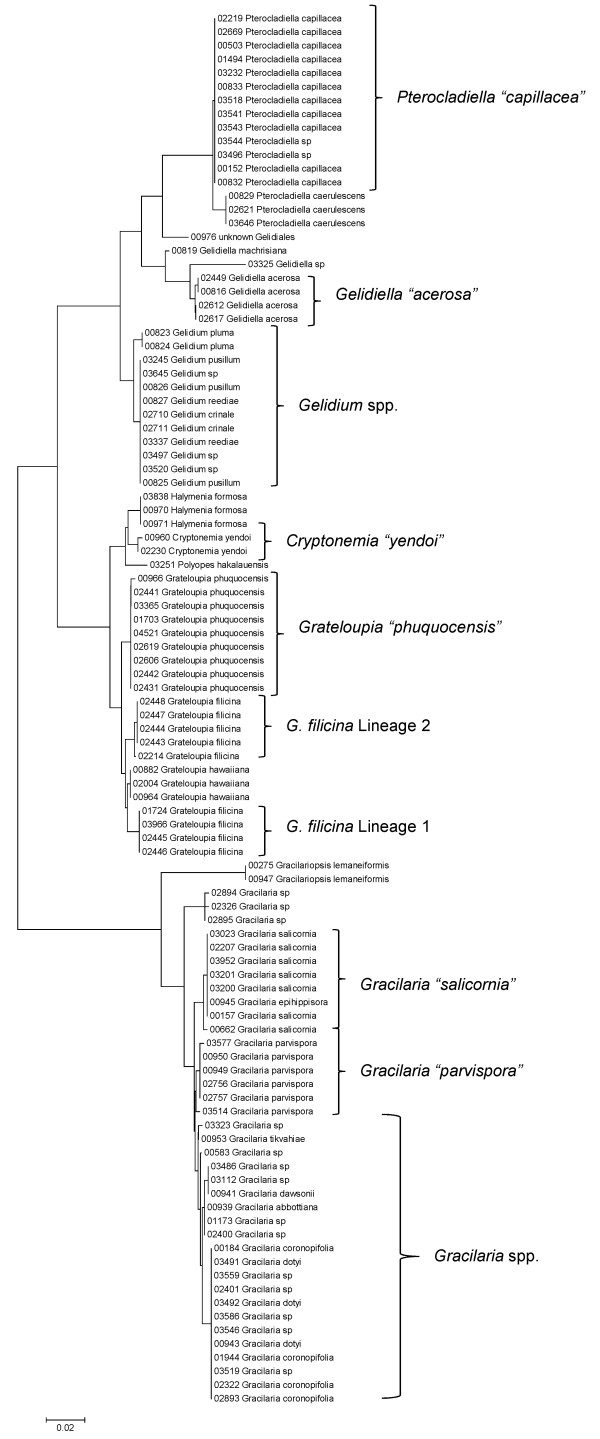
**Neighbor-joining tree of LSU sequences of the Gelidiales, Gracilariales and Halymeniales**. Sequence diversity based on the LSU nuclear marker for surveyed Rhodophyta specimens belonging to the orders Gelidiales, Gracilariales and Halymeniales. Neighbor-joining tree is based on the Maximum Composite Likelihood nucleotide model in MEGA 4.0. Scale bar = substitutions per site.

**Figure 20:**
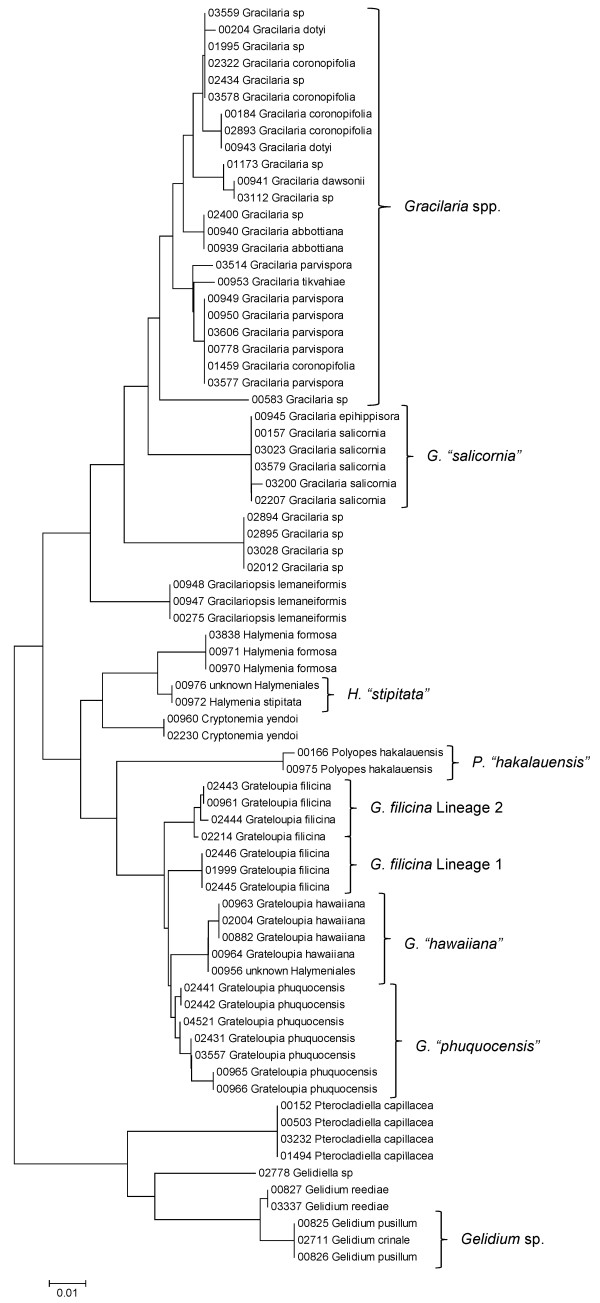
**Neighbor-joining tree of UPA sequences of the Gelidiales, Gracilariales and Halymeniales**. Sequence diversity based on the UPA plastid marker for surveyed Rhodophyta specimens belonging to the orders Gelidiales, Gracilariales and Halymeniales. Neighbor-joining tree is based on the Maximum Composite Likelihood nucleotide model in MEGA 4.0. Scale bar = substitutions per site.

**Figure 21:**
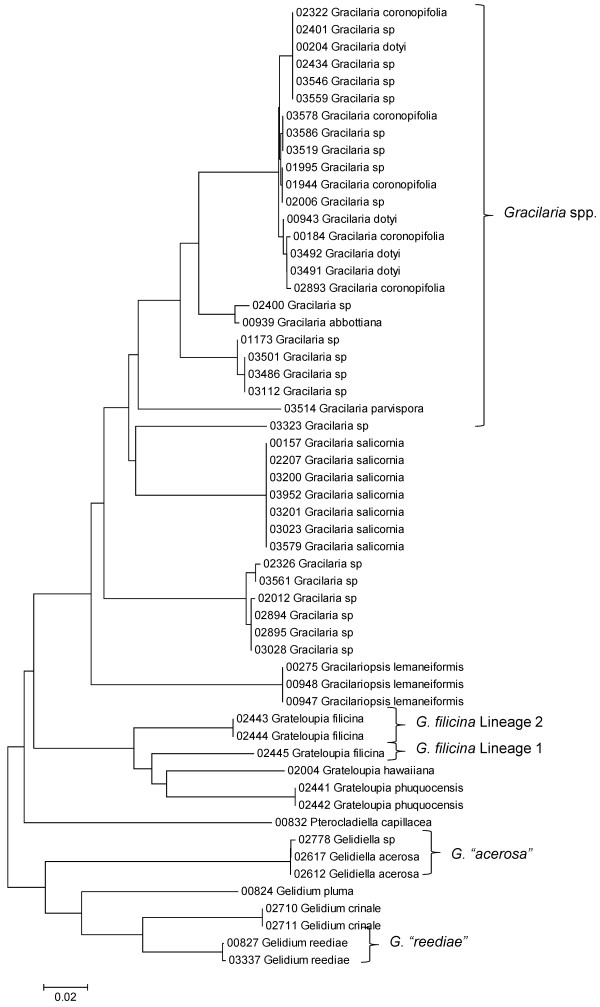
**Neighbor-joining tree of COI sequences of the Gelidiales, Gracilariales and Halymeniales**. Sequence diversity based on the COI mitochondrial marker for surveyed Rhodophyta specimens belonging to the orders Gelidiales, Gracilariales and Halymeniales. Neighbor-joining tree is based on the Maximum Composite Likelihood nucleotide model in MEGA 4.0. Scale bar = substitutions per site.

**Figure 22:**
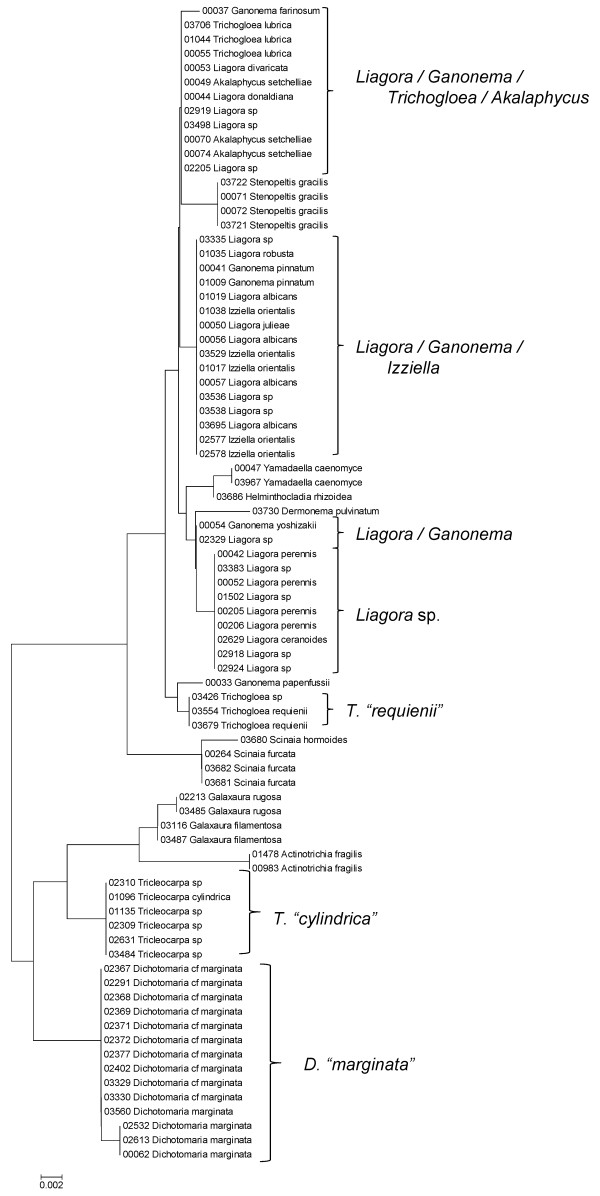
**Neighbor-joining tree of LSU sequences of the Nemaliales**. Sequence diversity based on the LSU nuclear marker for surveyed Rhodophyta specimens belonging to the order Nemaliales. Neighbor-joining tree is based on the Maximum Composite Likelihood nucleotide model in MEGA 4.0. Scale bar = substitutions per site.

**Figure 23:**
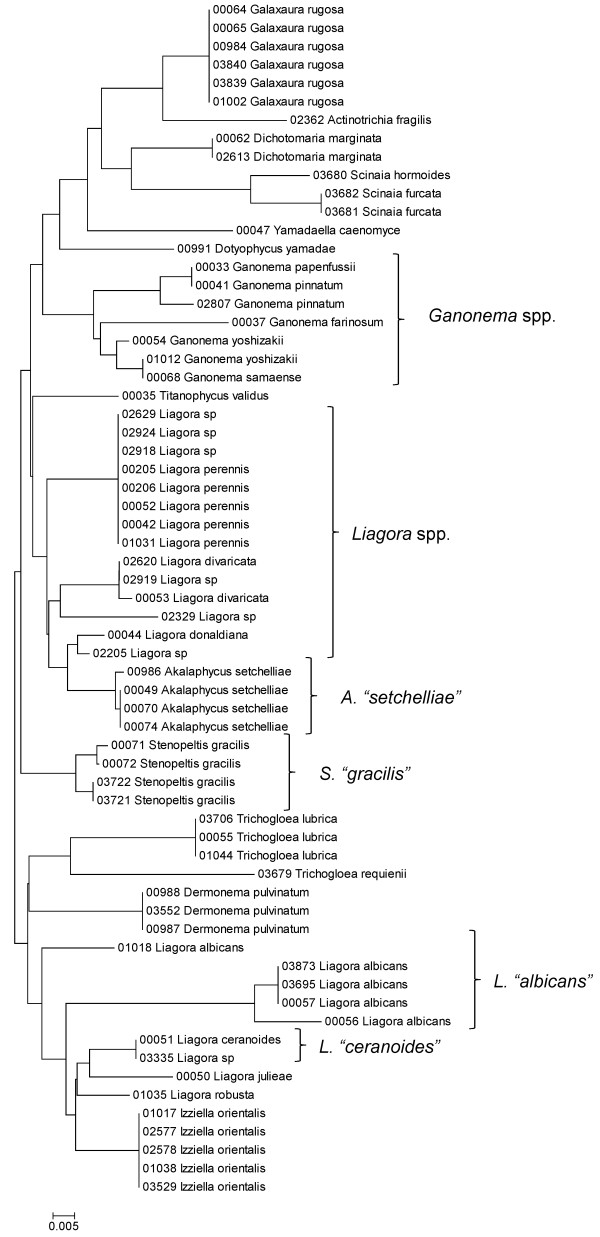
**Neighbor-joining tree of UPA sequences of the Nemaliales**. Sequence diversity based on the UPA plastid marker for surveyed Rhodophyta specimens belonging to the order Nemaliales. Neighbor-joining tree is based on the Maximum Composite Likelihood nucleotide model in MEGA 4.0. Scale bar = substitutions per site.

**Figure 24:**
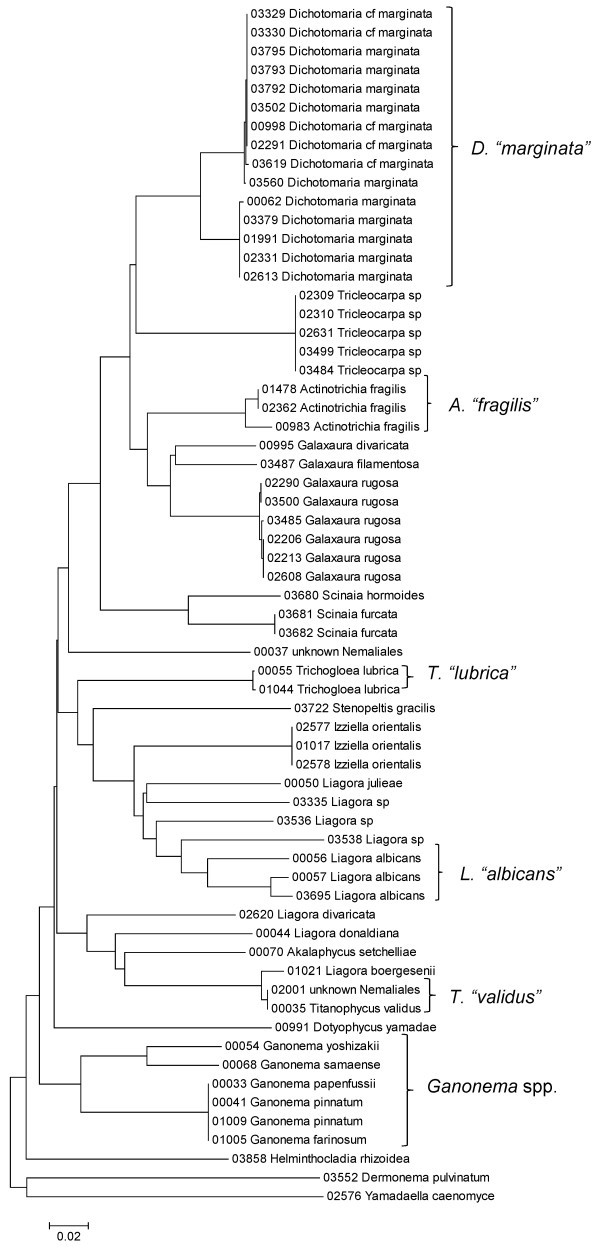
**Neighbor-joining tree of COI sequences of the Nemaliales**. Sequence diversity based on the COI mitochondrial marker for surveyed Rhodophyta specimens belonging to the order Nemaliales. Neighbor-joining tree is based on the Maximum Composite Likelihood nucleotide model in MEGA 4.0. Scale bar = substitutions per site.

**Figure 25:**
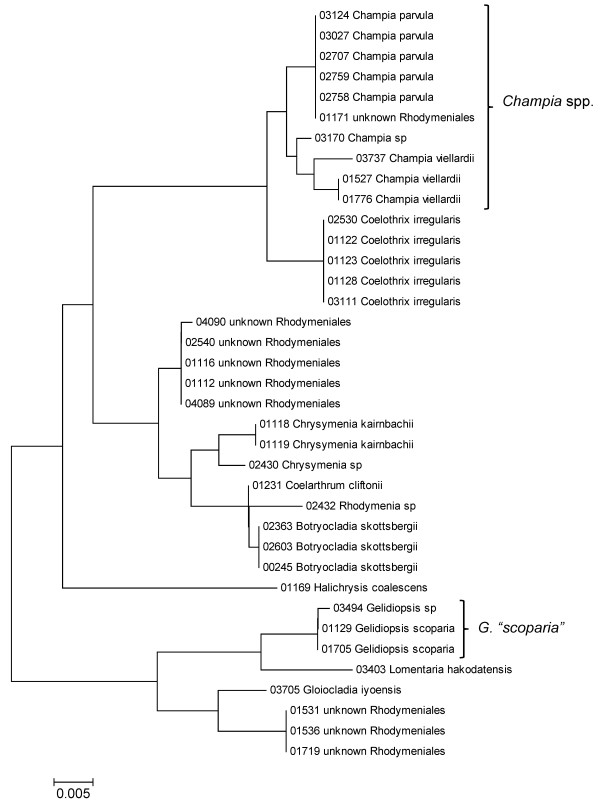
**Neighbor-joining tree of LSU sequences of the Rhodymeniales**. Sequence diversity based on the LSU nuclear marker for surveyed Rhodophyta specimens belonging to the order Rhodymeniales. Neighbor-joining tree is based on the Maximum Composite Likelihood nucleotide model in MEGA 4.0. Scale bar = substitutions per site.

**Figure 26:**
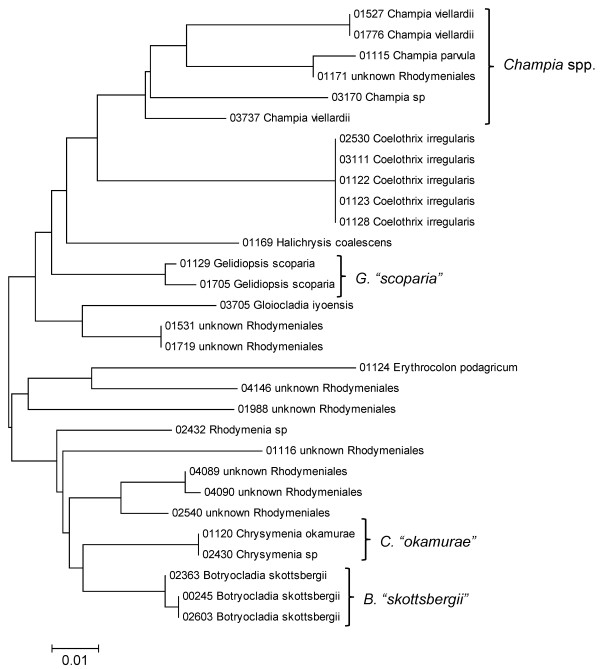
**Neighbor-joining tree of UPA sequences of the Rhodymeniales**. Sequence diversity based on the UPA plastid marker for surveyed Rhodophyta specimens belonging to the order Rhodymeniales. Neighbor-joining tree is based on the Maximum Composite Likelihood nucleotide model in MEGA 4.0. Scale bar = substitutions per site.

**Figure 27:**
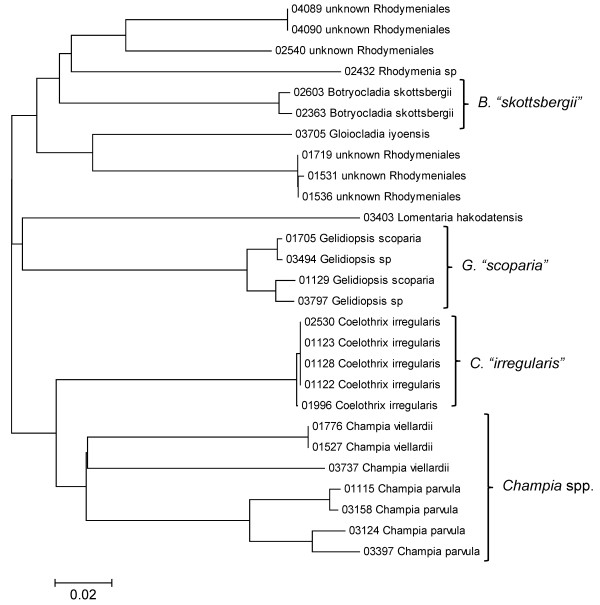
**Neighbor-joining tree of COI sequences of the Rhodymeniales**. Sequence diversity based on the COI mitochondrial marker for surveyed Rhodophyta specimens belonging to the order Rhodymeniales. Neighbor-joining tree is based on the Maximum Composite Likelihood nucleotide model in MEGA 4.0. Scale bar = substitutions per site.

**Figure 28:**
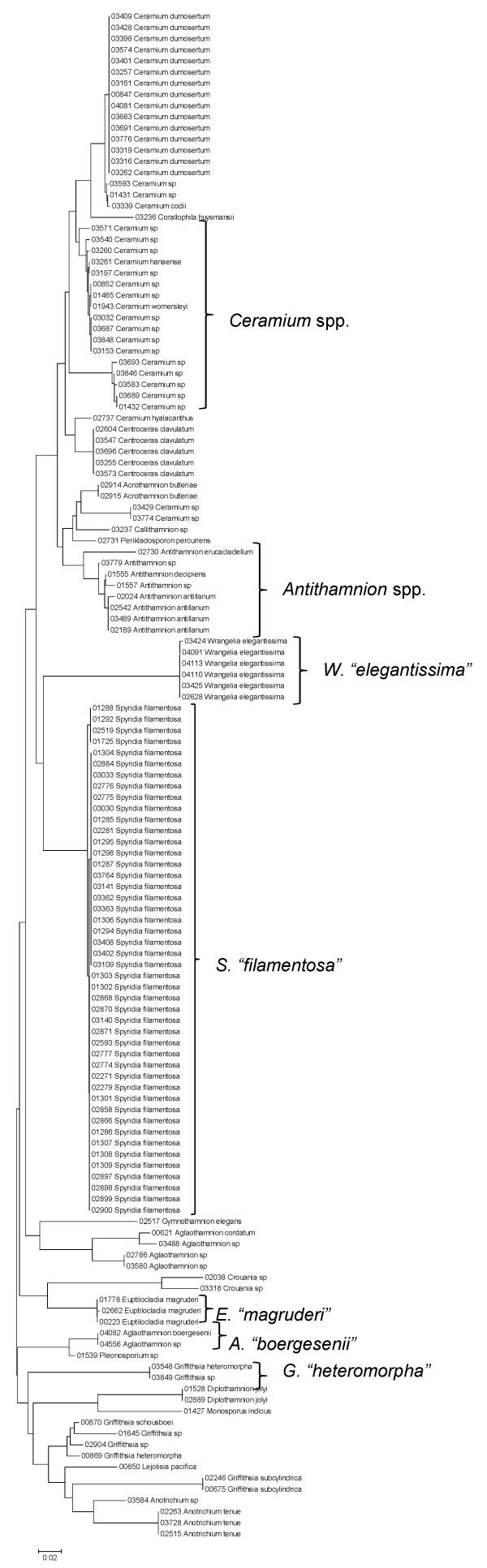
**Neighbor-joining tree of LSU sequences of the families Callithamniaceae, Ceramiaceae, Spyridiaceae and Wrangeliaceae of the order Ceramiales**. Sequence diversity based on the LSU nuclear marker for surveyed Rhodophyta specimens belonging to the families Callithamniaceae, Ceramiaceae, Spyridiaceae and Wrangeliaceae of the order Ceramiales. Neighbor-joining tree is based on the Maximum Composite Likelihood nucleotide model in MEGA 4.0. Scale bar = substitutions per site.

**Figure 29:**
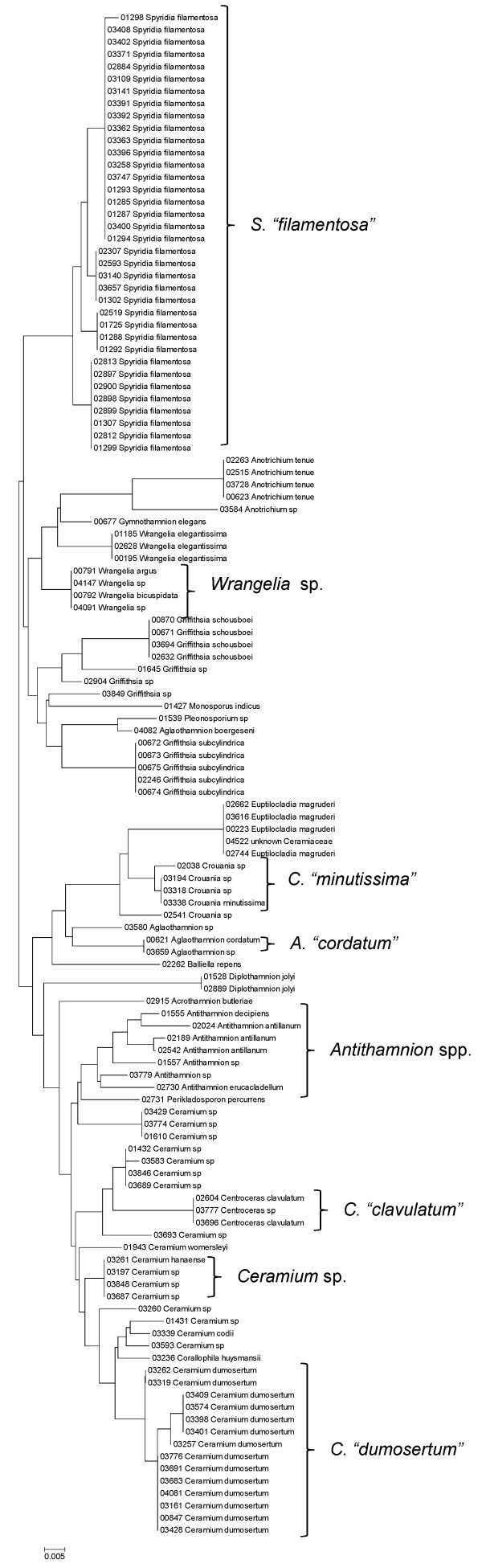
**Neighbor-joining tree of UPA sequences of the families Callithamniaceae, Ceramiaceae, Spyridiaceae and Wrangeliaceae of the order Ceramiales**. Sequence diversity based on the UPA plastid marker for surveyed Rhodophyta specimens belonging to the families Callithamniaceae, Ceramiaceae, Spyridiaceae and Wrangeliaceae of the order Ceramiales. Neighbor-joining tree is based on the Maximum Composite Likelihood nucleotide model in MEGA 4.0. Scale bar = substitutions per site.

**Figure 30:**
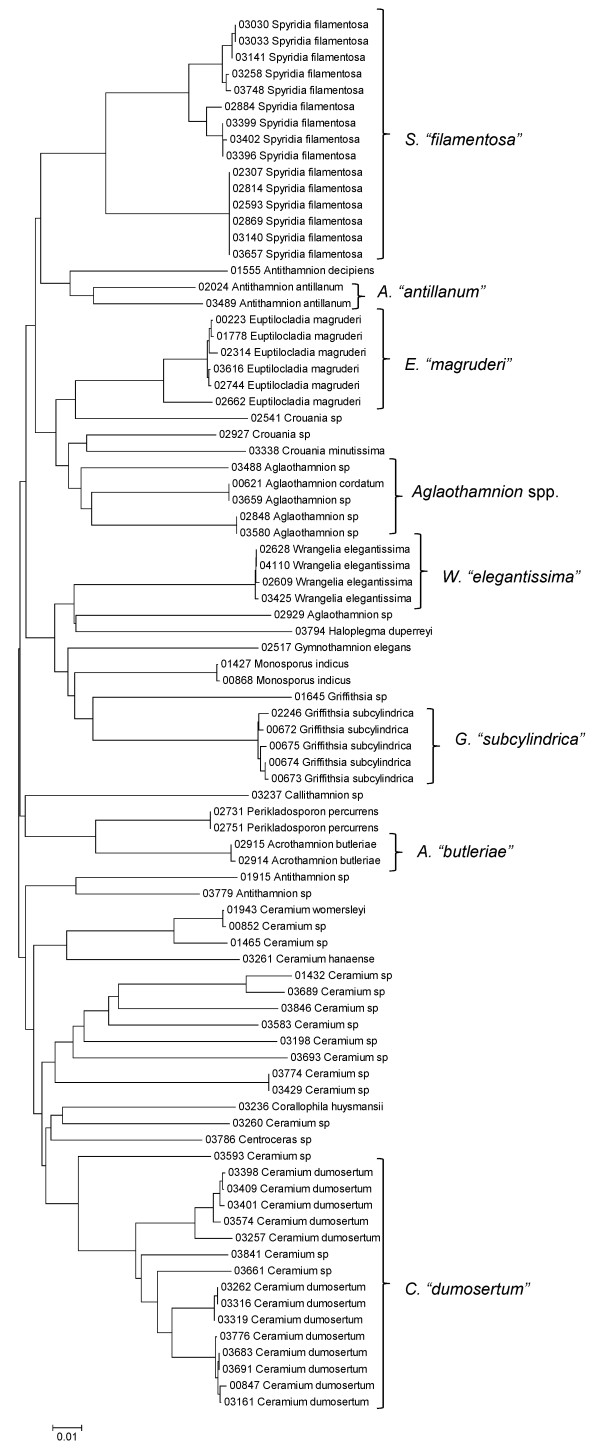
**Neighbor-joining tree of COI sequences of the families Callithamniaceae, Ceramiaceae, Spyridiaceae and Wrangeliaceae of the order Ceramiales**. Sequence diversity based on the COI mitochondrial marker for surveyed Rhodophyta specimens belonging to the families Callithamniaceae, Ceramiaceae, Spyridiaceae and Wrangeliaceae of the order Ceramiales. Neighbor-joining tree is based on the Maximum Composite Likelihood nucleotide model in MEGA 4.0. Scale bar = substitutions per site.

**Figure 31:**
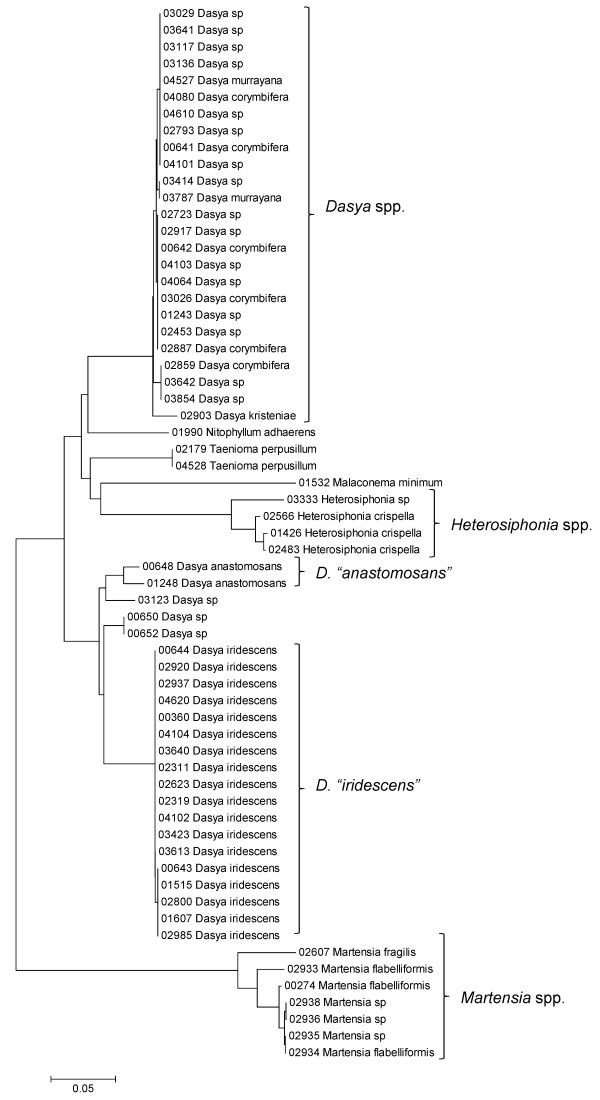
**Neighbor-joining tree of LSU sequences of the families Dasyaceae, Delesseriaceae and Sarcomeniaceae of the order Ceramiales**. Sequence diversity based on the LSU nuclear marker for surveyed Rhodophyta specimens belonging to the families Dasyaceae, Delesseriaceae and Sarcomeniaceae of the order Ceramiales. Neighbor-joining tree is based on the Maximum Composite Likelihood nucleotide model in MEGA 4.0. Scale bar = substitutions per site.

**Figure 32:**
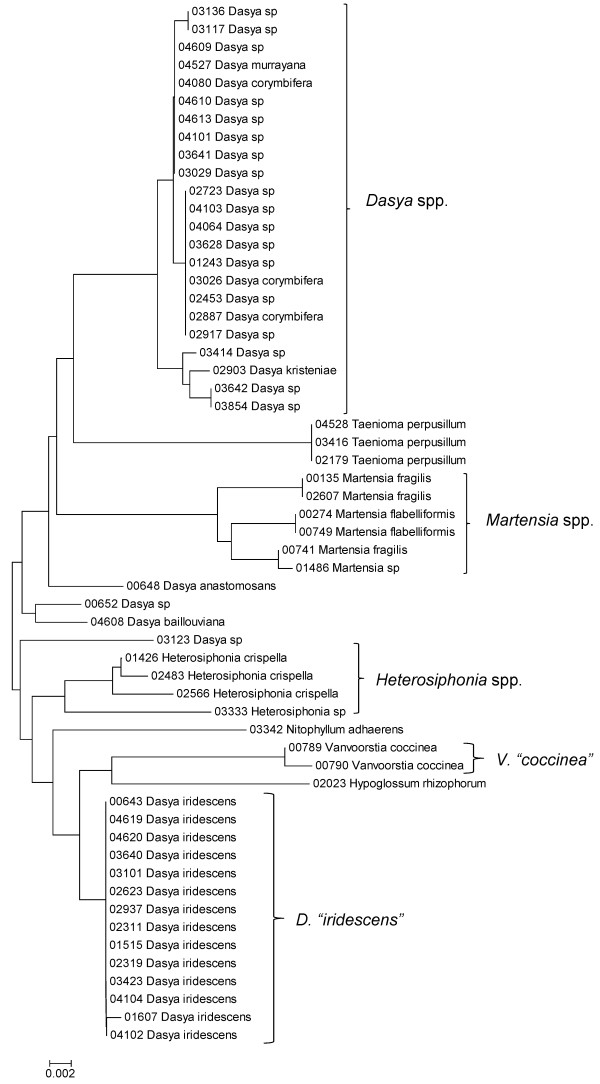
**Neighbor-joining tree of UPA sequences of the Dasyaceae, Delesseriaceae and Sarcomeniaceae of the order Ceramiales**. Sequence diversity based on the UPA plastid marker for surveyed Rhodophyta specimens belonging to the families Dasyaceae, Delesseriaceae and Sarcomeniaceae of the order Ceramiales. Neighbor-joining tree is based on the Maximum Composite Likelihood nucleotide model in MEGA 4.0. Scale bar = substitutions per site.

**Figure 33:**
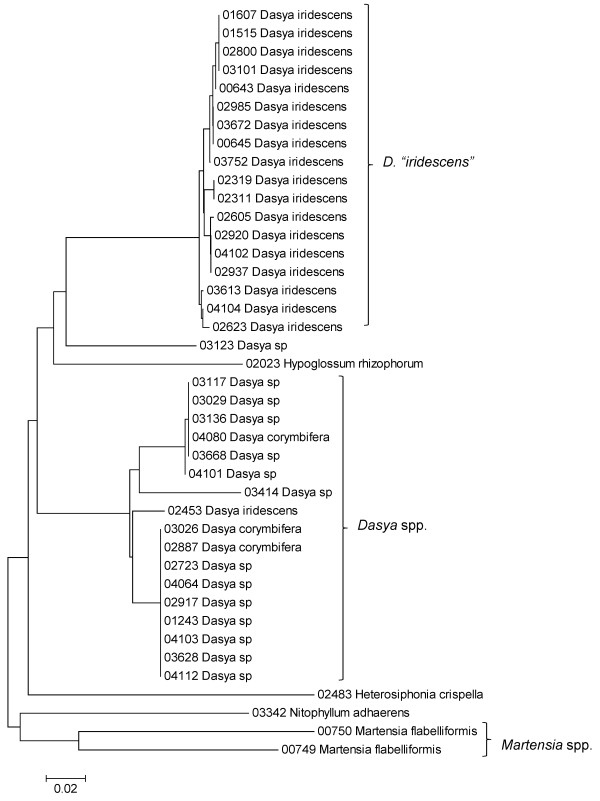
**Neighbor-joining tree of COI sequences of the families Dasyaceae, Delesseriaceae and Sarcomeniaceae of the order Ceramiales**. Sequence diversity based on the COI mitochondrial marker for surveyed Rhodophyta specimens belonging to the families Dasyaceae, Delesseriaceae and Sarcomeniaceae of the order Ceramiales. Neighbor-joining tree is based on the Maximum Composite Likelihood nucleotide model in MEGA 4.0. Scale bar = substitutions per site.

**Figure 34:**
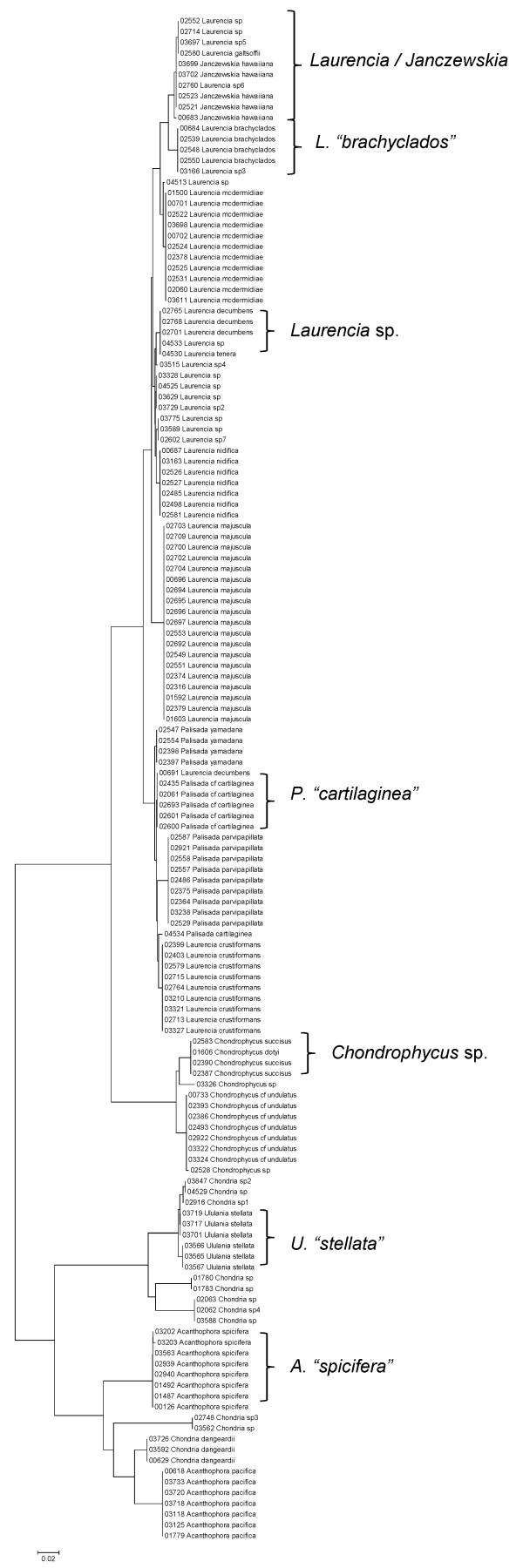
**Neighbor-joining tree of LSU sequences of the family Rhodomelaceae (part 1) of the order Ceramiales**. Sequence diversity based on the LSU nuclear marker for surveyed Rhodophyta specimens belonging to the family Rhodomelaceae (primarily the *Laurencia *complex) of the order Ceramiales. Neighbor-joining tree is based on the Maximum Composite Likelihood nucleotide model in MEGA 4.0. Scale bar = substitutions per site.

**Figure 35:**
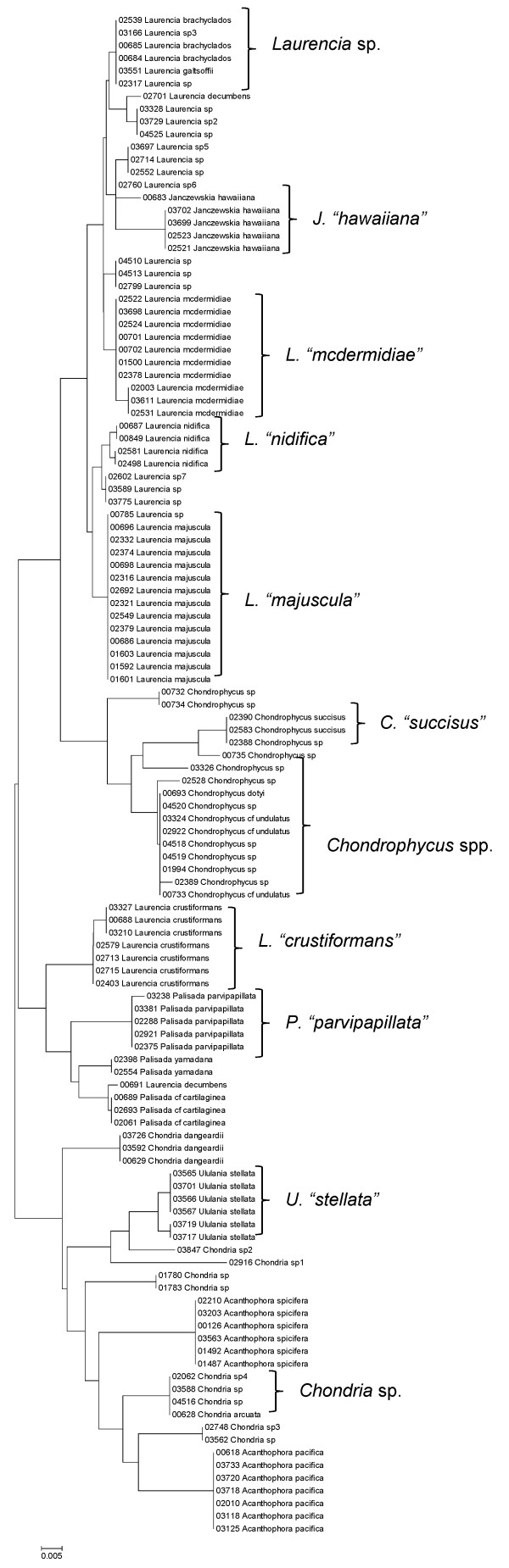
**Neighbor-joining tree of UPA sequences of the family Rhodomelaceae (part 1) of the order Ceramiales**. Sequence diversity based on the UPA plastid marker for surveyed Rhodophyta specimens belonging to the family Rhodomelaceae (primarily the *Laurencia *complex) of the order Ceramiales. Neighbor-joining tree is based on the Maximum Composite Likelihood nucleotide model in MEGA 4.0. Scale bar = substitutions per site.

**Figure 36:**
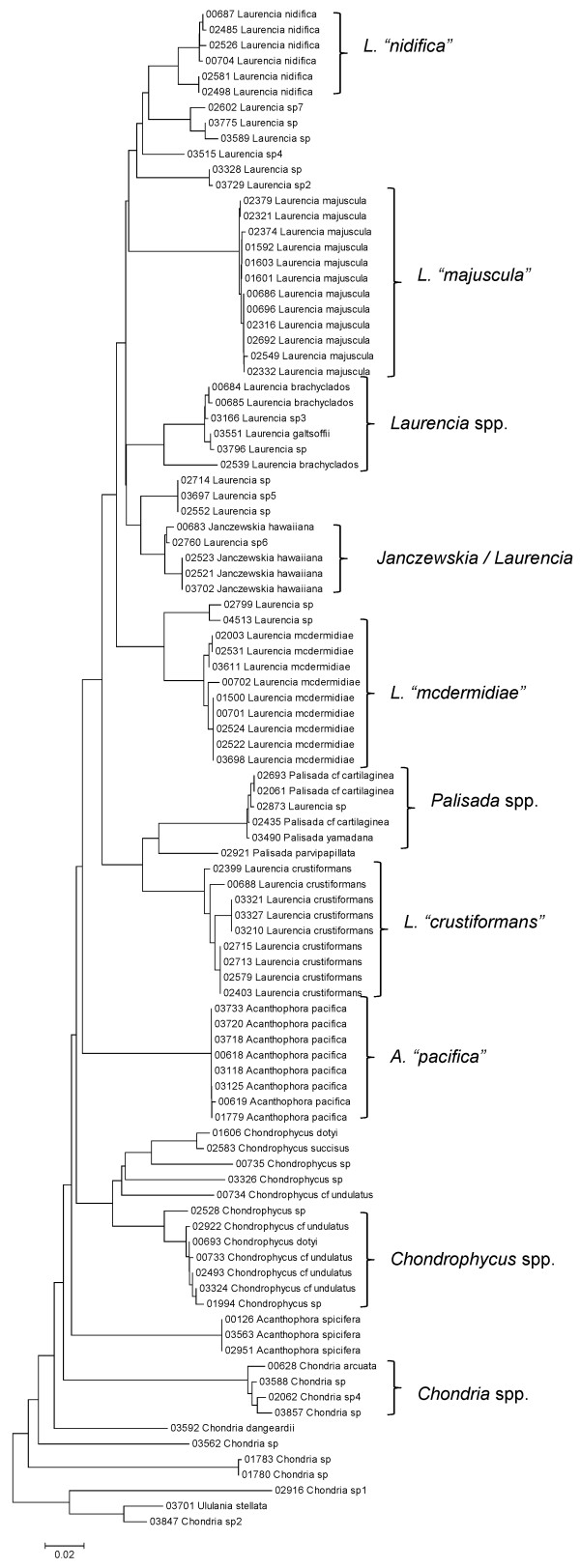
**Neighbor-joining tree of COI sequences of the family Rhodomelaceae (part 1) of the order Ceramiales**. Sequence diversity based on the COI mitochondrial marker for surveyed Rhodophyta specimens belonging to the family Rhodomelaceae (primarily the *Laurencia *complex) of the order Ceramiales. Neighbor-joining tree is based on the Maximum Composite Likelihood nucleotide model in MEGA 4.0. Scale bar = substitutions per site.

**Figure 37:**
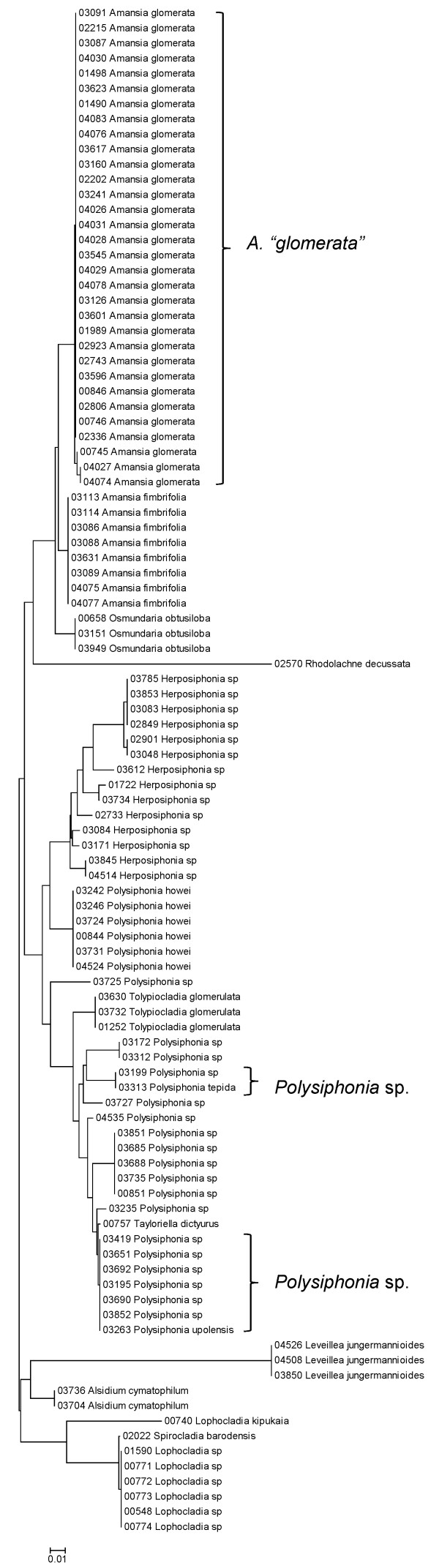
**Neighbor-joining tree of LSU sequences of the family Rhodomelaceae (part 2) of the order Ceramiales**. Sequence diversity based on the COI mitochondrial marker for surveyed Rhodophyta specimens belonging to the family Rhodomelaceae (excluding the *Laurencia *complex) of the order Ceramiales. Neighbor-joining tree is based on the Maximum Composite Likelihood nucleotide model in MEGA 4.0. Scale bar = substitutions per site.

**Figure 38:**
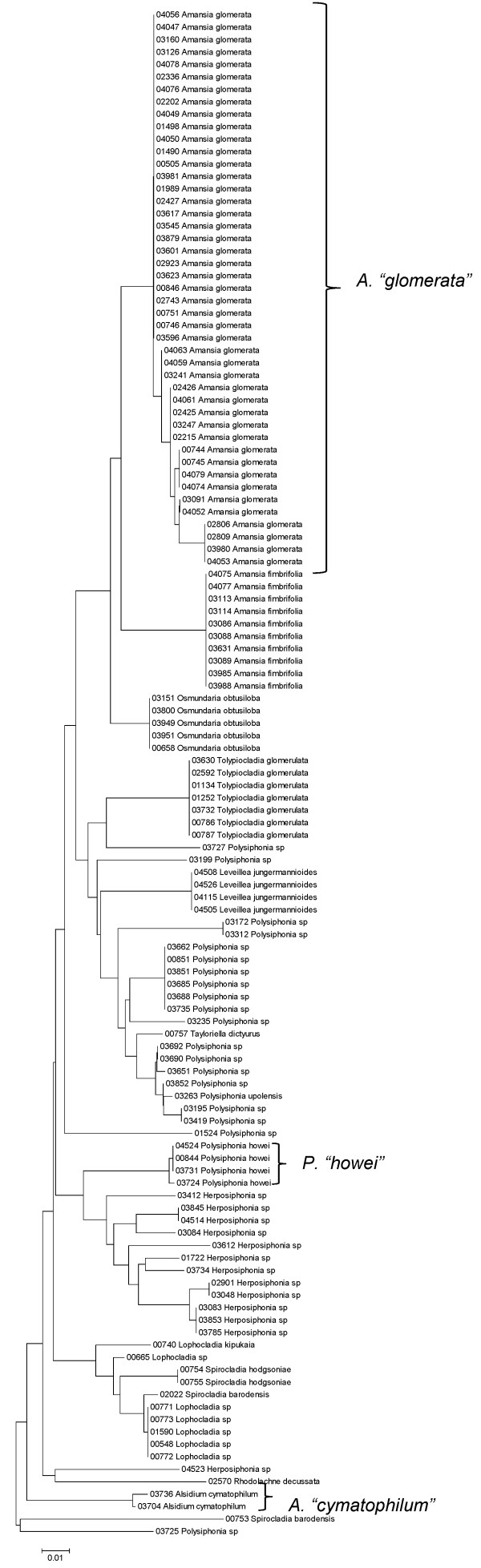
**Neighbor-joining tree of UPA sequences of the family Rhodomelaceae (part 2) of the order Ceramiales**. Sequence diversity based on the COI mitochondrial marker for surveyed Rhodophyta specimens belonging to the family Rhodomelaceae (excluding the *Laurencia *complex) of the order Ceramiales. Neighbor-joining tree is based on the Maximum Composite Likelihood nucleotide model in MEGA 4.0. Scale bar = substitutions per site.

**Figure 39:**
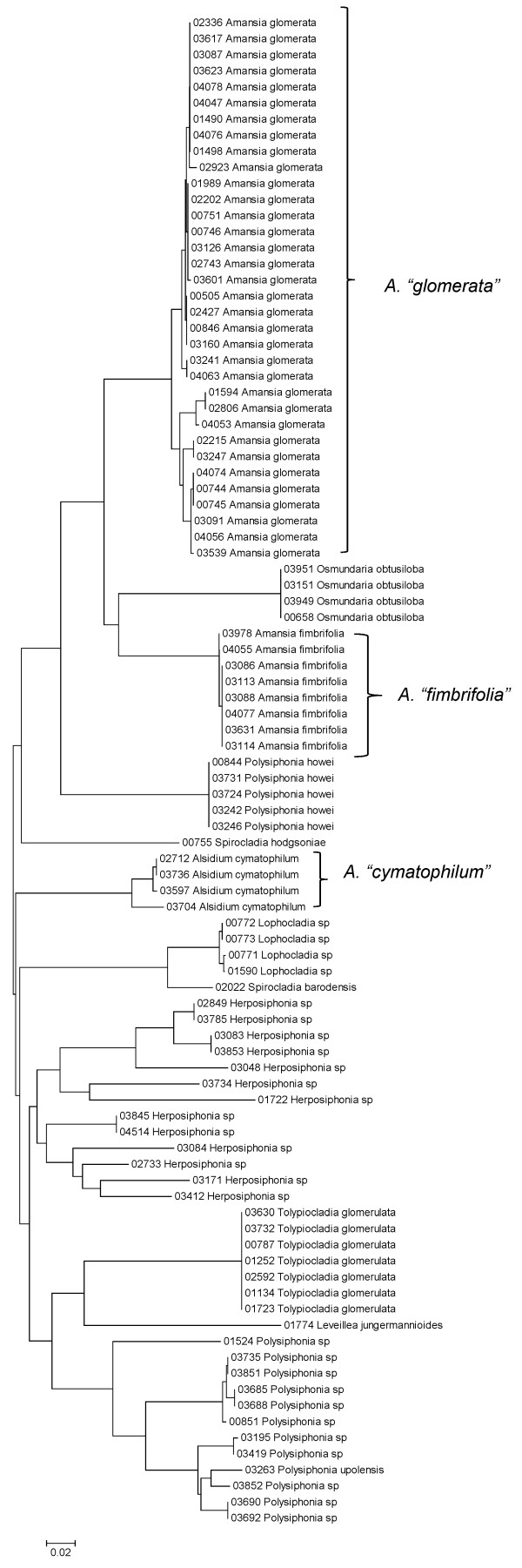
**Neighbor-joining tree of COI sequences of the family Rhodomelaceae (part 2) of the order Ceramiales**. Sequence diversity based on the COI mitochondrial marker for surveyed Rhodophyta specimens belonging to the family Rhodomelaceae (excluding the *Laurencia *complex) of the order Ceramiales. Neighbor-joining tree is based on the Maximum Composite Likelihood nucleotide model in MEGA 4.0. Scale bar = substitutions per site.

Some trends of marker utility are evident. Overall, a pattern of #LSU sequences > #UPA sequences > #COI sequences was observed in a number of instances, including for the Acrosymphytales, Gigartinales, Nemastomatales, Peyssonneliales, Plocamiales and Pihiellales (Figures 16, 17 and 18, Additional file 3), the Gelidiales, Gracilariales and Halymeniales (Figures 19, 20 and 21, Additional file 3), and many of the Ceramiales (Figures 28, 29, 30, 31, 32 and 33, Additional file 3). In contrast to this, the large number of COI sequences for the Bonnemaisoniales (Figure 12) relative to LSU (Figure 10) and UPA (Figure 11) is due to targeting of the COI region for a mtDNA-based phylogeographic study [43]. Overall, amplification and sequencing success was much higher for the LSU and UPA markers than for the COI marker, based on the laboratory protocols employed in this study.

A number of taxa were represented by multiple clusters of sequences according to one or more of the markers, including *Hydrolithon reinboldii *(Corallinales) (Figures 13, 14 and 15), *Grateloupia filicina *(Halymeniales) (Figures 19, 20 and 21), *Dichotomaria marginata *(Nemaliales) (Figures 22 and 24), *Champia parvula *(Rhodymeniales) (Figure 27), *Spyridia filamentosa *(Ceramiales) (Figures 28, 29 and 30), *Martensia flabelliformis *and *M. fragilis *(Ceramiales) (Figures 31, 32 and 33), *Laurencia crustiformans *and *L. mcdermidiae *(Ceramiales) (Figures 35 and 36), and *Amansia glomerata *(Ceramiales) (Figures 37, 38 and 39). These clusters may be due to cryptic or incipient speciation, or phylogeographic structure, and these taxa should be investigated through detailed anatomical and morphological comparison of specimens (and comparison to the type specimens) as well as increased sampling for molecular analyses, in order to clarify which of these processes accounts for the diversity observed in the NJ trees.

Other taxa were identified that will require taxonomic attention to reconcile patterns of molecular diversity with currently applied taxonomic names. For example, the genera *Ahnfeltiopsis *(Gigartinales) (Figures 16 and 18), *Peyssonnelia *(Peyssonneliales) (Figures 16 and 17) and *Gelidium *(Gelidiales) (Figures 19 and 20) all have clusters of sequences for at least one marker with multiple species names applied. Along these same lines, some pairs of taxa were shown to possibly be conspecific according to all three molecular markers employed here (e.g. *Gracilaria eppihippisora */*G. salicornia*, and *G. coronopifolia */*G. dotyi *[Figures 19 and 20]).

### Phylogenetic analysis of concatenated UPA, LSU and COI sequences

**Figure 40:**
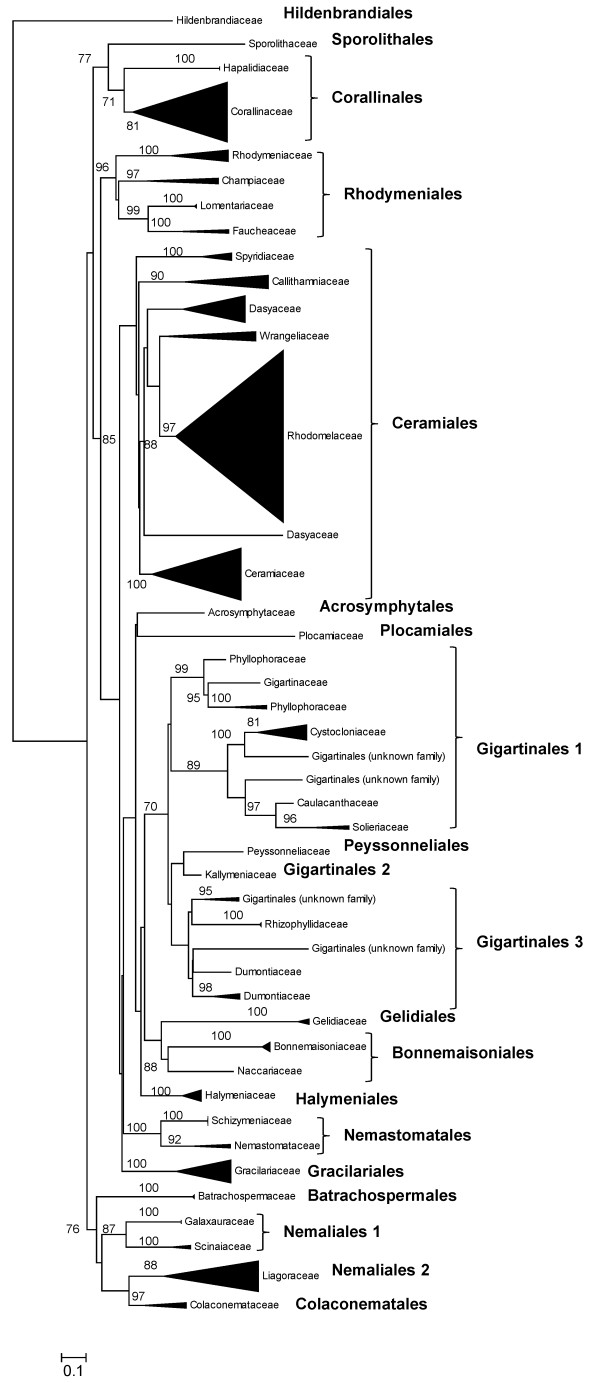
**RAxML analysis of concatenated LSU, UPA and COI sequences**. Maximum-likelihood tree of 284 florideophyte red algal samples based on an alignment of concatenated LSU, UPA and COI sequences. Taxa are summarized by family. Bootstrap values ≥70% are shown.

Although sequences for each of the three markers are short in length, when concatenated they yield 1,321 bp of data (with no indels and after trimming ambiguous regions) that can be used for phylogenetic analyses. A RAxML maximum likelihood analysis of sequence data for all accessions for which sequences of all three markers were available (*n *= 284 concatenated sequences) yielded a phylogenetic tree (Figure 40) that generally resembled current understanding of florideophyte red algal evolutionary relationships; however, bootstrap support was generally present only at and below the level of family. Of the 17 red algal orders included in the analysis, nine were supported as monophyletic, five were represented by only a single accession and thus did not have an associated bootstrap value, and three were not supported as monophyletic (the Ceramiales, Gigartinales and Nemaliales) (Figure 40). A total of 37 families were represented in the analysis, with 24 supported as monophyletic, nine represented by only a single accession, and four not supported as monophyletic (the Dasyaceae, Wrangeliaceae (topologically monophyletic but lacking bootstrap support), Phyllophoraceae, and Dumontiaceae) (Figure 40).

### Phylogeographic analyses of widespread taxa

**Figure 41:**
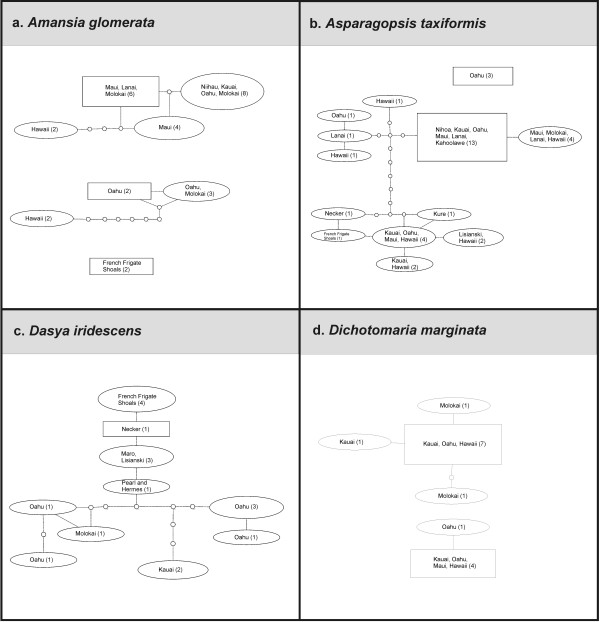
**Phylogeographic analysis of selected Hawaiian Rhodophyta**. Statistical parsimony networks (95% connection limit) of four Hawaiian red algal taxa for which multiple collections were analyzed (a - *Asparagopsis taxiformis*, b - *Ceramium dumosertum*, c - *Dasya iridescens*, d - *Amansia glomerata*). The number of sequences for each haplotype is given in parentheses. Rectangles represent the hypothesized ancestral haplotype. Small circles indicate hypothetical intermediate haplotypes with single nucleotide changes. Haplotype size is proportional to frequency (relative for each taxon).

Although our collecting strategy was to analyze approximately three specimens per taxon, several common taxa (as identified using [1] or more recent references listed in [44]) were represented in the survey collections by numerous sequences for the mitochondrial COI marker (*Amansia glomerata *(Figure 41a), *Asparagopsis taxiformis *(Figure 41b), *Dasya iridescens *(Figure 41c) and *Dichotomaria marginata *(Figure 41d)). Preliminary sequence analyses suggested that intraspecific variation was present for each of these, and thus they were employed as phylogeographic examples to explore whether general patterns are discernable for members of the Hawaiian red algal flora (Figure 41a-d).

#### Amansia glomerata

*A. glomerata *is widely distributed throughout the Hawaiian archipelago [1]. Statistical parsimony analysis yielded three distinct networks; one containing NWHI samples (French Frigate Shoals), and two containing MHI samples (Figure 41a; [10]).

#### Asparagopsis taxiformis

This species is sought after as a food source in the Hawaiian culture [1]. Statistical parsimony analysis of COI sequences for this taxon yielded two networks formed at the 95% connection limit; one that contains three samples from the south shore of the island of Oahu (which possibly represent a recent introduction), belonging to Lineage 4 (*sensu *[43,45]), and the remainder forming a large network that is broadly divided into Lineage 1 (widespread throughout the MHI and Nihoa) and Lineage 2 (NWHI and MHI in distribution) (Figure 41b; [43]).

#### Dasya iridescens

A single network was formed by statistical parsimony analysis containing both MHI and NWHI samples (Figure 41c). The NWHI samples were closely related to one another and separated from MHI samples. Additionally, greater nucleotide diversity was present for MHI samples, especially those from the island of Oahu.

#### Dichotomaria marginata

Two networks were formed by statistical parsimony analysis, both of which contained only MHI samples (Figure 41d). Samples from the island of Molokai formed two distinct haplotypes in the most populated network, while samples from the other islands (Kauai, Oahu, Maui and Hawaii) were represented in the most geographically diverse haplotypes (Figure 41d).

In contrast to these findings, a number of taxa were analyzed for which multiple sequences were obtained (for at least two markers), but with little or no sequence diversity across the sampled range (based on *p*-distances, and reported as sequence identity) (Table 1). These species - *Acanthophora pacifica*, *Coelothrix irregularis*, *Haliptilon subulatum*, *Laurencia majuscula*, *Lithophyllum insipidum*, *Polysiphonia howei *and *Tolypiocladia glomerulata *- are all presumed to be native to the Hawaiian Islands [1] and represent three different orders of red algae (the Ceramiales, Corallinales and Rhodymeniales). These examples demonstrate that not all widespread Hawaiian red algal taxa have high intraspecific divergence, and hence lend further support to *Asparagopsis taxiformis*, *Amansia glomerata *and *Dichotomaria marginata *consisting of species complexes in the Hawaiian Islands.

**Table 1:** Hawaiian Rhodophyta with low intraspecific divergence

Taxon	Represented range	COI*	UPA*	LSU*
*Coelothrix irregularis *(Rhodymeniales)	Oahu, Molokai, Maui, Hawaii	0.996-1.000 (5)	1.000 (5)	1.000 (5)
*Lithophyllum insipidum *(Corallinales)	Oahu, Lanai, Hawaii	0.991-0.995 (3)	0.980-0.997 (4)	0.991-1.000 (6)
*Haliptilon subulatum *(Corallinales)	Kauai, Oahu, Molokai, Hawaii	0.990-1.000 (7)	0.997-1.000 (4)	1.000 (6)
*Tolypiocladia glomerulata *(Ceramiales)	Oahu, Molokai, Maui, Hawaii	1.000 (7)	1.000 (7)	1.000 (3)
*Polysiphonia howei *(Ceramiales)	Oahu, Maui, Hawaii	1.000 (5)	0.997-1.000 (4)	1.000 (6)
*Acanthophora pacifica *(Ceramiales)	Oahu, Molokai, Maui, Kahoolawe, Hawaii	0.998-1.000 (8)	1.000 (7)	1.000 (7)
*Laurencia majuscula *(Ceramiales)	Laysan, French Frigate Shoals, Kauai, Oahu, Molokai, Lanai	0.995-1.000 (12)	1.000 (14)	1.000 (19)

*divergences reported as sequence identity (number of sequences in parentheses following sequence identities)

## Discussion

The Hawaiian Rhodophyta Biodiversity Survey yielded only a few new taxonomic records for the Hawaiian Islands, but many new distributional records. The flora has been remarkably well-studied from a morphological and anatomical perspective, most notably by Abbott [1], and many of her designations are supported by our molecular investigations. However, many taxa have also been revealed to be in need of in-depth taxonomic study based on our molecular survey data due to instances of suspected cryptic or incipient speciation (e.g. *Amansia glomerata *and *Spyridia filamentosa*; see results for full list), or conspecificity (e.g. *Gracilaria eppihippisora *and *G. salicornia*). As such, our survey data provide a unique opportunity to flag Hawaiian red algal taxa for further study. Although identification of these taxonomic issues, rather than full resolution, was the goal of the Hawaiian Rhodophyta Biodiversity Survey, several of these flagged taxa have been investigated in detail under the auspices of the project (e.g. [8,10,43]), and we hope that other researchers will be able to make use of the data for similar purposes, for it is detailed taxonomic studies that will ultimately lead to a floristic revision of the Hawaiian red algae. Also, additional taxonomic expertise for some of the lineages of red algae investigated during the project (in particular, for many of the Ceramiales), will be needed for taxonomic resolution. However, despite adding relatively few new taxa to the species list, we have substantially updated the reported range of many taxa, with 196 new island-level distributional records included from our sampling records.

The vast majority of accessions analyzed were from marine and brackish locations (1,928, or 99%), while 16 were from freshwater habitats and two were from terrestrial locations. This breakdown is close to the accepted consensus that, worldwide, approximately 97% of red algal species are marine [46].

The three molecular markers chosen for diversity assessment of our red algal collections (nuclear LSU, mitochondrial COI and plastid UPA) differ markedly in both their degree of conservation and ease of amplification/sequencing, based on the protocols that were applied to our survey samples. Since the Hawaiian Rhodophyta Biodiversity Survey encompassed an evolutionarily diverse set of samples, and we aimed to generate a large number of sequences (i.e. >2,000), we were constrained by the need for minimal variations of protocol for each marker (including primer design, PCR cocktail components/concentrations and amplification conditions). The LSU and UPA markers were sequenced without modification (i.e. according to the primers and conditions described in [27] and [29] respectively), and these first two markers yielded the highest and second highest overall number of sequences, respectively. In contrast, the COI marker required the design of an additional reverse primer, and yielded the lowest number of sequences. Ease of data acquisition and performance characteristics of each of the three markers are further examined and discussed in [47], as well as below.

All clean sequences (those with clearly readable chromatograms that could be assembled with confidence) were retained in HADB, regardless of whether or not they corresponded to the taxon under study; contaminant sequences (e.g. from epiphytes) were re-labelled as such and given new accession numbers. All three markers yielded some contaminant sequences, but the UPA marker yielded the highest number (30), followed by LSU (15) and COI (7).

The LSU marker was the simplest for which to reliably obtain clean sequence data; only a single pair of primers was needed, and most samples were successfully sequenced on the first attempt. The disadvantage of the LSU marker, however, was a lack of reliable species-level resolution (e.g. Figure 21, Nemaliales LSU sequence data show no divergence between some members of the genera *Akalaphycus*, *Ganonema*, *Izziella*, *Liagora *and *Trichogloea*). A recent investigation of the characteristics and phylogenetic "performance" of the LSU, UPA and COI markers based on this same data set confirmed that the LSU marker is the most conserved of the three, that it was the easiest for which to obtain sequence data, and illustrated that florideophyte red algal sequence data for the marker became saturated at F84 distances of approximately 0.30 (although there was variation when individual orders were examined) [47]. The LSU marker was included in the survey to provide representation from the nuclear genome (both the UPA and COI are organellar markers). However, given that the level of resolution for the LSU marker differs so strongly from the other two markers (Figure 4), it would be difficult to recommend its inclusion in future biodiversity surveys.

The UPA marker, which was first described in 2006 [28] and tested for a selection of algal lineages in 2007 [29], can be amplified and sequenced for most cyanobacterial and plastid-containing algal lineages, with only a few genus-level exceptions noted to date (e.g. *Cladophora*, *Symbiodinium*). This universality is exceptional among algal primers, and lends great potential for the use of this marker in assessment of environmental samples that contain multiple algal lineages [48]. For this reason, we believe it is important to continue to generate framework data for this marker for as many algal representatives as possible. Recent work has shown that the UPA marker falls between in the COI and LSU markers in terms of ease of data acquisition, that it is quite conserved (although slightly less so than LSU), and that saturation is not an issue for any group of the florideophyte red algae based on UPA sequences [47].

The COI marker was somewhat more difficult to amplify and sequence across red algal lineages, and two combinations of primers were used to obtain the 639 sequences reported here (although new primers have been designed recently that are generally more successful; G.W. Saunders, pers. comm.). Others have reported requiring up to seven primer combinations to obtain COI sequences for samples within a single family of red algae [49]. However, the COI marker is widely recognized as the DNA barcode for red algae [9,41,49], and as such, provides a useful sequence data set due to the immediate comparative power of COI sequences against the 11,925 red algal DNA barcode sequences in the Barcode of Life Data Systems (BOLD) v.2.5 database (accessed 05 November 2010 [50]). This trade-off between universal amplification and resolution has been noted in the past [42,49]. We recently reported that these COI florideophyte red algal sequence data were the most difficult of the three markers to obtain (i.e. low ease of acquisition), but that the COI marker is the least conserved (and is especially variable at the third codon position), and that saturation of the entire data set occurs at F84 distances of approximately 0.11 (although this number varies substantially depending on which order is being examined) [47].

Maximum likelihood analysis of the concatenated LSU+UPA+COI sequences yielded a phylogenetic tree that included support for most of the florideophyte red algal orders and families included in the data set (i.e. 3/17 orders and 4/37 families were not recovered as monophyletic; Figure 40). A number of explanations may be responsible for the lack of full resolution in the phylogeny, including insufficient sequence data [47,51], insufficient taxonomic representation, or unresolved identifications of some specimens. Verbruggen *et al*. [51], in their recent research on the data requirements for resolving the red algal tree of life, indicated that up to 2.8 × 10^5 ^nucleotides of sequence data may be required to resolve some regions of the phylogeny, which is orders of magnitude more data than that provided by our concatenated three-marker data set. We [47] preliminarily evaluated the phylogenetic performance of the three markers with the same data set by assessing changes in neighbour-joining bootstrap support with the addition of marker data (progressing from UPA, to UPA+LSU, to UPA+LSU+COI), and also concluded that the fully concatenated phylogeny was supported largely at the family and ordinal level, with higher level relationships not resolved. Nonetheless, the sequences of the three markers will make a valuable addition to the red algal species assignment framework of data, and in concatenated form are useful for examining phylogenetic relationships at lower taxonomic levels.

Considering the taxa studied using statistical parsimony analysis (Figure 41), the debate arises as to whether observed breaks in networks represent strong phylogeographic signal or species boundaries. It has been suggested that the 95% connection limit under statistical parsimony, which is commonly used in phylogeographic studies, is a useful criterion for indicating biological species boundaries [52]. Applying this criterion to our analyses suggests that cryptic species may be common in the Hawaiian red algal flora. Only one of the four taxa investigated formed a single haplotype network at the 95% connection limit (*Dasya iridescens*), while the remaining taxa formed two (*Asparagopsis taxiformis *and *Dichotomaria marginata*) or three networks (*Amansia glomerata*). If this pattern holds true for a number of other Hawaiian red algal taxa (which will only be determined as more taxa are studied in depth), then there may be many more species than currently recognized in the flora. Sorting out phylogeographic signal from species boundaries requires an understanding of the limits of phenotypic plasticity for each case. Most of our identifications were made, at least preliminarily, based on morphology and anatomy. We largely relied on the detailed descriptions of Abbott [1] but supplemented these with more recent or taxon-specific studies that were relevant to each taxon (a list of this additional literature can be found in [44]). Nonetheless, lately there have been a number of cases in the phycological literature where cryptic species complexes have been revealed through molecular data, and in a number of these instances other characters (morphological, anatomical, life history, physiological, etc.) have later been identified that allowed recognition of the species on grounds other than molecular data, e.g. [49,53]. More detailed phylogeographic, morphological/anatomical and life history analyses of suspected species complexes will be necessary for confirmation, but the DNA sequences from the present survey provide a valuable starting point for identification of these phenomena, and pave the way for taxonomic revision of the floristic checklist.

Interestingly, not all common Hawaiian Rhodophyta species have notable intraspecific sequence variation. Eight taxa representing three different orders (Table 1) were reported to have little to no sequence divergence for the three markers sequenced. Although these taxa require stronger sampling for confirmation and resolution of these trends, one important finding from the survey is that phylogeographic patterns are likely to vary, even for taxa considered to be native to the Hawaiian Islands, and that histories of individual taxa need to be studied (e.g. time since colonization or speciation, colonization order of the islands, interisland dispersal vectors, introduction events from other regions) to obtain a fuller understanding of intraspecific molecular patterns. It does not seem to be the case that increased within-taxon sampling will always reveal obvious phylogeographic patterns. This difference could be due to many factors, including the length of time since colonization of the species (or speciation, in the case of endemic taxa), ease of dispersal, dispersal vectors and type of life history (i.e. whether there is an easily spread life history stage, such as a floating, filamentous form), habitat and physicochemical parameter tolerances.

Ekrem et al. [54] emphasized that DNA barcode data will only ever be as useful as the reference library of sequences against which comparisons are made. This is a compelling argument for support of biodiversity surveys of entire floras, such as this one, that attempt to account for the diversity of a region with both morphological and molecular sequence characterizations, and that make these data available to others in easily-accessible formats for comparisons. Zhang et al. [55], however, rightly point out that sample sizes for most DNA barcode studies are insufficient to assess genetic diversity of a species, and the level of intraspecific sampling for our project is no exception. Given the limited time and funds for the survey, we aimed to strike a balance between representation of as many taxa as possible and sufficient coverage of common taxa to assess intraspecific diversity. We emphasize that the patterns discussed here are largely preliminary, and that a more thorough examination is warranted to reveal the full story.

The value of the Hawaiian Rhodophyta Biodiversity Survey is multifaceted: through this project we have generated baseline molecular diversity data for much of the flora, revealed taxa requiring further systematic investigation (both in terms of possible cryptic species that have not been previously recognized, and morphologically/molecularly similar taxa that may be synonymous), begun to elucidate phylogeographic patterns for Hawaiian red algae, revised the species checklist for Hawaiian Rhodophyta, and reported a number of new records (both taxonomic and distributional). All of these data (our own collections plus those from BISH), as well as photographs and micrographs, specimen details and collecting site information, are available *via *the publicly accessible Hawaiian Algal Database [56], and the data have also been shared with the Global Biodiversity Information Facility [57] and GenBank [58]. Voucher specimens and the DNA extract library are available to other researchers, enhancing the long-term value of the survey collections. The value of the survey will also increase as other studies make use of the data and our initial observations are developed into additional projects.

## Conclusions

Here we describe the principal findings of the Hawaiian Rhodophyta Biodiversity Survey, a four-year effort to inventory and assess the species of red algae from the Hawaiian Islands. Numerous new taxonomic and distributional records resulted from the collections, which allowed for an updated taxonomic checklist of Hawaiian red algae. Three molecular markers (one each from the nucleus, plastid and mitochondrion) were sequenced for numerous collections, and the data from each marker compared to establish baseline molecular diversity profiles for the flora. A number of cases were highlighted where the molecular data helped to identify taxa or groups of taxa in need of systematic revision, illustrating the value of constructing this baseline molecular diversity data set. In the case of widespread and common species, the sequence data were also helpful in beginning to elucidate phylogeographic patterns, which differed substantially depending on the taxon. A maximum likelihood analysis based on concatenated sequence data for 17 red algal orders yielded a tree topology that is largely consistent with current understanding of red algal phylogeny, but with lower support at deeper nodes, indicating that the DNA barcode-like markers have phylogenetic utility at and below the ordinal level. The value of each of the markers for biodiversity surveys was also assessed, and recommendations made for inclusion or exclusion in future surveys of algal floras.

## Methods

### Sampling strategy

Samples were field-collected or subsampled from accessioned material at the Bernice P. Bishop Museum's *Herbarium Pacificum *(BISH). Annual collecting expeditions to the islands of Kauai, Oahu, Molokai, Maui, Lanai and Hawaii were undertaken to survey nearshore coastal areas and streams. Samples from the Main Hawaiian Islands of Niihau and Kahoolawe were processed as they were opportunistically obtained, as were those from the Northwestern Hawaiian Islands. We aimed to collect three specimens per species, although there was variation around this number due to rare or, conversely, extremely widespread taxa.

### Sample processing and analysis

Field-collected samples were processed as described in Sherwood [43]. Samples were examined using light microscopy (Olympus BX-41 compound microscope [Olympus, Center Valley, PA, USA] and a Zeiss SteREO Discovery.v12 stereomicroscope, Carl Zeiss Microimaging Inc., Göttingen, Germany). Identifications were made using the most current sources, with [1] forming the foundation for identifications and more recent primary literature being added where appropriate [3-6,40]. Material analyzed from BISH was labelled as per the assigned identification on the herbarium sheet, except in cases of obvious mis-identification or where microscopic examination revealed the sample to belong to a different taxon (herbarium sheets were subsequently annotated to reflect these changes).

DNA was extracted from field-collected and herbarium material following the methods described in [27]. All DNA extracts are housed in the Hawaiian Algal DNA Library at the University of Hawaii at Manoa, which is maintained at -80°C in the Sherwood Laboratory. Duplicate DNA extracts of material from herbarium sheets housed at BISH are maintained at Bishop Museum's Pacific Center for Molecular Biodiversity. Three short DNA markers were amplified and sequenced for as many of the collections as possible; these markers were selected to represent different genomes within the cell (i.e. nucleus - part of the 28 S rRNA gene (LSU), plastid - the Universal Plastid Amplicon (UPA) [28,29], and mitochondrion - the cytochrome oxidase I barcode region (COI) [9]). PCR primers and cycling conditions used for each of the markers are referenced in [27], with information on additional LSU and COI primer derivation as follows.

We aligned the entire GenBank content for Rhodophyta LSU (>650 sequences ranging from ~800-2700 bp) and designed primers to amplify a central portion where most of the data overlapped. These primers, nu28SF (5'-GGAATCCGCYAAGGAGTGTG-3'; Tm = 56.9°C) and nu28SR (5'-TGCCGACTTCCCTTACCTGC-3'; Tm = 59.7°C) produced an amplicon of variable length; 580-650 bp depending on the taxon. Cycling conditions for this amplicon were as follows: 94°C for 2 min, 40 cycles of 94°C for 20 s, 55°C for 30 s, 72°C for 50 s, and a 5 min final extension at 72°C.

**Figure 42:**
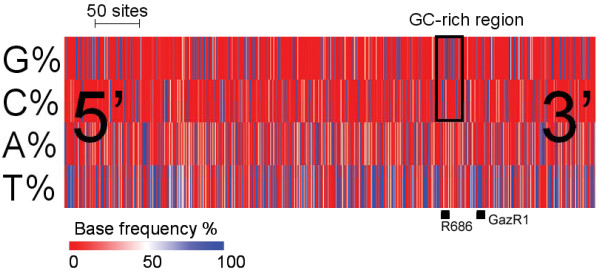
**Representation of base frequencies along 663 nt of Rhodophyta COI**. Nucleotide frequency values (G, C, A, and T from top to bottom row) for a given site are reported on a red to blue scale (0 to 100% conservation). The positions of GazR1 [9] and our nested primer, R686, are displayed and the GC-rich region in which R686 lies is highlighted. The numerous polyTs in COI 3'end (four or more successive thymidines), potentially affecting GazR1 specificity, can be seen as thick blue bands. The multiple alignment used to create this figure contained accessions EF136591-EF434941, Z47547 NC_001677 and AF118119 (Gigartinales and Gracilariales), Z48930, D89861 and NC_000887 (Cyanidiales), and AF114794 and NC_002007 (Bangiales), which were selected for their length, extending past GazR1. The region shown depicts sites 205-868 of the *Cyanidium caldarium *(Z48930) complete COI sequence.

We experienced inconsistent sequencing results for COI using the published primer pair GazF1-GazR1 [9]. For the same PCR product, reverse chromatograms (GazR1) were regularly lower in quality than the forward reads (GazF1). We suspect that GazR1 polyA 3'end (5'-ACTTCTGGATGTCCAAAAAAYCA-3'; Tm = 53.9°C) poses specificity problems, mispriming and/or mismatching with several polyTs located near the GazR1 priming site. A search for suitable priming sites in the first 863 bp of COI did not reveal any single 10-15-bp unit fully conserved across red algal taxa, making the design of universal primers a challenge. However, we identified a GC-rich region nested upstream of GazR1 containing successive CCs and GGs, theoretically offering stronger specificity (Figure 42). We designed an alternate reverse primer within this region, R686 5'-CCACCWGMAGGATCAA-3' (Tm = 49.7°C). GazF1-R686 produces an amplicon of 616 bp (vs. 720 bp for GazF1-GazR1) that still contains the 500 bp required by BOLD for barcode designation [50]. We prioritized amplifying with GazR1 for the longer amplicon it produces, and used R686 to re-sequence low quality reads and/or re-attempt amplification when the former failed. PCR thermal profiles were optimized for COI with GazF1-R686: 94°C for 1.5 min, 40 cycles of 94°C for 30 s, 47°C for 40 s, 72°C for 40 s, and a 5 min final extension at 72°C. PCR products were purified and sequenced, and resulting chromatograms manually assembled according to previously published protocols [27].

### Data storage and dissemination

All relevant project data (photographs and micrographs, locality information, DNA sequences, identifications, voucher type and archival information) were entered into the Hawaiian Algal Database (HADB); a database that was specially designed and constructed for the project to allow organization and accessibility of the data. HADB is internet accessible at http://algae.manoa.hawaii.edu and is fully described in [59].

### Data analyses

#### Mapping of GPS data

Collection locality data (GPS points) were downloaded from HADB and plotted on a map of the Hawaiian Islands using GPS Visualizer [60]. All coastal, freshwater and terrestrial MHI points were included, while those from the NWHI were excluded since most were only at island-scale resolution.

#### Construction of DNA sequence diversity trees

Sequences for each marker were downloaded from HADB and aligned using Clustal X [61]. Neighbor-joining trees of sequences for the UPA, COI and LSU markers were constructed based on the maximum-likelihood composite model in MEGA v.4 [62]. Separate trees were constructed for each marker, and for each red algal order (or groups of related orders when sample numbers were low enough to still visualize taxon labels on trees), or by family (in the case of the order Ceramiales, which accounts for approximately half of the Hawaiian red algal diversity). Summary labels were added to the trees to indicate the following: a) previously identified lineages of species from the literature (in these cases the citations are given in the figure legends), b) groups of sequences that, to the best of our knowledge, belong to the same species although some sequence divergence is present (e.g. *Chroodactylon *"*ornatum*"; Figure 5), c) groups of sequences that do not have a consistent taxonomy but nonetheless likely represent a single taxon (e.g. *Platoma *sp.; Figure 16), and d) groups of sequences that do or do not have a consistent taxonomy and likely represent multiple species (e.g. *Peyssonnelia *spp.; Figures 16 and 17).

#### Phylogenetic analysis of concatenated sequence data

All accessions for which sequences of all three markers were available (*n *= 284) were concatenated using the software package APE [63] with R [64]. Fast evolving loops of LSU (indel rich regions) were removed because sequence homology could not be ascertained with confidence, leaving only stems for this marker. Similarly, a very short segment of UPA, which aligned ambiguously on the scale of the entire data set, had to be removed. Concatenated sequences were analyzed with the rapid bootstrap (RBS) and maximum likelihood (ML) search algorithm of the RAxML Blackbox server http://phylobench.vital-it.ch/raxml-bb/[65]. RAxML uses a hill-climbing algorithm that searches the tree space using Lazy Subtree Rearrangement [66] while allowing specification of partitioned models useful for concatenated data sets. An exploratory analysis of the sequences can be found in [47] which demonstrates the varying levels of substitution saturation and substitution rates for UPA, LSU, and all three codon positions of the COI marker. Following this five-partition scheme, the RAxML analysis was launched with a GTR+G_4 _model and branch length optimization per partition with the default 100 RBS. The returned ML tree was summarized by family and order in MEGA [62] and RBS values ≥70% were illustrated.

#### Phylogeographic analyses

Phylogeographic patterns for *Amansia glomerata*, *Asparagopsis taxiformis*, *Dichotomaria marginata *and *Dasya iridescens *(representatives of widespread Hawaiian red algae with evident structuring at the molecular level) were estimated using COI marker sequences (which is commonly used for phylogeographic inference, e.g. [24] and the statistical parsimony method of Templeton *et al*. [67] using TCS v.1.21 [68], employing a 95% connection limit and treating gaps as a 5^th ^state. Sequence divergences for the widespread taxa *Acanthophora pacifica*, *Champia parvula*, *Coelothrix irregularis*, *Haliptilon subulatum*, *Laurencia majuscula*, *Lithophyllum insipidum*, *Polysiphonia howei *and *Tolypiocladia glomerulata *were calculated based on *p*-distances using MEGA v.4 [62] and reported as percent identities.

## Authors' contributions

ARS, AK, KYC, and TS conducted field work and processed collections (including identification, voucher archival, photography/photomicroscopy and DNA sequencing). ARS, AK and KYC performed the data analyses. TS performed primer design and troubleshooting for the LSU and COI-R686 primers. NW constructed and maintained the Hawaiian Algal Database for project data management, and provided data downloads. ARS and GGP conceived the study, designed the project, obtained funding and coordinated the work. ARS wrote the initial draft of the manuscript. All authors read and approved the final manuscript.

## Supplementary Material

Additional file 1 **List of collection locations**. Collection locations for samples surveyed as part of the Hawaiian Rhodophyta Biodiversity Project. Islands are listed in alphabetical order. Geographical coordinates are listed in decimal degrees. Click here for file

Additional file 2 **Taxonomic checklist and new records**. Taxonomic checklist and new records from the Hawaiian Rhodophyta Biodiversity Project. Taxon records are arranged by order. New taxon and island records are indicated in bold. Click here for file

Additional file 3 **Sequence accessions**. Hawaiian Algal Database accession numbers for all sequences analyzed as part of the Hawaiian Rhodophyta Biodiversity Survey. Accessions are given as superscripts for each of the three markers (N = nuclear LSU, M = mitochondrial COI, P = plastid UPA). Click here for file
