# Supplementary material for: The Hawaiian Rhodophyta Biodiversity Survey (2006-2010): a summary of principal findings
Source: BMC Plant Biol. 2010 Nov 22;10:258. doi: 10.1186/1471-2229-10-258 (PMC3012605; doi:10.1186/1471-2229-10-258)
Supplement: Additional file 1 — List of collection locations. Collection locations for samples surveyed as part of the Hawaiian Rhodophyta Biodiversity Project. Islands are listed in alphabetical order. Geographical coordinates are listed in decimal degrees. [file 1471-2229-10-258-S1.DOCX]

**Additional file 1.** List of collection locations.

| **Island ("Region" in HADB)** | **Site details** | **Latitude** | **Longitude** |
| --- | --- | --- | --- |
| French Frigate Shoals | Census of Marine Life CReefs cruise samples | 23.7616 | -166.169 |
| French Frigate Shoals | La Perouse Pinnacles | 23.7720 | -166.186 |
| French Frigate Shoals | Northwestern Hawaiian Islands (BISH samples) | 23.7443 | -166.142 |
|  |  |  |  |
| Hawaii | Ahalanui Park | 19.4680 | -154.832 |
| Hawaii | Akaka Falls State Park | 19.8565 | -155.162 |
| Hawaii | Anaehoomalu Bay | 19.9137 | -155.891 |
| Hawaii | Bay Front Beach | 19.7240 | -155.083 |
| Hawaii | Coconut Island (Hilo) | 19.7291 | -155.069 |
| Hawaii | Halape | 19.2679 | -155.252 |
| Hawaii | Hawaii Division of Aquatic Resources baseyard in Hilo | 19.7215 | -155.073 |
| Hawaii | Hiilawe Stream (Waipio Valley) | 20.0896 | -155.600 |
| Hawaii | Hilo Bay | 19.7249 | -155.080 |
| Hawaii | Hilo Bay (by Queen Liliokalani Gardens) | 19.7264 | -155.070 |
| Hawaii | Hilo River | 19.7284 | -155.087 |
| Hawaii | Honokohau | 19.6709 | -156.026 |
| Hawaii | Honomu | 19.8757 | -155.109 |
| Hawaii | Honuapo Bay | 19.0860 | -155.550 |
| Hawaii | Hookena Beach Park | 19.3788 | -155.897 |
| Hawaii | Isaac Hale Beach | 19.4581 | -154.842 |
| Hawaii | Island of Hawaii (general) |  |  |
| Hawaii | Kahaluu Beach | 19.5804 | -155.966 |
| Hawaii | Kailua (Kona) | 19.7342 | -156.058 |
| Hawaii | Kalapana | 19.3491 | -154.972 |
| Hawaii | Kapoho Point | 19.4983 | -154.819 |
| Hawaii | Kaupulehu | 19.8241 | -155.997 |
| Hawaii | Kawaihae Boat Harbor | 20.0396 | -155.831 |
| Hawaii | Keahole Point (Kona) | 19.7261 | -156.063 |
| Hawaii | Kealakekua Bay | 19.4740 | -155.923 |
| Hawaii | Keaukaha | 19.7320 | -155.047 |
| Hawaii | Kiholo | 19.8571 | -155.929 |
| Hawaii | Kings Landing | 19.7348 | -155.010 |
| Hawaii | Kuhio Kalanianaole Park, Hilo | 19.7256 | -155.062 |
| Hawaii | Laaloa Bay Beach Park | 19.5943 | -155.972 |
| Hawaii | Lalakea Stream (above Waipio Valley) | 20.0754 | -155.688 |
| Hawaii | Laupahoehoe Beach Park | 19.9938 | -155.241 |
| Hawaii | Laupahoehoe Nui | 20.1644 | -155.652 |
| Hawaii | Leleiwi Beach Park | 19.7332 | -155.017 |
| Hawaii | Mahikea Bay, Hilo | 19.7377 | -155.031 |
| Hawaii | Mahukona | 20.1824 | -155.901 |
| Hawaii | Milolii Beach Park | 19.1824 | -155.908 |
| Hawaii | Onekahakaha Beach Park | 19.7381 | -155.039 |
| Hawaii | Pepeekeo | 19.8524 | -155.084 |
| Hawaii | Puako | 19.9658 | -155.854 |
| Hawaii | Puna | 19.5058 | -154.813 |
| Hawaii | Punaluu Black Sand Beach | 19.1359 | -155.505 |
| Hawaii | Queen Liliuokalani Gardens Pond | 19.7267 | -155.069 |
| Hawaii | Reef flat behind the Kona Inn in Kona | 19.6392 | -155.995 |
| Hawaii | Richardson's Beach Park | 19.7352 | -155.013 |
| Hawaii | Shipman Estate/Ranch (Keaau) | 19.6575 | -154.979 |
| Hawaii | South Point Beach (Green Sand Beach) | 18.9161 | -155.669 |
| Hawaii | Spencer's Beach Park | 20.0230 | -155.823 |
| Hawaii | stream along Onomea Scenic drive near Hilo, close to Hawaii Tropical Botanical Garden | 19.8110 | -155.095 |
| Hawaii | Upolu | 20.2669 | -155.859 |
| Hawaii | Wawaloli Beach Park (site 13A) | 19.7137 | -156.049 |
| Hawaii | Wawaloli Beach Park (site 13B) | 19.7218 | -156.057 |
| Hawaii | Whittington Beach Park | 19.0860 | -155.550 |
|  |  |  |  |
| Kahoolawe | Hakioawa | 20.5501 | -156.605 |
| Kahoolawe | Island of Kahoolawe (general) |  |  |
| Kahoolawe | Puukoae | 20.5110 | -156.610 |
|  |  |  |  |
| Kauai | Aliomanu | 22.1722 | -159.314 |
| Kauai | Anahola Beach Park | 22.1507 | -159.306 |
| Kauai | Anini Reef | 22.2234 | -159.438 |
| Kauai | Haena Point | 22.2211 | -159.585 |
| Kauai | Hanalei | 22.2048 | -159.502 |
| Kauai | Hoai Bay | 21.8667 | -159.467 |
| Kauai | Kaluapuhi | 21.9275 | -159.646 |
| Kauai | Kapaa | 22.0733 | -159.316 |
| Kauai | Kealia Lookout | 22.0896 | -159.306 |
| Kauai | Kee Beach | 22.2208 | -159.583 |
| Kauai | Kekaha | 21.9639 | -159.711 |
| Kauai | Kipukai | 21.9105 | -159.390 |
| Kauai | Kokoa Pt. | 21.8778 | -159.467 |
| Kauai | Kokoa Pt., tidepools | 21.8778 | -159.467 |
| Kauai | Lawai | 21.8819 | -159.477 |
| Kauai | Limahuli | 22.2236 | -159.574 |
| Kauai | Lydgate State Park | 22.0410 | -159.335 |
| Kauai | Milolii | 22.1469 | -159.727 |
| Kauai | Moloaa | 22.1935 | -159.333 |
| Kauai | Nawiliwili Harbor | 21.9502 | -159.358 |
| Kauai | Nawiliwili Park | 21.9588 | -159.352 |
| Kauai | Nualolo Kai | 22.1597 | -159.702 |
| Kauai | Papaa Bay | 22.1706 | -159.311 |
| Kauai | Poipu (by Sheraton Kauai Resort) | 21.8762 | -159.464 |
| Kauai | Poipu Beach | 21.8751 | -159.461 |
| Kauai | Poipu Beach Park | 21.8728 | -159.452 |
| Kauai | Polihale Beach Park | 22.1011 | -159.744 |
| Kauai | Port Allen | 21.9009 | -159.588 |
| Kauai | Salt Pond Park | 21.8981 | -159.608 |
| Kauai | South Shore Reef | 21.9614 | -159.707 |
| Kauai | Spouting Horn Beach Park | 21.8848 | -159.495 |
| Kauai | Tunnels Beach | 22.2213 | -159.567 |
| Kauai | unnamed beach (near Wainiha Beach Park) | 22.2182 | -159.542 |
| Kauai | Wainiha Bay | 22.2195 | -159.544 |
| Kauai | Island of Kauai (general) |  |  |
|  |  |  |  |
| Kure Atoll | Kure Atoll (general) | 28.4125 | -178.324 |
|  |  |  |  |
| Lanai | Hulopoe Bay | 20.7390 | -156.894 |
| Lanai | Hulopoe Beach Park | 20.7368 | -156.893 |
| Lanai | Kaiolohia Bay | 20.9201 | -156.909 |
| Lanai | Kapihaa Bay | 20.7361 | -156.906 |
| Lanai | Kaumalapau Harbor | 20.7849 | -156.993 |
| Lanai | Kaumalapau Harbor | 20.7869 | -156.990 |
| Lanai | Manele Bay | 20.7416 | -156.888 |
| Lanai | Naha | 20.7659 | -156.831 |
| Lanai | near Lopa (west end of Lanai) | 20.8038 | -156.808 |
| Lanai | north end of Keomuku Rd. (southern Shipwreck beach) | 20.9145 | -156.900 |
| Lanai | North of Lopa, east side of island | 20.8086 | -156.806 |
| Lanai | outside of Kaumalapau Harbor | 20.7875 | -156.991 |
| Lanai | Polihua Beach | 20.9172 | -157.044 |
|  |  |  |  |
| Laysan | Laysan Island (general) | 25.7673 | -171.733 |
|  |  |  |  |
| Lisianski | Lisianski Island (general) | 26.0585 | -173.929 |
|  |  |  |  |
| Maro | Maro Reef (general) | 25.3670 | -170.624 |
|  |  |  |  |
| Maui | Ahihi Bay | 20.6268 | -156.444 |
| Maui | D.T.Fleming Beach Park | 21.0019 | -156.666 |
| Maui | East Maui Irrigation ditch by Hana Highway | 20.8175 | -156.133 |
| Maui | Hamoa Beach | 20.7201 | -155.987 |
| Maui | Hana Bay | 20.7589 | -155.984 |
| Maui | Hana Beach Park | 20.7564 | -155.985 |
| Maui | Hana Boat Harbor | 20.7557 | -155.982 |
| Maui | Hekili Point | 20.8108 | -156.621 |
| Maui | Honokowai | 20.9547 | -156.689 |
| Maui | Honolua Bay | 21.0142 | -156.638 |
| Maui | Honomanu Bay | 20.8606 | -156.166 |
| Maui | Hookeana Bay | 20.9978 | -156.670 |
| Maui | Hookipa Park | 20.9336 | -156.357 |
| Maui | Kaanapali Beach | 20.9228 | -156.696 |
| Maui | Kahana | 20.9758 | -156.680 |
| Maui | Kahekili Park | 20.9368 | -156.693 |
| Maui | Kahului Bay | 20.8942 | -156.477 |
| Maui | Kahului Harbor Park | 20.8969 | -156.478 |
| Maui | Kaihalulu Beach | 20.7526 | -155.982 |
| Maui | Kalama Park in Kihei | 20.7321 | -156.455 |
| Maui | Kalepolepo Fish Pond | 20.7651 | -156.459 |
| Maui | Kanaha Beach Park | 20.9011 | -156.441 |
| Maui | Kanaha Pond | 20.8921 | -156.458 |
| Maui | Kapalua Bay | 20.9996 | -156.667 |
| Maui | Kawililipoa | 20.7471 | -156.459 |
| Maui | Keanae | 20.8653 | -156.145 |
| Maui | Kihei | 20.7761 | -156.463 |
| Maui | Kihei (Lipoa Street beach entry) | 20.7474 | -156.458 |
| Maui | La Perouse Bay | 20.5996 | -156.420 |
| Maui | Lahaina | 20.8765 | -156.682 |
| Maui | Launiupoko | 20.8429 | -156.654 |
| Maui | Launiupoko Wayside Park | 20.8419 | -156.652 |
| Maui | Maalaea | 20.7913 | -156.511 |
| Maui | Maalaea (by Maalaea Banyan Condominium) | 20.7950 | -156.506 |
| Maui | Makena (golf course) | 20.6443 | -156.446 |
| Maui | Makena, Black Sand Beach | 20.6310 | -156.449 |
| Maui | Mala Wharf Boat Ramp | 20.8857 | -156.687 |
| Maui | Maluaka Beach (Maui Prince Hotel) | 20.6483 | -156.443 |
| Maui | Napili Bay | 20.9955 | -156.667 |
| Maui | near Maalaea Boat Harbor | 20.7948 | -156.507 |
| Maui | Olowalu | 20.8088 | -156.622 |
| Maui | Oneuli (Black Sand Beach) | 20.6439 | -156.449 |
| Maui | Paia | 20.9177 | -156.382 |
| Maui | Papaula | 20.9115 | -156.422 |
| Maui | Polo Beach | 20.6757 | -156.444 |
| Maui | Puaakaa State Wayside (ditch) | 20.8172 | -156.125 |
| Maui | Puamana | 20.8587 | -156.669 |
| Maui | Puunoa (Lahaina) | 20.8820 | -156.688 |
| Maui | Rock jetty in Kahului Harbor, behind the Maui Seaside Hotel | 20.8919 | -156.470 |
| Maui | Wahikuli Park (Lahaina) | 20.8950 | -156.685 |
| Maui | Waiehu Beach Park | 20.9324 | -156.498 |
| Maui | Waihee Beach | 20.9367 | -156.507 |
| Maui | Wailea Beach | 20.6888 | -156.446 |
| Maui | Waioka (Venus Pool) | 20.7043 | -155.995 |
| Maui | Island of Maui (general) |  |  |
|  |  |  |  |
| Midway | Midway Island (general) | 28.2286 | -177.371 |
| Midway | Sand Island | 28.2077 | -177.376 |
|  |  |  |  |
| Molokai | Halawa | 21.1583 | -156.737 |
| Molokai | Hale O Lono Harbor | 21.0871 | -157.250 |
| Molokai | Halena | 21.0859 | -157.249 |
| Molokai | Honolui Maloo Bay Beach | 21.1148 | -156.738 |
| Molokai | Honouli | 21.1153 | -156.737 |
| Molokai | Hotel Molokai | 21.0786 | -156.997 |
| Molokai | Ilio Point | 21.2210 | -157.259 |
| Molokai | Kakahaia Beach Park | 21.0626 | -156.940 |
| Molokai | Kaluakoi (Golf course) | 21.1876 | -157.248 |
| Molokai | Kamalo Wharf | 21.0468 | -156.875 |
| Molokai | Kamiloloa | 21.0780 | -157.002 |
| Molokai | Kamolo | 21.0841 | -157.021 |
| Molokai | Kanalukaha Beach | 21.0884 | -157.255 |
| Molokai | Kapukahehu Beach | 21.1405 | -157.291 |
| Molokai | Kaunakakai | 21.0838 | -157.024 |
| Molokai | Keawanui Fishpond | 21.0578 | -156.853 |
| Molokai | Murphy's Beach | 21.1060 | -156.746 |
| Molokai | One Alii Park II | 21.0708 | -156.977 |
| Molokai | Pakaa Beach | 21.1490 | -157.285 |
| Molokai | Palaau | 21.1798 | -157.013 |
| Molokai | Poolau Beach | 21.1545 | -157.279 |
| Molokai | Pukoo | 21.0661 | -156.805 |
| Molokai | Ualapue Fishpond | 21.0584 | -156.833 |
| Molokai | Wavecrest condominiums | 21.0538 | -156.841 |
| Molokai | Island of Molokai (general) |  |  |
|  |  |  |  |
| Necker | Island of Necker (general) | 23.5747 | -164.702 |
|  |  |  |  |
| Nihoa | Island of Nihoa (general) | 23.0586 | -161.905 |
|  |  |  |  |
| Niihau | Nanina Beach | 22.0019 | -160.087 |
|  |  |  |  |
| NWHI | Northwestern Hawaiian Islands unspecified location |  |  |
|  |  |  |  |
| Oahu | "IZ" Beach | 21.5978 | -157.896 |
| Oahu | Ala Moana Beach Park | 21.2883 | -157.852 |
| Oahu | Ala Moana Beach Park (Kewalo side) | 21.2908 | -157.856 |
| Oahu | Anuenue Fisheries Center (Hawaii Division of Aquatic Resources) | 21.3065 | -157.874 |
| Oahu | b/w Pokai Bay Beach Park & Lualualei Beach Park | 21.4407 | -158.190 |
| Oahu | Barber's Point | 21.2952 | -158.109 |
| Oahu | BOT480 Collection Tank (University of Hawaii) | 21.3016 | -157.815 |
| Oahu | Castle Beach | 21.7041 | -157.959 |
| Oahu | Castle Point (Kailua Bay) | 21.4252 | -157.740 |
| Oahu | Cockroach Bay near Makapuu | 21.3180 | -157.665 |
| Oahu | Coconut Island (Moku o Loe) | 21.4338 | -157.788 |
| Oahu | Cromwell's Beach | 21.2562 | -157.800 |
| Oahu | Diamond Head | 21.2554 | -157.804 |
| Oahu | Ewa Beach | 21.3103 | -158.006 |
| Oahu | Fort Kamehameha | 21.3211 | -157.961 |
| Oahu | Gray's Beach (Waikiki) | 21.2783 | -157.835 |
| Oahu | Haleiwa | 21.5934 | -158.110 |
| Oahu | Halona Beach Cove (Cockroach Cove) | 21.2817 | -157.677 |
| Oahu | Hanauma Bay | 21.2704 | -157.695 |
| Oahu | Hauula Beach Park | 21.6117 | -157.910 |
| Oahu | Heeia | 21.4310 | -157.806 |
| Oahu | Hunakai | 21.2569 | -157.798 |
| Oahu | Ka ala wai | 21.2563 | -157.800 |
| Oahu | Kaaawa (roadside) | 21.5499 | -157.846 |
| Oahu | Kaaawa Beach Park | 21.5576 | -157.856 |
| Oahu | Kahala Beach Park | 21.2692 | -157.777 |
| Oahu | Kahaluu Beach Park | 21.4602 | -157.840 |
| Oahu | Kahana Bay | 21.5584 | -157.876 |
| Oahu | Kahanahaiki | 21.5683 | -158.269 |
| Oahu | Kahe Point (Electric Beach) | 21.3545 | -158.131 |
| Oahu | Kahuku | 21.7105 | -157.983 |
| Oahu | Kaiaka Beach Park (near Haleiwa) | 21.5839 | -158.124 |
| Oahu | Kailua | 21.3975 | -157.724 |
| Oahu | Kailua Beach | 21.3973 | -157.725 |
| Oahu | Kaimana Beach | 21.2636 | -157.822 |
| Oahu | Kalaeloa | 21.2957 | -158.082 |
| Oahu | Kalawahine hiking trail near summit of Round Top Drive, Honolulu | 21.3352 | -157.817 |
| Oahu | Kaloko | 21.2923 | -157.659 |
| Oahu | Kaneohe Bay | 21.4577 | -157.820 |
| Oahu | Kapapa Island (Kaneohe Bay) | 21.4654 | -157.777 |
| Oahu | Kawaihoa | 21.2614 | -157.711 |
| Oahu | Kawaikui Beach Park | 21.2787 | -157.744 |
| Oahu | Kawailoa | 21.6249 | -158.078 |
| Oahu | Kewalo Basin Beach Park | 21.2908 | -157.858 |
| Oahu | Kewalo Marine Laboratory | 21.2908 | -157.861 |
| Oahu | Ko Olina | 21.3315 | -158.124 |
| Oahu | Kualoa Beach Park | 21.5124 | -157.835 |
| Oahu | Kualoa, across from old Sugar Mill | 21.5224 | -157.835 |
| Oahu | Kukaimanini Island | 21.6964 | -158.024 |
| Oahu | Laie | 21.6463 | -157.918 |
| Oahu | Lanai Lookout | 21.2784 | -157.681 |
| Oahu | Lanikai | 21.3911 | -157.714 |
| Oahu | Lualualei | 21.3857 | -158.151 |
| Oahu | Magic Island | 21.2847 | -157.849 |
| Oahu | Maili Point | 21.4054 | -158.179 |
| Oahu | Makaha Beach Park | 21.4758 | -158.221 |
| Oahu | Makai Pier near Makapuu | 21.3183 | -157.669 |
| Oahu | Makapuu | 21.3101 | -157.656 |
| Oahu | Makua Beach | 21.5294 | -158.230 |
| Oahu | Malaekahana Beach Park | 21.6687 | -157.936 |
| Oahu | Manana Island (Rabbit Island) | 21.3280 | -157.657 |
| Oahu | Manoa Stream | 21.2961 | -157.814 |
| Oahu | Maunalua Bay | 21.2811 | -157.719 |
| Oahu | Moanalua Valley | 21.3814 | -157.853 |
| Oahu | Mokuleia | 21.5817 | -158.154 |
| Oahu | Nanakuli | 21.3784 | -158.145 |
| Oahu | near Honolulu Airport | 21.2973 | -157.931 |
| Oahu | near Ulehawa Beach Park (by Mohihi St.) | 21.3915 | -158.157 |
| Oahu | Nimitz Beach (Barber's Point NAS) | 21.2976 | -158.074 |
| Oahu | North Shore (unspecified location) |  |  |
| Oahu | Nuuanu Pali Lookout (along Old Pali Highway hiking trail); wet wall | 21.3670 | -157.792 |
| Oahu | Off Ala Moana | 21.2856 | -157.868 |
| Oahu | Pahole | 21.5859 | -158.173 |
| Oahu | Paiko | 21.2808 | -157.724 |
| Oahu | Pokai Bay | 21.4430 | -158.190 |
| Oahu | Popoia Island (Flat Island) | 21.3996 | -157.720 |
| Oahu | Portlock | 21.2738 | -157.709 |
| Oahu | Punaluu | 21.5969 | -157.895 |
| Oahu | Pupukea, Shark's Cove | 21.6514 | -158.062 |
| Oahu | Puu Noenoe | 21.6322 | -158.074 |
| Oahu | Quarry Pond Stream, Manoa | 21.2955 | -157.815 |
| Oahu | reef in front of Kahala Mandarin Hotel | 21.2710 | -157.773 |
| Oahu | Rock bench at Portlock, Hawaii Kai | 21.2662 | -157.711 |
| Oahu | Sand Island (Mokauea) | 21.3081 | -157.892 |
| Oahu | Sandy Beach | 21.2860 | -157.669 |
| Oahu | southern Diamond Head (close to Beach Rd.) | 21.2552 | -157.809 |
| Oahu | Swanzy Beach Park (Kaaawa) | 21.5576 | -157.856 |
| Oahu | Three Tables break, north shore | 21.6456 | -158.064 |
| Oahu | Ulehawa Beach Park | 21.4017 | -158.178 |
| Oahu | Waianae Boat Harbor | 21.4483 | -158.197 |
| Oahu | Waianae coast | 21.4434 | -158.191 |
| Oahu | Waianae Regional Park | 21.4496 | -158.197 |
| Oahu | Waihee Stream | 21.4681 | -157.849 |
| Oahu | Waikiki | 21.2723 | -157.826 |
| Oahu | Waikiki MLCD Trench | 21.2655 | -157.823 |
| Oahu | Waikiki Natatorium | 21.2643 | -157.823 |
| Oahu | Wailupe | 21.2735 | -157.756 |
| Oahu | Waimanalo | 21.3326 | -157.693 |
| Oahu | Waimea Bay | 21.6385 | -158.066 |
| Oahu | West Beach, Ewa Beach | 21.3109 | -158.000 |
| Oahu | Whitehouse | 21.6343 | -158.070 |
| Oahu | Windward side (unspecified location) |  |  |
| Oahu | Yokohama Bay | 21.5553 | -158.249 |
| Oahu | Island of Oahu (general) |  |  |
|  |  |  |  |
| Pearl and Hermes Atoll | Pearl and Hermes Atoll (general) | 27.8333 | -175.833 |
