# Supplementary material for: The Hawaiian Rhodophyta Biodiversity Survey (2006-2010): a summary of principal findings
Source: BMC Plant Biol. 2010 Nov 22;10:258. doi: 10.1186/1471-2229-10-258 (PMC3012605; doi:10.1186/1471-2229-10-258)
Supplement: Additional file 2 — Taxonomic checklist and new records. Taxonomic checklist and new records from the Hawaiian Rhodophyta Biodiversity Project. Taxon records are arranged by order. New taxon and island records are indicated in bold. [file 1471-2229-10-258-S2.DOCX]

**Additional file 2.** Taxonomic checklist and new records.

| **Order** | **Family** | **Genus** | **Species** | **Authority** | **Island(s) of distribution** |
| --- | --- | --- | --- | --- | --- |
| Acrosymphytales | Acrosymphytaceae | *Acrosymphyton* | sp. |  | Oahu |
| Acrosymphytales | Acrosymphytaceae | *Acrosymphyton* | *taylorii* | Abbott | Oahu |
| Bangiales | Bangiaceae | *Bangia* | *fuscopurpurea* | (Dillwyn) Lyngbye | Maui |
| Bangiales | Bangiaceae | *Porphyra* | sp. |  | Hawaii, Kauai, **Maui**, Oahu |
| Bangiales | Bangiaceae | *Porphyra* | *vietnamensis* | Tanaka et Pham | Oahu |
| Bangiales | Bangiaceae | ***Pseudobangia*** | **sp.** |  | **Oahu** |
| Batrachospermales | Batrachospermaceae | *Batrachospermum* | sp. |  | Hawaii, Oahu |
| Batrachospermales | Batrachospermaceae | *Chantransia* | sp.  (of *Batrachospermum arcuatum*) |  | Hawaii, Oahu |
| Batrachospermales | Batrachospermaceae | *Kumanoa* | *spermatiophora* | (M.L. Vis et Sheath) Entwisle, M.L. Vis, W.B. Chiasson, Necchi et Sherwood | Maui |
| Bonnemaisoniales | Bonnemaisoniaceae | *Asparagopsis* | *taxiformis* | (Delile) Trevisan | French Frigate Shoals, Hawaii, **Kahoolawe**, Kauai, Kure, Lanai, Laysan, Lisianski, Maui, Midway, Molokai, Necker, Nihoa, Oahu |
| Bonnemaisoniales | Naccariaceae | *Naccaria* | *hawaiiana* | Abbott | Oahu |
| Bonnemaisoniales | Naccariaceae | *Reticulocaulis* | *mucosissimus* | Abbott | Oahu |
| Ceramiales | Callithamniaceae | *Aglaothamnion* | *boergesenii* | (Aponte et Ballantine) L'Hardy-Halos et Rueness | **Maui**, Oahu |
| Ceramiales | Callithamniaceae | *Aglaothamnion* | *cordatum* | (Børgesen) Feldmann- Mazoyer | Maui |
| Ceramiales | Callithamniaceae | *Aglaothamnion* | sp. |  | **Hawaii**, Molokai, Oahu |
| Ceramiales | Callithamniaceae | *Callithamnion* | sp. |  | Kure Atoll |
| Ceramiales | Callithamniaceae | *Crouania* | *minutissima* | Yamada | **Kure Atoll** |
| Ceramiales | Callithamniaceae | *Crouania* | sp. |  | **Hawaii**, **Kure**, **Molokai**, Oahu |
| Ceramiales | Callithamniaceae | *Euptilocladia* | *magruderi* | Abbott et R.E. Norris | **Kauai**, **Lanai**, **Molokai**, Oahu |
| Ceramiales | Callithamniaceae | *Gymnothamnion* | *elegans* | (Schousboe ex C. Agardh) J. Agardh | **Lanai**, Oahu |
| Ceramiales | Ceramiaceae | *Acrothamnion* | *butleriae* | (Collins) Kylin | Oahu |
| Ceramiales | Ceramiaceae | *Antithamnion* | *antillanum* | Børgesen | **Maro**, Maui, **Molokai**, Oahu |
| Ceramiales | Ceramiaceae | *Antithamnion* | *decipiens* | (J. Agardh) Athanasiadis | HURL CRUISE |
| Ceramiales | Ceramiaceae | *Antithamnion* | *erucacladellum* | R.E. Norris | **Lanai** |
| Ceramiales | Ceramiaceae | *Antithamnion* | sp. |  | HURL CRUISE, Oahu |
| Ceramiales | Ceramiaceae | *Balliella* | *repens* | Huisman et Kraft | **Lanai** |
| Ceramiales | Ceramiaceae | *Centroceras* | *clavulatum* | (C. Agardh) Montagne | French Frigate Shoals, Hawaii, **Lanai**, Oahu |
| Ceramiales | Ceramiaceae | *Centroceras* | sp. |  | Hawaii, Kauai, Oahu |
| Ceramiales | Ceramiaceae | *Ceramium* | *codii* | (Richards) G. Mazoyer | **Kure Atoll** |
| Ceramiales | Ceramiaceae | *Ceramium* | *dumosertum* | R.E. Norris et Abbott | Hawaii, **Kure Atoll**, Maui, Oahu |
| Ceramiales | Ceramiaceae | *Ceramium* | *hanaense* | R.E. Norris et Abbott | **Kure Atoll** |
| Ceramiales | Ceramiaceae | *Ceramium* | *hyalacanthus* | (Kützing) Sonder | Oahu |
| Ceramiales | Ceramiaceae | *Ceramium* | sp*.* |  | Hawaii, HURL CRUISE, Kauai, Kure, Lanai, Maro, Maui, Midway, Molokai, Oahu, Pearl & Hermes |
| Ceramiales | Ceramiaceae | *Ceramium* | *womersleyi* | R.E. Norris et Abbott | Maui |
| Ceramiales | Ceramiaceae | *Corallophila* | *huysmansii* | (Weber-van Bosse) R.E. Norris | **Kure Atoll** |
| Ceramiales | Ceramiaceae | *Diplothamnion* | *jolyi* | van den Hoek | HURL CRUISE, **Lanai**, Oahu |
| Ceramiales | Ceramiaceae | *Gayliella* | *fimbriata* | (Setchell et Gardner) T.O. Cho et S.M. Boo | Oahu |
| Ceramiales | Ceramiaceae | *Gayliella* | sp. |  | Oahu, Lanai, Molokai |
| Ceramiales | Ceramiaceae | *Perikladosporon* | *percurrens* | (Dawson) Athanasiadis | Hawaii, **Lanai** |
| Ceramiales | Dasyaceae | *Dasya* | *anastomosans* | (Weber-van Bosse) M.J. Wynne | Hawaii, **Necker**, NWHI |
| Ceramiales | Dasyaceae | *Dasya* | *atropurpurea* | Vroom | **French Frigate Shoals**, **Maro**, Necker |
| Ceramiales | Dasyaceae | *Dasya* | *corymbifera* | J. Agardh | Oahu |
| Ceramiales | Dasyaceae | *Dasya* | *iridescens* | (Schlech) Millar et Abbott | French Frigate Shoals, Kauai, **Lisianski**, **Maro**, Maui, Molokai, Necker, NWHI, Oahu |
| Ceramiales | Dasyaceae | *Dasya* | *kristeniae* | Abbott | French Frigate Shoals, Oahu |
| Ceramiales | Dasyaceae | *Dasya* | *murrayana* | Abbott et Millar | Kauai, Oahu |
| Ceramiales | Dasyaceae | *Dasya* | sp. |  | Hawaii, Kauai, Maro, Maui, Midway, Molokai, Necker, NWHI, Oahu |
| Ceramiales | Dasyaceae | *Heterosiphonia* | *crispella* | (C. Agardh) M.J. Wynne | Hawaii, HURL CRUISE, **Molokai**, Oahu |
| Ceramiales | Dasyaceae | *Heterosiphonia* | sp. |  | Oahu |
| Ceramiales | Delesseriaceae | *Cryptopleura* | *peltata* | (Montagne) Wynne | Maui |
| Ceramiales | Delesseriaceae | *Hypoglossum* | *caloglossoides* | Wynne et Kraft | **Kure Atoll** |
| Ceramiales | Delesseriaceae | *Hypoglossum* | *rhizophorum* | Ballantine et Wynne | Maui |
| Ceramiales | Delesseriaceae | *Hypoglossum* | sp. |  | Maui |
| Ceramiales | Delesseriaceae | *Martensia* | *flabelliformis* | Harvey ex J. Agardh | French Frigate Shoals, Hawaii, Maui, **Molokai**, Oahu |
| Ceramiales | Delesseriaceae | *Martensia* | *fragilis* | Harvey | French Frigate Shoals, Hawaii, Kauai, Maui, Oahu |
| Ceramiales | Delesseriaceae | *Martensia* | sp. |  | Hawaii, Kauai, **Lanai**, Molokai, **Niihau**, Oahu |
| Ceramiales | Delesseriaceae | *Nitophyllum* | *adhaerens* | Wynne | **Kure Atoll**, Maui, **Molokai**, Oahu |
| Ceramiales | Delesseriaceae | *Taenioma* | *perpusillum* | (J. Agardh) J. Agardh | Hawaii, Midway, Molokai, Oahu |
| Ceramiales | Delesseriaceae | *Taenioma* | sp. |  | Oahu |
| Ceramiales | Delesseriaceae | *Vanvoorstia* | *coccinea* | J. Agardh | Hawaii, **Maro** |
| Ceramiales | Rhodomelaceae | *Acanthophora* | *pacifica* | (Setchell) Kraft | Hawaii, HURL CRUISE, Kahoolawe, Maui, **Molokai**, Oahu |
| Ceramiales | Rhodomelaceae | *Acanthophora* | *spicifera* | (Vahl) Børgesen | Hawaii, Kauai, Molokai, **Niihau**, Oahu |
| Ceramiales | Rhodomelaceae | *Alsidium* | *cymatophilum* | R.E. Norris | **Molokai**, Oahu |
| Ceramiales | Rhodomelaceae | *Amansia* | *fimbrifolia* | (R.E. Norris) L.E. Phillips | **Hawaii**, **Laysan**, **Maro**, **Maui**, **Molokai**, Oahu, Pearl and Hermes |
| Ceramiales | Rhodomelaceae | *Amansia* | *glomerata* | C. Agardh | French Frigate Shoals, Hawaii, Kauai, **Lanai**, Laysan, Maui, **Molokai**, **Necker**, Nihoa, **Niihau**, Oahu, **Pearl and Hermes** |
| Ceramiales | Rhodomelaceae | *Amansia* | sp. |  | Hawaii, HURL CRUISE, Kauai, Maui, Molokai, Oahu |
| Ceramiales | Rhodomelaceae | *Chondria* | *arcuata* | Hollenberg | **Molokai** |
| Ceramiales | Rhodomelaceae | *Chondria* | *dangeardii* | Dawson | Maui, Molokai |
| Ceramiales | Rhodomelaceae | *Chondria* | *simpliciuscula* | Weber-van Bosse | French Frigate Shoals, Maui |
| Ceramiales | Rhodomelaceae | *Chondria* | sp. |  | HURL CRUISE, Kauai, Molokai, Oahu |
| Ceramiales | Rhodomelaceae | *Chondrophycus* | cf. *undulatus* | (Yamada) Garbary et Harper | Kauai, Maui, Oahu |
| Ceramiales | Rhodomelaceae | *Chondrophycus* | *dotyi* | (Saito) K.W. Nam | **French Frigate Shoals**, Kauai, Oahu |
| Ceramiales | Rhodomelaceae | *Chondrophycus* | *glanduliferus* | (Kützing) Lipkin & Silva | Maui |
| Ceramiales | Rhodomelaceae | *Chondrophycus* | sp. |  | Hawaii, Kauai, Lanai, Maui, Molokai, Oahu |
| Ceramiales | Rhodomelaceae | *Chondrophycus* | *succisus* | (Cribb) Nam | Hawaii, Kauai, Maui, Molokai |
| Ceramiales | Rhodomelaceae | *Exophyllum* | *wentii* | Weber-van Bosse | Hawaii |
| Ceramiales | Rhodomelaceae | *Herposiphonia* | sp. |  | Hawaii, HURL CRUISE, **Lanai**, Midway, Molokai, Oahu, **Pearl and Hermes** |
| Ceramiales | Rhodomelaceae | *Janczewskia* | *hawaiiana* | Apt | Oahu |
| Ceramiales | Rhodomelaceae | *Laurencia* | *brachyclados* | Pilger | French Frigate Shoals, **Kauai**, **Lanai**, **Necker** |
| Ceramiales | Rhodomelaceae | *Laurencia* | *crustiformans* | McDermid | Hawaii, Kauai, Maui, Oahu |
| Ceramiales | Rhodomelaceae | *Laurencia* | *decumbens* | Kützing | Kauai, **Molokai**, Oahu |
| Ceramiales | Rhodomelaceae | *Laurencia* | *galtsoffii* | Howe | **Lanai**, Midway, Necker, Oahu |
| Ceramiales | Rhodomelaceae | *Laurencia* | *majuscula* | (Harvey) Lucas | French Frigate Shoals, Kauai, **Lanai**, Laysan, Molokai, Oahu |
| Ceramiales | Rhodomelaceae | *Laurencia* | *mariannensis* | Yamada | Hawaii, Oahu |
| Ceramiales | Rhodomelaceae | *Laurencia* | *mcdermidiae* | Abbott | **Hawaii**, **Kauai**, Maui, **Molokai**, Oahu |
| Ceramiales | Rhodomelaceae | *Laurencia* | *nidifica* | J. Agardh | **Hawaii**, Maui, Molokai, Oahu |
| Ceramiales | Rhodomelaceae | *Laurencia* | sp. |  | French Frigate Shoals, Hawaii, Kauai, Kure, Lanai, Maro, Maui, Molokai, Oahu |
| Ceramiales | Rhodomelaceae | *Laurencia* | *tenera* | C.K. Tseng | Oahu |
| Ceramiales | Rhodomelaceae | *Leveillea* | *jungermannioides* | (Hering et Martens) Harvey | Maui, Oahu |
| Ceramiales | Rhodomelaceae | *Lophocladia* | *kipukaia* | Schlech | Kauai |
| Ceramiales | Rhodomelaceae | *Lophocladia* | sp. |  | HURL CRUISE, Oahu |
| Ceramiales | Rhodomelaceae | *Osmundaria* | *obtusiloba* | (C. Agardh) R.E. Norris | Maui, Oahu |
| Ceramiales | Rhodomelaceae | *Palisada* | *cartilaginea* | (Yamada) Nam | **French Frigate Shoals**, Oahu |
| Ceramiales | Rhodomelaceae | *Palisada* | cf. *cartilaginea* | (Yamada) Garbary & Harper | **Kauai**, Maui, **Molokai**, Oahu |
| Ceramiales | Rhodomelaceae | *Palisada* | *parvipapillata* | (C.K. Tseng) Nam | French Frigate Shoals, Hawaii, Kauai, **Lanai**, Maui, Molokai, Oahu |
| Ceramiales | Rhodomelaceae | *Palisada* | *yamadana* | (Howe) Nam | **Kauai**, **Lanai**, Maui, Oahu |
| Ceramiales | Rhodomelaceae | *Polysiphonia* | *howei* | Hollenberg | Hawaii, **Maui**, Oahu |
| Ceramiales | Rhodomelaceae | *Polysiphonia* | sp. |  | Hawaii, Kure, Lanai, Maui, Midway, Oahu, Pearl and Hermes |
| Ceramiales | Rhodomelaceae | *Polysiphonia* | *tepida* | Hollenberg | **Kure Atoll** |
| Ceramiales | Rhodomelaceae | *Polysiphonia* | *upolensis* | (Grunow) Hollenberg | Kure Atoll |
| Ceramiales | Rhodomelaceae | *Pterosiphonia* | *pennata* | (C. Agardh) Falkenberg | **Maui** |
| Ceramiales | Rhodomelaceae | *Rhodolachne* | *decussata* | Wynne | **Hawaii** |
| Ceramiales | Rhodomelaceae | *Spirocladia* | *barodensis* | Børgesen | Maui, Molokai |
| Ceramiales | Rhodomelaceae | *Spirocladia* | *hodgsoniae* | Abbott | Maui |
| Ceramiales | Rhodomelaceae | *Tayloriella* | *dictyurus* | (J. Agardh) Kylin | **Hawaii** |
| Ceramiales | Rhodomelaceae | *Tolypiocladia* | *glomerulata* | (C. Agardh) Schmitz | Hawaii, Maui, **Molokai**, Oahu |
| Ceramiales | Rhodomelaceae | *Ululania* | *stellata* | Apt et Schlech | **Hawaii**, **Maui**, Oahu |
| Ceramiales | Sarcomeniaceae | *Dotyella* | *hawaiiensis* | (Doty ex Wainwright) Womersley ex Shepley | Kauai, **Midway**, Oahu |
| Ceramiales | Sarcomeniaceae | *Dotyella* | sp. |  | Oahu |
| Ceramiales | Sarcomeniaceae | *Malaconema* | *minimum* | Hollenberg | HURL CRUISE |
| Ceramiales | Spyridiaceae | *Spyridia* | *filamentosa* | (Wulfen) Harvey | Hawaii, HURL CRUISE, Kahoolawe, Kauai, Kure, **Lanai**, Laysan, **Lisianski**, **Maro**, Maui, Midway, **Molokai**, **Necker**, Oahu |
| Ceramiales | Wrangeliaceae | *Anotrichium* | sp. |  | Hawaii, **Molokai** |
| Ceramiales | Wrangeliaceae | *Anotrichium* | *tenue* | (C. Agardh) Naegeli | Hawaii, Oahu |
| Ceramiales | Wrangeliaceae | *Griffithsia* | *heteromorpha* | Kützing | French Frigate Shoals, Oahu |
| Ceramiales | Wrangeliaceae | *Griffithsia* | *metcalfii* | C.K. Tseng | Oahu |
| Ceramiales | Wrangeliaceae | *Griffithsia* | *schousboei* | Montagne | **Lanai**, Maui, Oahu |
| Ceramiales | Wrangeliaceae | *Griffithsia* | sp. |  | HURL CRUISE, Oahu |
| Ceramiales | Wrangeliaceae | *Griffithsia* | *subcylindrica* | Okamura | Maui, **Molokai**, Oahu |
| Ceramiales | Wrangeliaceae | *Haloplegma* | *duperreyi* | Montagne | French Frigate Shoals, Kauai, **Lanai**, Molokai, Oahu |
| Ceramiales | Wrangeliaceae | *Lejolisia* | *pacifica* | Itono | Maui, Oahu |
| Ceramiales | Wrangeliaceae | *Monosporus* | *indicus* | Børgesen | Oahu |
| Ceramiales | Wrangeliaceae | *Pleonosporium* | sp. |  | **Hawaii**, HURL CRUISE |
| Ceramiales | Wrangeliaceae | *Tiffaniella* | *saccorhiza* | (Setchell et Gardner) Doty et Meñez | HURL CRUISE, Oahu |
| Ceramiales | Wrangeliaceae | *Wrangelia* | *argus* | (Montagne) Montagne | Oahu |
| Ceramiales | Wrangeliaceae | *Wrangelia* | *bicuspidata* | Børgesen | Hawaii |
| Ceramiales | Wrangeliaceae | *Wrangelia* | *elegantissima* | R.E. Norris | Kauai, Oahu |
| Ceramiales | Wrangeliaceae | *Wrangelia* | sp. |  | Kauai, Maui, Oahu |
| Colaconematales | Colaconemataceae | *Colaconema* | sp. |  | Kauai, Maui, Molokai, Oahu |
| Compsopogonales | Compsopogonaceae | *Compsopogon* | *coeruleus* | (Balbis ex C. Agardh) Montagne | Kauai, Maui, Oahu |
| Corallinales | Corallinaceae | *Amphiroa* | *beauvoisii* | Lamouroux | **Hawaii**, Kauai |
| Corallinales | Corallinaceae | *Amphiroa* | *foliacea* | Lamouroux | **Hawaii**, Kauai, **Molokai** |
| Corallinales | Corallinaceae | *Amphiroa* | *rigida* | Lamouroux | **Hawaii**, Maui |
| Corallinales | Corallinaceae | *Amphiroa* | sp. |  | French Frigate Shoals, Kauai, Maui, Oahu |
| Corallinales | Corallinaceae | *Amphiroa* | *valonioides* | Yendo | **Kahoolawe**, Laysan |
| Corallinales | Corallinaceae | *Arthrocardia* | sp. |  | Maui |
| Corallinales | Corallinaceae | cf. *Spongites* |  |  | Oahu |
| Corallinales | Corallinaceae | *Corallina* | sp. |  | Oahu |
| Corallinales | Corallinaceae | *Haliptilon* | *subulatum* | (Ellis et Solander) Johansen | Hawaii, Kauai, Molokai, Oahu |
| Corallinales | Corallinaceae | *Hydrolithon* | *breviclavium* | (Foslie) Foslie | Oahu |
| Corallinales | Corallinaceae | *Hydrolithon* | *gardineri* | (Foslie) Verheij & Prud’homme van Reine | Oahu |
| Corallinales | Corallinaceae | *Hydrolithon* | *reinboldii* | (Weber-van Bosse & Foslie) Foslie | Oahu |
| Corallinales | Corallinaceae | *Hydrolithon* | sp. |  | Kure Atoll, Oahu, Molokai |
| Corallinales | Corallinaceae | *Jania* | *adhaerens* | Lamouroux | French Frigate Shoals, Hawaii |
| Corallinales | Corallinaceae | *Jania* | *micrarthrodia* | Lamouroux | **Hawaii**, **Kauai** |
| Corallinales | Corallinaceae | *Jania* | *pumila* | Lamouroux | **Kauai**, Oahu |
| Corallinales | Corallinaceae | *Jania* | sp. |  | Hawaii, **Molokai**, **Niihau**, Oahu |
| Corallinales | Corallinaceae | *Jania* | *verrucosa* | Lamouroux | **Lanai**, Oahu |
| Corallinales | Corallinaceae | *Lithophyllum* | *insipidum* | Adey, Townsend & Boykins | Hawaii, Lanai, Oahu |
| Corallinales | Corallinaceae | *Lithophyllum* | *kotschyanum* | Unger | Lanai, Molokai, Oahu |
| Corallinales | Corallinaceae | *Mastophora* | *pacifica* | (Heydlich) Foslie | Oahu |
| Corallinales | Corallinaceae | *Metamastophora* | sp. |  | Molokai |
| Corallinales | Corallinaceae | *Neogoniolithon* | *brassica-florida* | (Harvey) Setchell & L.R. Mason | Oahu |
| Corallinales | Corallinaceae | *Pneophyllum* | *conicum* | (E.Y. Dawson) Keats, Y.M. Chamberlain & Baba | Lanai, Lisianaski, Oahu |
| Corallinales | Corallinaceae | *Pneophyllum* | sp. |  | Lanai |
| Corallinales | Corallinaceae | *Titanoderma* | *prototypum* | (Foslie) Woelkerling, Y.M. Chamberlain & Silva | Oahu |
| Corallinales | Hapalidiaceae | *Phymatolithon* | sp. |  | Oahu |
| Corallinales | Hapalidiaceae | *Mesophyllum* | *erubescens* | (Foslie) M. Lemoine | Lanai, Oahu |
| Corallinales | Hapalidiaceae | *Mesophyllum* | sp. |  | Oahu |
| Corallinales | Mastophoroideae | *Mastophora* | sp. |  | Lanai, Molokai, Oahu |
| Erythropeltidales | incertae sedis | ***Madagascaria*** | ***erythrocladioides*** | J.A. West et Kikichi | **Maui** |
| Erythropeltidales | Erythrotrichiaceae | *Erythrocladia* | sp. |  | **Oahu** |
| Erythropeltidales | Erythrotrichiaceae | *Erythrotrichia* | sp. |  | Oahu |
| Erythropeltidales | Erythrotrichiaceae | ***Sahlingia*** | ***subintegra*** | (Rosenvinge) Kornmann | **Hawaii**, **Kauai**, **Maui**, **Oahu** |
| Gelidiales | Gelidiaceae | *Gelidium* | *crinale* | (Turner) Gaillon | Oahu |
| Gelidiales | Gelidiaceae | *Gelidium* | *pluma* | Loomis | Hawaii, Kauai |
| Gelidiales | Gelidiaceae | *Gelidium* | *pusillum* | (Stackhouse) Le Jolis | Hawaii, Kauai, Oahu |
| Gelidiales | Gelidiaceae | *Gelidium* | *reediae* | Loomis | Kauai, **Maui**, **Molokai**, Oahu |
| Gelidiales | Gelidiaceae | *Gelidium* | sp. |  | Hawaii, Kauai, Molokai, Oahu |
| Gelidiales | Gelidiaceae | *Pterocladiella* | *caerulescens* | (Kützing) Santelices et Hommersand | Maui, **Molokai**, **Nihoa**, Oahu |
| Gelidiales | Gelidiaceae | *Pterocladiella* | *caloglossoides* | (Howe) Santelices | **Hawaii** |
| Gelidiales | Gelidiaceae | *Pterocladiella* | *capillacea* | (Gmelin) Santelices et Hommersand | **French Frigate Shoals**, Hawaii, Kauai, Maui |
| Gelidiales | Gelidiaceae | *Pterocladiella* | sp. |  | Kauai, Molokai, Oahu |
| Gelidiales | Gelidiellaceae | *Gelidiella* | *acerosa* | (Forsskål) Feldmann et Hamel | Maui, Oahu |
| Gelidiales | Gelidiellaceae | *Gelidiella* | *machrisiana* | Dawson | Hawaii, **Kauai** |
| Gelidiales | Gelidiellaceae | *Gelidiella* | sp. |  | Maui, Oahu |
| Gigartinales | Calosiphonaceae | *Schmitzia* | sp. |  | Oahu |
| Gigartinales | Caulacanthaceae | *Caulacanthus* | *ustulatus* | (Turner ex Mertens) Kützing | Hawaii |
| Gigartinales | Cystocloniaceae | *Hypnea* | *cervicornis* | J. Agardh | Maui, Oahu |
| Gigartinales | Cystocloniaceae | *Hypnea* | *charoides* | Lamouroux | Necker |
| Gigartinales | Cystocloniaceae | *Hypnea* | *chordacea* | Kützing | **Hawaii**, **Kauai**, Maui, **Molokai**, Oahu |
| Gigartinales | Cystocloniaceae | *Hypnea* | *cornuta* var. *stellulifera* | J. Agardh | Kauai |
| Gigartinales | Cystocloniaceae | *Hypnea* | *musciformis* | (Wulfen in Jacquin) Lamouroux | **Kauai**, Maui, **Necker**, NWHI, Oahu |
| Gigartinales | Cystocloniaceae | *Hypnea* | *nidifica* | J. Agardh | Hawaii, Oahu |
| Gigartinales | Cystocloniaceae | *Hypnea* | *pannosa* | J. Agardh | French Frigate Shoals, Hawaii, **Maro**, Maui, Oahu |
| Gigartinales | Cystocloniaceae | *Hypnea* | sp. |  | Hawaii, Kauai, Maui, Midway, Molokai, **Niihau**, Oahu |
| Gigartinales | Cystocloniaceae | *Hypnea* | *spinella* | (C. Agardh) Kützing | **Kauai**, Oahu |
| Gigartinales | Cystocloniaceae | *Hypnea* | *valentiae* | (Turner) Montagne | Kauai, Maui |
| Gigartinales | Cystocloniaceae | *Hypneocolax* | *stellaris* ssp. *orientalis* | (Weber-van Bosse) Womersley | Oahu |
| Gigartinales | Dumontiaceae | *Dudresnaya* | *hawaiiensis* | R.K.S. Lee | Kauai, Oahu |
| Gigartinales | Dumontiaceae | *Dudresnaya* | *littleri* | Abbott | Oahu |
| Gigartinales | Dumontiaceae | *Dudresnaya* | sp. |  | Oahu |
| Gigartinales | Dumontiaceae | *Gibsmithia* | *dotyi* | Kraft et Ricker | French Frigate Shoals, Oahu |
| Gigartinales | Dumontiaceae | *Gibsmithia* | *hawaiiensis* | Doty | French Frigate Shoals, Hawaii, Kauai, Oahu |
| Gigartinales | Gigartinaceae | *Chondracanthus* | *acicularis* | (Roth) Fredericq | Maui, Oahu |
| Gigartinales | Gigartinaceae | *Chondracanthus* | sp. |  | Hawaii, **Molokai** |
| Gigartinales | Gigartinaceae | *Chondracanthus* | *tenellus* | (Harvey) Hommersand | Oahu |
| Gigartinales | Gigartinaceae | *Chondrus* | *ocellatus* | Holmes | Hawaii, **Maui** |
| Gigartinales | Gigartinaceae | *Mazzaella* | *volans* | (C. Agardh) J. Agardh | Hawaii |
| Gigartinales | Gloiosiphoniaceae | *Peleophycus* | *multiprocarpium* | Abbott | Oahu |
| Gigartinales | Kallymeniaceae | *Kallymenia* | *sessilis* | Okamura | **Kauai**, Oahu |
| Gigartinales | Kallymeniaceae | *Kallymenia* | sp. |  | French Frigate Shoals |
| Gigartinales | Kallymeniaceae | *Kallymenia* | *thompsonii* | Abbott et McDermid | **Hawaii**, **Maro** |
| Gigartinales | Phyllophoraceae | *Ahnfeltiopsis* | *concinna* | (J. Agardh) Silva et DeCew | Hawaii, Kauai, Maui, **Molokai**, Oahu |
| Gigartinales | Phyllophoraceae | *Ahnfeltiopsis* | *divaricata* | (Holmes) Masuda | Kauai, **Maui** |
| Gigartinales | Phyllophoraceae | *Ahnfeltiopsis* | *flabelliformis* | (Harvey) Masuda | Hawaii, Kauai, Maui, **Molokai**, Oahu |
| Gigartinales | Phyllophoraceae | *Ahnfeltiopsis* | *pygmaea* | (J. Agardh) Silva et DeCew | Maui, Oahu |
| Gigartinales | Phyllophoraceae | *Ahnfeltiopsis* | sp. |  | Hawaii, Kauai, Oahu |
| Gigartinales | Rhizophyllidaceae | *Portieria* | *hornemannii* | (Lyngbye) Silva | **French Frigate Shoals**, Hawaii, **Kauai**, Maui, **Molokai**, Oahu |
| Gigartinales | Solieriaceae | *Eucheuma* | *denticulatum* | (Burman) Collins et Hervey | Oahu |
| Gigartinales | Solieriaceae | *Kappaphycus* | *alvarezii* var. *tambalang* | Doty | Oahu |
| Gigartinales | Solieriaceae | *Kappaphycus* | *cottonii* | (Weber-van Bosse) Doty | Oahu |
| Gigartinales | Solieriaceae | *Kappaphycus* | sp. |  | Oahu |
| Gigartinales | Solieriaceae | *Kappaphycus* | *striatum* | (Schmitz) Doty ex Silva | Oahu |
| Gracilariales | Gracilariaceae | *Gracilaria* | *abbottiana* | Hoyle | Kauai, Oahu |
| Gracilariales | Gracilariaceae | *Gracilaria* | *coronopifolia* | J. Agardh | Kauai, Maui, Molokai, Oahu |
| Gracilariales | Gracilariaceae | *Gracilaria* | *dawsonii* | Hoyle | **Kauai**, Oahu |
| Gracilariales | Gracilariaceae | *Gracilaria* | *dotyi* | Hoyle | Kauai, Oahu |
| Gracilariales | Gracilariaceae | *Gracilaria* | *epihippisora* | Hoyle | Hawaii |
| Gracilariales | Gracilariaceae | *Gracilaria* | *parvispora* | Abbott | Molokai, Oahu |
| Gracilariales | Gracilariaceae | *Gracilaria* | *salicornia* | (C. Agardh) Dawson | Hawaii, Molokai, Oahu |
| Gracilariales | Gracilariaceae | *Gracilaria* | sp. |  | Hawaii, Kauai, Maui, Molokai, Oahu |
| Gracilariales | Gracilariaceae | *Gracilaria* | *tikvahiae* | McLachlan | **Hawaii**, Oahu |
| Gracilariales | Gracilariaceae | *Gracilariopsis* | *lemaneiformis* | (Bory) Dawson, Acleto et Foldvik | Molokai, Oahu |
| Halymeniales | Halymeniaceae | *Cryptonemia* | *umbraticola* | Dawson | **Hawaii** |
| Halymeniales | Halymeniaceae | *Cryptonemia* | *yendoi* | Weber-van Bosse | **Kauai**, **Lanai**, Oahu |
| Halymeniales | Halymeniaceae | *Grateloupia* | cf. *filicina* |  | Maui, Oahu |
| Halymeniales | Halymeniaceae | *Grateloupia* | *filicina* | (Lamouroux) C. Agardh | Oahu, Maui, Molokai, Hawaii |
| Halymeniales | Halymeniaceae | *Grateloupia* | *hawaiiana* | Dawson | Hawaii, Maui, Oahu |
| Halymeniales | Halymeniaceae | *Grateloupia* | *phuquocensis* | Tanaka et Pham | Hawaii, **Kauai**, Maui, Oahu |
| Halymeniales | Halymeniaceae | *Grateloupia* | sp. |  | Oahu |
| Halymeniales | Halymeniaceae | *Halymenia* | *actinophysa* | Howe | **Oahu** |
| Halymeniales | Halymeniaceae | *Halymenia* | *chiangiana* | Abbott et Kraft | Maui, **Oahu** |
| Halymeniales | Halymeniaceae | *Halymenia* | *formosa* | Harvey ex Kützing | **Hawaii**, Oahu |
| Halymeniales | Halymeniaceae | *Halymenia* | sp. |  | Maui, Oahu |
| Halymeniales | Halymeniaceae | *Halymenia* | *stipitata* | Abbott | Maui, Oahu |
| Halymeniales | Halymeniaceae | *Polyopes* | *hakalauensis* | (Tilden) Abbott | Hawaii, Maui |
| Halymeniales | Halymeniaceae | *Prionitis* | *corymbifera* | Abbott | Hawaii |
| Hildenbrandiales | Hildenbrandiaceae | *Hildenbrandia* | *angolensis* | Welwitsch ex W. West et G.S. West | Oahu |
| Hildenbrandiales | Hildenbrandiaceae | *Hildenbrandia* | *rubra* | (Sommerfelt) Meneghini | **Maui**, Oahu |
| Nemaliales | Galaxauraceae | *Actinotrichia* | *fragilis* | (Forsskål) Børgesen | Kauai, **Niihau**, Oahu |
| Nemaliales | Galaxauraceae | *Dichotomaria* | cf. *marginata* |  | Hawaii, Kauai, Maui, Molokai, Oahu |
| Nemaliales | Galaxauraceae | *Dichotomaria* | *marginata* | (Ellis et Solander) Lamarck | Hawaii, **Kauai**, **Lanai**, Maui, **Molokai**, Oahu |
| Nemaliales | Galaxauraceae | *Galaxaura* | *apiculata* | Kjellman | Hawaii |
| Nemaliales | Galaxauraceae | *Galaxaura* | *divaricata* | (Linnaeus) Huisman & Townsend | **Lanai**, **Maui** |
| Nemaliales | Galaxauraceae | *Galaxaura* | *filamentosa* | Chou | Maui, **Oahu** |
| Nemaliales | Galaxauraceae | *Galaxaura* | *rugosa* | (Ellis et Solander) Lamouroux | Hawaii, Kauai, Maui, Midway, Molokai, **Niihau**, Oahu |
| Nemaliales | Galaxauraceae | *Galaxaura* | sp. |  | Kauai |
| Nemaliales | Galaxauraceae | *Tricleocarpa* | *cylindrica* | (Ellis et Solander) Huisman et Borowitzka | Kauai, Lanai |
| Nemaliales | Galaxauraceae | *Tricleocarpa* | *fragilis* | (Linneaus) Huisman et Townsend | **French Frigate Shoals**, **Kauai**, Maui |
| Nemaliales | Galaxauraceae | *Tricleocarpa* | sp. |  | Kauai, Maui, Midway, **Molokai**, Oahu |
| Nemaliales | Liagoraceae | *Akalaphycus* | *setchelliae* | (Yamada) Huisman, Abbott et Sherwood | Kauai, Oahu |
| Nemaliales | Liagoraceae | *Dermonema* | *pulvinatum* | (Grunow ex Holmes) Fan | Kauai, Maui, Oahu |
| Nemaliales | Liagoraceae | *Dotyophycus* | *pacificum* | Abbott | Molokai, Oahu |
| Nemaliales | Liagoraceae | *Dotyophycus* | *yamadae* | (Ohmi et Itono) Abbott et Yoshizaki | Oahu |
| Nemaliales | Liagoraceae | *Ganonema* | *farinosum* | (Lamouroux) Fan et Wang | French Frigate Shoals, Kauai, Oahu |
| Nemaliales | Liagoraceae | *Ganonema* | *papenfussii* | (Abbott) Huisman, Abbott et Sherwood | **Hawaii**, Oahu |
| Nemaliales | Liagoraceae | *Ganonema* | *pinnatum* | (Harvey) Huisman | **French Frigate Shoals**, Oahu |
| Nemaliales | Liagoraceae | *Ganonema* | *samaense* | (C. K. Tseung) Huisman | Hawaii, Oahu |
| Nemaliales | Liagoraceae | *Ganonema* | sp. |  | Molokai |
| Nemaliales | Liagoraceae | *Ganonema* | *yoshizakii* | Huisman, Abbott et Sherwood | Hawaii |
| Nemaliales | Liagoraceae | *Helminthocladia* | *rhizoidea* | Doty et Abbott | **Maui**, Oahu |
| Nemaliales | Liagoraceae | *Helminthocladia* | *simplex* | Doty et Abbott | Oahu |
| Nemaliales | Liagoraceae | *Helminthocladia* | sp. |  | Maui |
| Nemaliales | Liagoraceae | *Izziella* | *orientalis* | (J. Agardh) Huisman et Schils | Oahu |
| Nemaliales | Liagoraceae | *Liagora* | *albicans* | Lamouroux | Hawaii, **Kauai**, Lanai, Oahu |
| Nemaliales | Liagoraceae | *Liagora* | *boergesenii* | Yamada | **Kauai**, Oahu |
| Nemaliales | Liagoraceae | *Liagora* | *ceranoides* | Lamouroux | Maui, Oahu |
| Nemaliales | Liagoraceae | *Liagora* | *divaricata* | C.K. Tseng | Oahu |
| Nemaliales | Liagoraceae | *Liagora* | *donaldiana* | Abbott et Huisman | Oahu |
| Nemaliales | Liagoraceae | *Liagora* | *hawaiiana* | Butters | **Hawaii**, Oahu |
| Nemaliales | Liagoraceae | *Liagora* | *julieae* | Abbott et Huisman | Oahu |
| Nemaliales | Liagoraceae | *Liagora* | *perennis* | Abbott | **Kauai**, **Lanai**, Oahu |
| Nemaliales | Liagoraceae | *Liagora* | *robusta* | Yamada | Hawaii, Oahu |
| Nemaliales | Liagoraceae | *Liagora* | sp. |  | Hawaii, Kauai, Maui, Molokai, Oahu |
| Nemaliales | Liagoraceae | *Liagora* | *valida* | Harvey | Maui, Oahu |
| Nemaliales | Liagoraceae | *Stenopeltis* | *gracilis* | (Yamada et Tanaka) Itono et Yoshizaki | **Midway**, Oahu |
| Nemaliales | Liagoraceae | *Stenopeltis* | *liagoroides* | (Yamada) Itono et Yoshizaki | **Kauai** |
| Nemaliales | Liagoraceae | *Titanophycus* | *validus* | (Harvey) Huisman, Saunders et Sherwood | Oahu |
| Nemaliales | Liagoraceae | *Trichogloea* | *lubrica* | J. Agardh | Oahu |
| Nemaliales | Liagoraceae | *Trichogloea* | *requienii* | (Montagne) Kützing | Maui, Oahu |
| Nemaliales | Liagoraceae | *Trichogloea* | sp. |  | Oahu |
| Nemaliales | Liagoraceae | *Trichogloeopsis* | *hawaiiana* | Abbott et Doty | Oahu |
| Nemaliales | Liagoraceae | *Trichogloeopsis* | *mucosissima* | (Yamada) Abbott et Doty | Hawaii |
| Nemaliales | Liagoraceae | *Yamadaella* | *caenomyce* | (Decaisne) Abbott | **Midway**, Oahu |
| Nemaliales | Scinaiaceae | *Scinaia* | *furcata* | Zablackis | **Hawaii**, **Kauai**, Oahu |
| Nemaliales | Scinaiaceae | *Scinaia* | *hormoides* | Setchell | Maui, Oahu |
| Nemastomatales | Nemastomataceae | *Predaea* | *laciniosa* | Kraft | **Hawaii**, Maui |
| Nemastomatales | Nemastomataceae | *Predaea* | sp. |  | Kauai, Oahu |
| Nemastomatales | Nemastomataceae | *Predaea* | *weldii* | Kraft et Abbott | **Hawaii**, **Kauai** |
| Nemastomatales | Schizymeniaceae | *Platoma* | *ardreanum* | Kraft et Abbott | Maui, Oahu |
| Nemastomatales | Schizymeniaceae | *Platoma* | sp. |  | **French Frigate Shoals**, **Hawaii**, Oahu |
| Nemastomatales | Schizymeniaceae | *Titanophora* | *pikeana* | (Dickie) J. Feldmann | Oahu |
| Peyssonneliales | Peysonneliaceae | *Peyssonnelia* | *conchicola* | Piccone et Grunow | Oahu |
| Peyssonneliales | Peysonneliaceae | *Peyssonnelia* | *inamoena* | Pilger | French Frigate Shoals, **Hawaii**, Maui, **Oahu** |
| Peyssonneliales | Peysonneliaceae | *Peyssonnelia* | *rubra* | (Greville) J. Agardh | Hawaii, **Nihoa**, Oahu |
| Peyssonneliales | Peysonneliaceae | *Peyssonnelia* | sp. |  | Hawaii, Maui, Oahu |
| Pihiellales | Pihiellaceae | *Pihiella* | *liagoraciphila* | Huisman, Sherwood et Abbott | Oahu |
| Plocamiales | Plocamiaceae | *Plocamium* | *sandvicense* | J. Agardh | Hawaii, Kauai, **Molokai**, Oahu |
| Rhodachlyales | Rhodachlyaceae | ***Rhodachlya*** | **sp.** |  | **Hawaii** |
| Rhodymeniales | Champiaceae | *Champia* | *parvula* | (C. Agardh) Harvey | **Hawaii**, Maui, Oahu |
| Rhodymeniales | Champiaceae | *Champia* | *vieillardii* | Kützing | **Hawaii**, HURL CRUISE, **Oahu** |
| Rhodymeniales | Champiaceae | *Champia* | sp. |  | Oahu, Maui |
| Rhodymeniales | Faucheaceae | *Gloiocladia* | *iyoensis* | (Okamura) R.E. Norris | HURL CRUISE, **Lanai**, **Maro** |
| Rhodymeniales | Faucheaceae | *Halichrysis* | *coalescens* | (Farlow) R.E. Norris et Millar | French Frigate Shoals, **Hawaii** |
| Rhodymeniales | Lomentariaceae | *Gelidiopsis* | *intricata* | (C. Agardh) Vickers | **Laysan**, Maui, Oahu |
| Rhodymeniales | Lomentariaceae | *Gelidiopsis* | *scoparia* | (Montagne ex Millardet) DeToni | Kauai, Oahu |
| Rhodymeniales | Lomentariaceae | *Gelidiopsis* | sp. |  | Hawaii, Kauai, Maui, Oahu |
| Rhodymeniales | Lomentariaceae | *Gelidiopsis* | *variabilis* | (J. Agardh) Schmitz | Hawaii, Oahu |
| Rhodymeniales | Lomentariaceae | *Lomentaria* | *hakodatensis* | Yendo | Hawaii, **Midway** |
| Rhodymeniales | Rhodymeniaceae | *Botryocladia* | *skottsbergii* | (Børgesen) Levring | Kauai, Maui, Oahu |
| Rhodymeniales | Rhodymeniaceae | *Botryocladia* | sp. |  | Oahu |
| Rhodymeniales | Rhodymeniaceae | *Botryocladia* | *tenuissima* | Taylor | **French Frigate Shoals** |
| Rhodymeniales | Rhodymeniaceae | *Chamaebotrys* | *boergesenii* | (Weber-van Bosse) Huisman | **Hawaii** |
| Rhodymeniales | Rhodymeniaceae | *Chrysymenia* | *glebosa* | Abbott et Littler | Maui |
| Rhodymeniales | Rhodymeniaceae | *Chrysymenia* | *kairnbachii* | Grunow | **Maro** |
| Rhodymeniales | Rhodymeniaceae | *Chrysymenia* | *okamurae* | Yamada et Sagawa | French Frigate Shoals, Hawaii, **Maro** |
| Rhodymeniales | Rhodymeniaceae | *Chrysymenia* | sp. |  | French Frigate Shoals, **Kauai** |
| Rhodymeniales | Rhodymeniaceae | *Coelarthrum* | *cliftonii* | (Harvey) Kylin | Oahu |
| Rhodymeniales | Rhodymeniaceae | *Coelothrix* | *irregularis* | (Harvey) Børgesen | Hawaii, Maui, **Molokai**, Oahu |
| Rhodymeniales | Rhodymeniaceae | *Erythrocolon* | *podagricum* | (J. Agardh. ex Grunow) J. Agardh ex Kylin | **Maro** |
| Rhodymeniales | Rhodymeniaceae | *Rhodymenia* | *leptophylla* | J. Agardh | Kauai |
| Rhodymeniales | Rhodymeniaceae | *Rhodymenia* | sp. |  | Kauai, **Necker** |
| Sporolithales | Sporolithaceae | *Sporolithon* | *ptychoides* | Heydrich | Oahu |
| Sporolithales | Sporolithaceae | *Sporolithon* | sp. |  | Oahu |
| Stylonematales | Stylonemataceae | *Chroodactylon* | *ornatum* | (C. Agardh) Basson | **Oahu** |
| Stylonematales | Stylonemataceae | ***Chroothece*** | **sp.** |  | **Oahu** |
| Stylonematales | Stylonemataceae | *Rhodosorus* | *marinus* | Geitler | Oahu |
| Stylonematales | Stylonemataceae | *Stylonema* | *alsidii* | (Zanardini) Drew | **Hawaii**, HURL CRUISE, **Kauai**, Maui, Oahu |
| Thoreales | Thoreaceae | *Chantransia* | sp.  (of *Nemalionopsis tortuosa*) |  | Maui |
