# Supplementary material for: The Hawaiian Rhodophyta Biodiversity Survey (2006-2010): a summary of principal findings
Source: BMC Plant Biol. 2010 Nov 22;10:258. doi: 10.1186/1471-2229-10-258 (PMC3012605; doi:10.1186/1471-2229-10-258)
Supplement: Additional file 3 — Sequence accessions. Hawaiian Algal Database accession numbers for all sequences analyzed as part of the Hawaiian Rhodophyta Biodiversity Survey. Accessions are given as superscripts for each of the three markers (N = nuclear LSU, M = mitochondrial COI, P = plastid UPA). [file 1471-2229-10-258-S3.DOCX]

**Additional file 3.** Sequence accessions.

| **Order** | **Family** | **Genus** | **Species** | **Authority** | **Accessions** |
| --- | --- | --- | --- | --- | --- |
| Acrosymphytales | Acrosymphytaceae | *Acrosymphyton* | *taylorii* | Abbott | 00261^N,M,P^ 00834^P^ |
| Bangiales | Bangiaceae | *Bangia* | *fuscopurpurea* | (Dillwyn) Lyngbye | 03783^M,P^ |
| Bangiales | Bangiaceae | *Porphyra* | *vietnamensis* | Tanaka et Pham | 00616^N,P^ 00866^M,P^ |
| Bangiales | Bangiaceae | *Porphyra* | sp. |  | 02788^M,P^ 02789^M,P^ 03516^P^ 03789^M,P^ 03791^M,P^ 04486^M,P^ 04487^M,P^ 04182^M,P^ 04183^M,P^ |
| Bangiales | Bangiaceae | *Pseudobangia* | sp. |  | 04181^M^ |
| Batrachospermales | Batrachospermaceae | *Chantransia* | sp. |  | 00079^M,P^ 00140^N,M,P^ 00168^P^ 02599^N,M,P^ |
| Batrachospermales | Batrachospermaceae | *Kumanoa* | *spermatiophora* | (M.L. Vis et Sheath) Entwisle, M.L. Vis, W.B. Chiasson, Necchi et A.R. Sherwood | 00508^M,P^ |
| Bonnemaisoniales | Bonnemaisoniaceae | *Asparagopsis* | *taxiformis* | (Delile) Trevisan | 00136^N,P^ 01132^N,M,P^ 01155^N,M,P^ 01156^N,M,P^ 01162^N,M^ 01163^M^ 01164^M^ 01165^N,M^ 01166^N,M^ 01201^M^ 01202^M^ 01206^M^ 01208^M^ 01209^M^ 01213^M^ 01214^M^ 01217^M^ 01218^M^ 01220^M^ 01221^M^ 01222^M^ 01224^M^ 01225^M^ 01226^M^ 01227^M^ 01693^M^ 01945^M^ 01946^M^ 01962^M^ 01965^M^ 01967^N,M,P^ 02247^M^ 02248^M^ 02249^M^ 02259^M^ 02260^N,M,P^ 02261^M^ 01726^N,M,P^ 01232^N,P^ |
| Bonnemaisoniales | Naccariaceae | *Reticulocaulis* | *mucosissimus* | Abbott | 00269^N^ 00935^M,P^ 00936^N,M,P^ |
| Ceramiales | Callithamniaceae | *Aglaothamnion* | *boergesenii* | (Aponte et Ballantine) L'Hardy-Halos et Rueness | 04082^N,P^ |
| Ceramiales | Callithamniaceae | *Aglaothamnion* | *cordatum* | (Børgesen) Feldmann- Mazoyer | 00621^N,M,P^ |
| Ceramiales | Callithamniaceae | *Aglaothamnion* | sp. |  | 02786^N^ 02848^M^ 02929^M^ 03488^N,M^ 03580^N,M,P^ 03659^M,P^ 04556^N^ |
| Ceramiales | Callithamniaceae | *Crouania* | *minutissima* | Yamada | 03338^M,P^ |
| Ceramiales | Callithamniaceae | *Crouania* | sp. |  | 02038^N,P^ 02541^M,P^ 02927^M^ 03194^P^ 03318^N,P^ |
| Ceramiales | Callithamniaceae | *Euptilocladia* | *magruderi* | Abbott et R. E. Norris | 00223^N,M,P^ 02314^M^ 02662^N,M,P^ 01778^N,M^ 02744^M,P^ 03616^M,P^ |
| Ceramiales | Callithamniaceae | *Gymnothamnion* | *elegans* | (Schousboe ex C. Agardh) J. Agardh | 00677^P^ 02517^N,M^ |
| Ceramiales | Callithamniaceae | unknown |  |  | 02721^N,M,P^ 03684^N,M,P^ 03700^N,M,P^ |
| Ceramiales | Ceramiaceae | *Acrothamnion* | *butleriae* | (Collins) Kylin | 02914^N,M^ 02915^N,M,P^ |
| Ceramiales | Ceramiaceae | *Antithamnion* | *antillanum* | Børgesen | 02189^N,P^ 02024^N,M,P^ 02542^N,P^ 03489^N,M^ |
| Ceramiales | Ceramiaceae | *Antithamnion* | *decipiens* | (J. Agardh) Athanasiadis | 01555^N,M,P^ |
| Ceramiales | Ceramiaceae | *Antithamnion* | *erucacladellum* | R. E. Norris | 02730^N,P^ |
| Ceramiales | Ceramiaceae | *Antithamnion* | sp. |  | 01557^N,P^ 01915^M^ 03779^N,M,P^ |
| Ceramiales | Ceramiaceae | *Antithamnionella* | sp. |  | 03237^N,M^ |
| Ceramiales | Ceramiaceae | *Balliella* | *repens* | Huisman et Kraft | 02262^P^ |
| Ceramiales | Ceramiaceae | *Centroceras* | *clavulatum* | (C. Agardh) Montagne | 02604^N,P^ 03255^N^ 03547^N^ 03573^N^ 03696^N,P^ |
| Ceramiales | Ceramiaceae | *Centroceras* | sp. |  | 03777^P^ 03786^M^ |
| Ceramiales | Ceramiaceae | *Ceramium* | *codii* | (Richards) G. Mazoyer | 03339^N,P^ |
| Ceramiales | Ceramiaceae | *Ceramium* | *dumosertum* | R. E. Norris et Abbott | 00847^N,M,P^ 03262^N,M,P^ 03316^N,M^ 03319^N,M,P^ 03257^N,M,P^ 03398^N,M,P^ 03401^N,M,P^ 03409^N,M,P^ 03428^N,P^ 03161^N,M,P^ 03574^N,M,P^ 03776^N,M,P^ 03683^N,M,P^ 03691^N,M,P^ 04081^N,P^ |
| Ceramiales | Ceramiaceae | *Ceramium* | *hanaense* | R. E. Norris et Abbott | 03261^N,M,P^ |
| Ceramiales | Ceramiaceae | *Ceramium* | *hyalacanthus* | (Kützing) Sonder | 02737^N^ |
| Ceramiales | Ceramiaceae | *Ceramium* | sp. |  | 01431^N,P^ 01465^N,M^ 00852^N,M^ 03032^N^ 03153^N^ 03260^N,M,P^ 03429^N,M,P^ 03197^N,P^ 03540^N^ 03593^N,M,P^ 03583^N,M,P^ 03571^N^ 03774^N,M,P^ 03687^N,P^ 03848^N,P^ 03198^M^ 03661^M^ 03841^M^ 01610^P^ |
| Ceramiales | Ceramiaceae | *Ceramium* | *womersleyi* | R. E. Norris et Abbott | 01943^N,M,P^ |
| Ceramiales | Ceramiaceae | *Corallophila* | *huysmansii* | (Weber-van Bosse) R. E. Norris | 03236^N,M,P^ |
| Ceramiales | Ceramiaceae | *Diplothamnion* | *jolyi* | van den Hoek | 01528^N,P^ 02889^N,P^ |
| Ceramiales | Ceramiaceae | *Gayliella* | sp. |  | 01432^N,M,P^ 03846^N,M,P^ 03693^N,M,P^ 03583^N,M,P^ 03689^N,M,P^ |
| Ceramiales | Ceramiaceae | *Perikladosporon* | *percurrens* | (Dawson) Athanasiadis | 02731^N,M,P^ 02751^M^ |
| Ceramiales | Dasyaceae | *Dasya* | *anastomosans* | (Weber-van Bosse) M.J. Wynne | 00648^N,P^ 01248^N^ |
| Ceramiales | Dasyaceae | *Dasya* | *baillouviana* | (S.G. Gmelin) Montagne | 04608^P^ |
| Ceramiales | Dasyaceae | *Dasya* | *corymbifera* | J. Agardh | 00641^N^ 00642^N^ 02859^N^ 03026^N,M,P^ 02887^N,M,P^ 04080^N,M,P^ |
| Ceramiales | Dasyaceae | *Dasya* | *iridescens* | (Schlech) Millar et Abbott | 00643^N,M,P^ 00644^N^ 00645^M^ 02605^M^ 02623^N,M,P^ 02920^N,M^ 02937^N,M,P^ 00360^N^ 02311^N,M,P^ 02319^N,M,P^ 02800^N,M^ 01607^N,M,P^ 01515^N,M,P^ 03423^N,P^ 02985^N,M^ 03640^N,P^ 03613^N,M^ 03101^M,P^ 03672^M^ 03752^M^ 04102^N,M,P^ 04104^N,M,P^ 04619^P^ 04620^N,P^ |
| Ceramiales | Dasyaceae | *Dasya* | *kristeniae* | Abbott | 02903^N,P^ |
| Ceramiales | Dasyaceae | *Dasya* | *murrayana* | Abbott et Millar | 03787^N^ 04527^N,P^ |
| Ceramiales | Dasyaceae | *Dasya* | sp. |  | 00650^N^ 00652^N,P^ 01243^N,M,P^ 02453^N,M,P^ 02723^N,M,P^ 02793^N^ 02917^N,M,P^ 03029^N,M,P^ 03117^N,M,P^ 03123^N,M,P^ 03136^N,M,P^ 03414^N,M,P^ 03641^N,P^ 03642^N,P^ 03628^M,P^ 03854^N,P^ 03668^M^ 04103^N,M,P^ 04101^N,M,P^ 04064^N,M,P^ 04610^N,P^ 04112^M^ 04609^P^ 04613^P^ |
| Ceramiales | Dasyaceae | *Heterosiphonia* | *crispella* | (C. Agardh) M.J. Wynne | 01426^N,P^ 02566^N,P^ 02483^N,M,P^ |
| Ceramiales | Dasyaceae | *Heterosiphonia* | sp. |  | 03333^N,P^ |
| Ceramiales | Delesseriaceae | *Hypoglossum* | *rhizophorum* | Ballantine et M.J. Wynne | 02023^M,P^ |
| Ceramiales | Delesseriaceae | *Martensia* | *flabelliformis* | Harvey ex J. Agardh | 00274^N,P^ 02933^N^ 02934^N^ 00749^M,P^ 00750^M^ |
| Ceramiales | Delesseriaceae | *Martensia* | *fragilis* | Harvey | 02607^N,P^ 00741^P^ 00135^P^ |
| Ceramiales | Delesseriaceae | *Martensia* | sp. |  | 01486^P^ 02935^N^ 02936^N^ 02938^N^ |
| Ceramiales | Delesseriaceae | *Nitophyllum* | *adhaerens* | Wynne | 01990^N^ 03342^M,P^ |
| Ceramiales | Delesseriaceae | *Taenioma* | *perpusillum* | (J. Agardh) J. Agardh | 02179^N,P^ 04528^N,P^ 03416^P^ |
| Ceramiales | Delesseriaceae | *Vanvoorstia* | *coccinea* | J. Agardh | 00789^P^ 00790^P^ |
| Ceramiales | Rhodomelaceae | *Acanthophora* | *pacifica* | (Setchell) Kraft | 00619^M^ 01779^N,M^ 00618^N,M,P^ 02010^P^ 03118^N,M,P^ 03125^N,M,P^ 03733^N,M,P^ 03720^N,M,P^ 03718^N,M,P^ |
| Ceramiales | Rhodomelaceae | *Acanthophora* | *spicifera* | (Vahl) Børgesen | 01487^N,P^ 01492^N,P^ 00126^N,M,P^ 02939^N^ 02940^N^ 03202^N^ 03203^N,P^ 03563^N,M,P^ 02951^M^ 02210^P^ |
| Ceramiales | Rhodomelaceae | *Alsidium* | *cymatophilum* | R.E. Norris | 03736^N,M,P^ 03704^N,M,P^ 02712^M^ 03597^M^ |
| Ceramiales | Rhodomelaceae | *Amansia* | *fimbrifolia* | (R. E. Norris) L.E. Phillips | 03086^N,M,P^ 03088^N,M,P^ 03089^N,P^ 03113^N,M,P^ 03114^N,M,P^ 03631^N,M,P^ 04075^N,P^ 04077^N,M,P^ 04055^M^ 03978^M^ 03985^P^ 03988^P^ |
| Ceramiales | Rhodomelaceae | *Amansia* | *glomerata* | C. Agardh | 00505^M,P^ 00744^M,P^ 00745^N,M,P^ 00746^N,M,P^ 00751^M,P^ 01498^N,M,P^ 01490^N,M,P^ 02202^N,M,P^ 02215^N,M,P^ 02743^N,M,P^ 00846^N,M,P^ 01594^M^ 01989^N,M,P^ 02806^N,M,P^ 02336^N,M,P^ 02923^N,M,P^ 02427^M^ 02809^P^ 02425^P^ 02426^P^ 02427^P^ 03087^N,M^ 03091^N,M,P^ 03126^N,M,P^ 03160^N,M,P^ 03241^N,M,P^ 03247^M,P^ 03539^M^ 03545^N,P^ 03623^N,M,P^ 03617^N,M,P^ 03596^N,P^ 03601^N,M,P^ 03879^P^ 03980^P^ 03981^P^ 04026^N^ 04027^N^ 04028^N^ 04029^N^ 04030^N^ 04031^N^ 04047^M,P^ 04049^P^ 04050^P^ 04052^P^ 04053^M,P^ 04056^M,P^ 04059^P^ 04061^P^ 04063^M,P^ 04074^N,M,P^ 04076^N,M,P^ 04078^N,M,P^ 04079^P^ 04083^N^ |
| Ceramiales | Rhodomelaceae | *Chondria* | *arcuata* | Hollenberg | 00628^M,P^ |
| Ceramiales | Rhodomelaceae | *Chondria* | *dangeardii* | Dawson | 00629^N,P^ 03592^N,M,P^ 03726^N,P^ |
| Ceramiales | Rhodomelaceae | *Chondria* | sp. |  | 02063^N^ 01780^N,M,P^ 01783^N,M,P^ 03562^N,M,P^ 03588^N,M,P^ 03857^M^ 04516^P^ 04529^N^ |
| Ceramiales | Rhodomelaceae | *Chondria* | sp.1 |  | 02916^N,M,P^ |
| Ceramiales | Rhodomelaceae | *Chondria* | sp.2 |  | 03847^N,M,P^ |
| Ceramiales | Rhodomelaceae | *Chondria* | sp.3 |  | 02748^N,P^ |
| Ceramiales | Rhodomelaceae | *Chondria* | sp.4 |  | 02062^N,M,P^ |
| Ceramiales | Rhodomelaceae | *Chondrophycus* | cf. *undulatus* | (Yamada) Garbary et Harper | 00733^N,M,P^ 00734^M^ 02386^N^ 02393^N^ 02493^N,M^ 02922^N,M,P^ 03322^N^ 03324^N,M,P^ |
| Ceramiales | Rhodomelaceae | *Chondrophycus* | *dotyi* | (Y. Saito) K.W. Nam | 00693^M,P^ 01606^N,M^ |
| Ceramiales | Rhodomelaceae | *Chondrophycus* | sp. |  | 00732^P^ 00734^P^ 00735^M,P^ 01994^M,P^ 02388^P^ 02389^P^ 02528^N,M,P^ 03326^N,M,P^ 04518^P^ 04519^P^ 04520^P^ |
| Ceramiales | Rhodomelaceae | *Chondrophycus* | *succisus* | (Cribb) K.W. Nam | 02387^N^ 02390^N,P^ 02583^N,M,P^ |
| Ceramiales | Rhodomelaceae | *Herposiphonia* | sp. |  | 01722^N,M,P^ 02733^N,M^ 02849^N,M^ 02901^N,P^ 03048^N,M,P^ 03083^N,M,P^ 03084^N,M,P^ 03171^N,M^ 03412^M^ 03612^N,P^ 03734^N,M,P^ 03845^N,M,P^ 03853^N,M,P^ 03785^N,M,P^ 04514^N,M,P^ |
| Ceramiales | Rhodomelaceae | *Janczewskia* | *hawaiiana* | Apt | 00683^N,M,P^ 02521^N,M,P^ 02523^N,M,P^ 03699^N,P^ 03702^N,M,P^ |
| Ceramiales | Rhodomelaceae | *Laurencia* | *brachyclados* | Pilger | 00684^N,M,P^ 00685^M,P^ 02539^N,M,P^ 02548^N^ 02550^N^ |
| Ceramiales | Rhodomelaceae | *Laurencia* | *crustiformans* | McDermid | 00688^M,P^ 02399^N,M^ 02403^N,M,P^ 02579^N,M,P^ 02715^N,M,P^ 02764^N^ 02713^N,M,P^ 03210^N,M,P^ 03321^N,M^ 03327^N,M,P^ |
| Ceramiales | Rhodomelaceae | *Laurencia* | *decumbens* | Kützing | 02701^N,P^ 02765^N^ 02768^N^ |
| Ceramiales | Rhodomelaceae | *Laurencia* | *galtsoffii* | Howe | 02580^N^ 03551^M,P^ |
| Ceramiales | Rhodomelaceae | *Laurencia* | *majuscula* | (Harvey) Lucas | 00686^M,P^ 00696^N,M,P^ 00698^P^ 02374^N,M,P^ 02379^N,M,P^ 02332^M,P^ 02316^N,M,P^ 02321^M,P^ 01603^N,M,P^ 01592^N,M,P^ 01601^M,P^ 02549^N,M,P^ 02551^N^ 02553^N^ 02692^N,M,P^ 02694^N^ 02695^N^ 02696^N^ 02697^N^ 02709^N^ 02700^N^ 02702^N^ 02703^N^ 02704^N^ |
| Ceramiales | Rhodomelaceae | *Laurencia* | *mcdermidiae* | Abbott | 00701^N,M,P^ 00702^N,M,P^ 01500^N,M,P^ 02003^M,P^ 02060^N^ 02378^N,P^ 02525^N^ 02522^N,M,P^ 02524^N,M,P^ 02531^N,M,P^ 03611^N,M,P^ 03698^N,M,P^ |
| Ceramiales | Rhodomelaceae | *Laurencia* | *nidifica* | J. Agardh | 00687^N,M,P^ 00704^M^ 00849^P^ 02526^N,M^ 02527^N^ 02485^N,M^ 02498^N,M,P^ 02581^N,M,P^ 03163^N^ |
| Ceramiales | Rhodomelaceae | *Laurencia* | *tenera* | Tseng | 04530^N^ |
| Ceramiales | Rhodomelaceae | *Laurencia* | sp. |  | 02317^P^ 00785^P^ 02552^N,M,P^ 02714^N,M,P^ 02799^M,P^ 02873^M^ 03328^N,M,P^ 03589^N,M,P^ 03629^N^ 03775^N,M,P^ 03796^M^ 04510^P^ 04513^N,M,P^ 04525^N,P^ |
| Ceramiales | Rhodomelaceae | *Laurencia* | sp.2 |  | 03729^N,M,P^ |
| Ceramiales | Rhodomelaceae | *Laurencia* | sp.3 |  | 03166^N,M,P^ |
| Ceramiales | Rhodomelaceae | *Laurencia* | sp.4 |  | 03515^N,M^ |
| Ceramiales | Rhodomelaceae | *Laurencia* | sp.5 |  | 03697^N,M,P^ |
| Ceramiales | Rhodomelaceae | *Laurencia* | sp.6 |  | 02760^N,M,P^ |
| Ceramiales | Rhodomelaceae | *Laurencia* | sp.7 |  | 02602^N,M,P^ |
| Ceramiales | Rhodomelaceae | *Leveillea* | *jungermannioides* | (Hering et Martens) Harvey | 01774^M^ 03850^N^ 04505^P^ 04508^N,P^ 04115^P^ 04526^N,P^ |
| Ceramiales | Rhodomelaceae | *Lophocladia* | *kipukaia* | Schlech | 00740^N,P^ |
| Ceramiales | Rhodomelaceae | *Lophocladia* | sp. |  | 00771^N,M,P^ 00772^N,M,P^ 00773^N,M,P^ 01590^N,M,P^ 00548^N,P^ 00774^N^ 00665^P^ |
| Ceramiales | Rhodomelaceae | *Osmundaria* | *obtusiloba* | (C. Agardh) R.E. Norris | 00658^N,M,P^ 03151^N,M,P^ 03949^N,M,P^ 03951^M,P^ 03800^P^ |
| Ceramiales | Rhodomelaceae | *Palisada* | *cartilaginea* | (Yamada) Garbary & Harper | 04534^N^ |
| Ceramiales | Rhodomelaceae | *Palisada* | cf. *cartilaginea* | (Yamada) Garbary & Harper | 02061^N,M,P^ 02435^N,M^ 02600^N^ 02601^N^ 02693^N,M,P^ 00689^P^ |
| Ceramiales | Rhodomelaceae | *Palisada* | *parvipapillata* | (C.K. Tseng) Nam | 02288^P^ 02364^N^ 02375^N,P^ 02529^N^ 02486^N^ 02557^N^ 02558^N^ 02587^N^ 02921^N,M,P^ 03238^N,P^ 03381^P^ |
| Ceramiales | Rhodomelaceae | *Palisada* | *yamadana* | (Howe) Nam | 02397^N^ 02398^N,M^ 02547^N^ 02554^N,P^ 03490^M^ |
| Ceramiales | Rhodomelaceae | *Palisada* | sp. |  | 00691^N,P^ |
| Ceramiales | Rhodomelaceae | *Polysiphonia* | *howei* | Hollenberg | 00844^N,M,P^ 03242^N,M^ 03246^N,M^ 03731^N,M,P^ 03724^N,M,P^ 04524^N,P^ |
| Ceramiales | Rhodomelaceae | *Polysiphonia* | sp. |  | 00851^N,M,P^ 01524^M,P^ 03172^N,P^ 03195^N,M,P^ 03199^N,P^ 03312^N,P^ 03235^N,P^ 03651^N,P^ 03419^N,M,P^ 03662^P^ 03735^N,M,P^ 03727^N,P^ 03725^N,P^ 03685^N,M,P^ 03688^N,M,P^ 03690^N,M,P^ 03692^N,M,P^ 03851^N,M,P^ 03852^N,M,P^ 04535^N^ |
| Ceramiales | Rhodomelaceae | *Polysiphonia* | *tepida* | Hollenberg | 03313^N^ |
| Ceramiales | Rhodomelaceae | *Polysiphonia* | *upolensis* | (Grunow) Hollenberg | 03263^N,M,P^ |
| Ceramiales | Rhodomelaceae | *Rhodolachne* | *decussata* | Wynne | 02570^N,P^ |
| Ceramiales | Rhodomelaceae | *Spirocladia* | *barodensis* | Børgesen | 02022^N,M,P^ |
| Ceramiales | Rhodomelaceae | *Spirocladia* | *hodgsoniae* | Abbott | 00754^P^ 00755^M,P^ |
| Ceramiales | Rhodomelaceae | *Tayloriella* | *dictyurus* | (J. Agardh) Kylin | 00757^N,P^ |
| Ceramiales | Rhodomelaceae | *Tolypiocladia* | *glomerulata* | (C. Agardh) Schmitz | 00786^P^ 00787^M,P^ 01134^M,P^ 01252^N,M,P^ 01723^M^ 02592^M,P^ 03630^N,M,P^ 03732^N,M,P^ |
| Ceramiales | Rhodomelaceae | *Ululania* | *stellata* | Apt et Schlech | 03565^N,P^ 03566^N,P^ 03567^N,P^ 03719^N,P^ 03717^N,P^ 03701^N,M,P^ |
| Ceramiales | Rhodomelaceae | unknown |  |  | 01530^N,M,P^ 01644^N,M,P^ 03524^N,M,P^ 03581^N,M,P^ 03572^N,M,P^ |
| Ceramiales | Sarcomeniaceae | *Malaconema* | *minimum* | Hollenberg | 01532^N^ |
| Ceramiales | Spyridiaceae | *Spyridia* | *filamentosa* | (Wulfen) Harvey | 01285^N,P^ 01287^N,P^ 01288^N,P^ 01292^N,P^ 01293^P^ 01294^N,P^ 01298^N,P^ 01299^P^ 01302^N,P^ 01307^N,P^ 01286^N^ 01295^N^ 01301^N^ 01303^N^ 01304^N^ 01306^N^ 01308^N^ 01309^N^ 01725^P^ 02519^N,P^ 01725^N^ 02593^N,M,P^ 02271^N^ 02279^N^ 02281^N^ 02774^N^ 02775^N^ 02776^N^ 02777^N^ 02812^P^ 02813^P^ 02858^N^ 02866^N^ 02868^N^ 02870^N^ 02871^N^ 02884^N,M,P^ 02897^N,P^ 02898^N,P^ 02899^N,P^ 02900^N,P^ 03030^N,M^ 03033^N,M^ 03140^N,M,P^ 03141^N,M,P^ 03109^N,P^ 03362^N,P^ 03363^N,P^ 03371^P^ 03391^P^ 03392^P^ 03400^P^ 03402^N,M,P^ 03408^N,P^ 03747^P^ 03764^N^ 02307^M,P^ 02814^M^ 02869^M^ 03258^M,P^ 03396^M,P^ 03399^M^ 03657^M,P^ 03748^M^ |
| Ceramiales | Wrangeliaceae | *Anotrichium* | sp. |  | 03584^N,P^ |
| Ceramiales | Wrangeliaceae | *Anotrichium* | *tenue* | (C. Agardh) Nägeli | 00623^P^ 02263^N,P^ 02515^N,P^ 03728^N,P^ |
| Ceramiales | Wrangeliaceae | *Griffithsia* | *heteromorpha* | Kützing | 00869^N^ 03548^N^ |
| Ceramiales | Wrangeliaceae | *Griffithsia* | *schousboei* | Montagne | 00870^N,P^ 00671^P^ 02632^P^ 03694^P^ |
| Ceramiales | Wrangeliaceae | *Griffithsia* | sp. |  | 01645^N,M,P^ 02904^N,P^ 03849^N,P^ |
| Ceramiales | Wrangeliaceae | *Griffithsia* | *subcylindrica* | Okamura | 00672^M,P^ 00673^M,P^ 00674^M,P^ 00675^N,M,P^ 02246^N,M,P^ |
| Ceramiales | Wrangeliaceae | *Haloplegma* | *duperreyi* | Montagne | 03794^M^ |
| Ceramiales | Wrangeliaceae | *Lejolisia* | *pacifica* | Itono | 00850^N^ |
| Ceramiales | Wrangeliaceae | *Monosporus* | *indicus* | Børgesen | 01427^N,M,P^ 00868^M^ |
| Ceramiales | Wrangeliaceae | *Pleonosporium* | sp. |  | 01539^N,P^ |
| Ceramiales | Wrangeliaceae | *Wrangelia* | *argus* | (Montagne) Montagne | 00791^P^ |
| Ceramiales | Wrangeliaceae | *Wrangelia* | *bicuspidata* | Børgesen | 00792^P^ |
| Ceramiales | Wrangeliaceae | *Wrangelia* | *elegantissima* | R. E. Norris | 00195^P^ 01185^P^ 02609^M^ 02628^N,M,P^ 03424^N^ 03425^N,M^ 04091^N^ 04110^N,M^ 04113^N^ |
| Ceramiales | Wrangeliaceae | *Wrangelia* | sp. |  | 04147^P^ 04091^P^ |
| Colaconematales | Colaconemataceae | *Colaconema* | sp.1 |  | 02734^N,P^ 03331^N,P^ 03334^N,M,P^ |
| Colaconematales | Colaconemataceae | *Colaconema* | sp.2 |  | 03537^N,M^ 03549^N,M,P^ 03550^N,P^ |
| Colaconematales | Colaconemataceae | *Colaconema* | sp.3 |  | 02791^N^ 03332^N,M,P^ 03336^N,P^ |
| Compsopogonales | Compsopogonaceae | *Compsopogon* | *coeruleus* | (Balbis ex C. Agardh) Montagne | 00216^N,P^ 02676^M,P^ 02687^P^ |
| Corallinales | Corallinaceae | *Amphiroa* | *foliacea* | Lamouroux | 00797^M,P^ 00799^N,M,P^ 02041^N,M,P^ |
| Corallinales | Corallinaceae | *Amphiroa* | sp. |  | 02782^N,P^ 01595^N^ 02308^P^ |
| Corallinales | Corallinaceae | *Amphiroa* | *valonioides* | Yendo | 00801^P^ 00802^N,M,P^ |
| Corallinales | Corallinaceae | *Arthrocardia* | sp*.* |  | 00803^P^ |
| Corallinales | Corallinaceae | cf*. Spongites* |  |  | 02829^N,M,P^ 02831^N,M,P^ 02834^N,M,P^ |
| Corallinales | Corallinaceae | *Corallina* | sp. |  | 02988^N,M^ |
| Corallinales | Corallinaceae | *Haliptilon* | *subulatum* | (Ellis et Solander) Johansen | 00806^M^ 00807^N,M^ 00804^N,M^ 02330^N,M,P^ 02667^N,M,P^ 03598^N,M,P^ 04507^N,M,P^ |
| Corallinales | Corallinaceae | *Hydrolithon* | *gardineri* | (Foslie) Verheij & Prud’homme van Reine | 02346^N,M,P^ 02348^N,M,P^ |
| Corallinales | Corallinaceae | *Hydrolithon* | *reinboldii* | (Weber-van Bosse & Foslie) Foslie | 02352^N,M,P^ 02357^N,M,P^ 02360^N,M,P^ 02815^N^ |
| Corallinales | Corallinaceae | *Hydrolithon* | sp. |  | 02817^N,M,P^ 02821^N^ 02816^N^ 03039^N^ 03266^N^ 02828^N,M,P^ |
| Corallinales | Corallinaceae | *Jania* | sp. |  | 02217^N,M,P^ 02668^N,M^ 02042^N^ 02925^N,M,P^ 04509^N,M,P^ 04511^N,M,P^ 04512^N,M,P^ 04517^P^ 04515^P^ |
| Corallinales | Corallinaceae | *Lithophyllum* | *insipidum* | Adey, Townsend & Boykins | 02347^N,M,P^ 02358^N,M,P^ 02573^N,P^ 02706^N^ 02818^N,M,P^ 03038^N^ |
| Corallinales | Corallinaceae | *Lithophyllum* | *kotschyanum* | Unger | 02355^N,M,P^ 02048^N^ 02825^N,P^ |
|  |  |  |  |  |  |
| Corallinales | Corallinaceae | *Metamastophora* | sp. |  | 02356^M^ |
| Corallinales | Corallinaceae | *Neogoniolithon* | *brassica-florida* | (Harvey) Setchell & L.R. Mason | 02353^N,P^ |
| Corallinales | Corallinaceae | *Pneophyllum* | *conicum* | (E.Y. Dawson) Keats, Y.M. Chamberlain & Baba | 02354^N,P^ 02359^N,M,P^ 02824^N,M,P^ 02827^N,P^ |
| Corallinales | Corallinaceae | *Pneophyllum* | sp. |  | 02823^N,M,P^ |
| Corallinales | Corallinaceae | *Titanoderma* | *prototypum* | (Foslie) Woelkerling, Y.M. Chamberlain & P.C. Silva | 02351^N,M^ 02832^N^ |
| Corallinales | Hapalidiaceae | *Mesophyllum* | *erubescens* | (Foslie) M. Lemoine | 02826^N,M,P^ 02835^N,M,P^ |
| Corallinales | Hapalidiaceae | *Phymatolithon* | sp. |  | 02350^N,P^ |
| Corallinales | Mastophoroideae | *Mastophora* | sp. |  | 02045^N,P^ 02716^N,P^ 02717^N,P^ 02718^N,P^ 02545^N,M,P^ 03037^N^ |
| Corallinales | unknown | Corallinales |  |  | 02342^N^ 02044^N,M,P^ 02047^M^ 02497^M^ 02565^N,M,P^ 02572^N,P^ 02784^N,P^ 02785^N,P^ 02783^N^ 03122^N,M,P^ 03265^N,P^ 03517^N^ 03521^N,M^ 03298^N,P^ 03564^N,M,P^ |
| Erythropeltidales | Incertae sedis | *Madagascaria* | *erythrocladioides* | J.A. West et N. Kikuchi | 04166^P^ 04167^P^ 04485^P^ |
| Erythropeltidales | Erythrotrichiaceae | *Erythrocladia* | sp. |  | 02875^N,M,P^ |
| Erythropeltidales | Erythrotrichiaceae | *Erythrotrichia* | sp. |  | 04170^P^ 04481^P^ 04482^P^ |
| Erythropeltidales | Erythrotrichiaceae | *Sahlingia* | *subintegra* | (Rosenvinge) Kornmann | 04168^P^ 04174^P^ 04175^P^ 04537^P^ 04538^P^ 04539^P^ 04540^P^ 04541^P^ 04542^P^ 04543^P^ 04544^P^ 04545^P^ 04546^P^ 04547^P^ 04548^P^ 04549^P^ 04550^P^ 04551^P^ 04552^P^ 04553^P^ 04554^P^ |
| Gelidiales | Gelidiaceae | *Gelidium* | *crinale* | (Turner) Gaillon | 02710^N,M^ 02711^N,M,P^ |
| Gelidiales | Gelidiaceae | *Gelidium* | *pluma* | Loomis | 00823^N^ 00824^N,M^ |
| Gelidiales | Gelidiaceae | *Gelidium* | *pusillum* | (Stackhouse) Le Jolis | 00825^N,P^ 00826^N,P^ 03245^N^ |
| Gelidiales | Gelidiaceae | *Gelidium* | *reediae* | Loomis | 00827^N,M,P^ 03337^N,M,P^ |
| Gelidiales | Gelidiaceae | *Gelidium* | sp. |  | 03645^N^ 03497^N^ 03520^N^ |
| Gelidiales | Gelidiaceae | *Pterocladiella* | *caerulescens* | (Kützing) Santelices et Hommersand | 00829^N^ 02621^N^ 03646^N^ |
| Gelidiales | Gelidiaceae | *Pterocladiella* | *capillacea* | (Gmelin) Santelices et Hommersand | 00832^N,M^ 00833^N^ 01494^N,P^ 00152^N,P^ 00503^N,P^ 02219^N^ 02669^N^ 03232^N,P^ 03518^N^ 03541^N^ 03543^N^ |
| Gelidiales | Gelidiaceae | *Pterocladiella* | sp. |  | 03496^N^ 03544^N^ |
| Gelidiales | Gelidiellaceae | *Gelidiella* | *acerosa* | (Forsskål) Feldmann et Hamel | 00816^N^ 02449^N^ 02612^N,M^ 02617^N,M^ |
| Gelidiales | Gelidiellaceae | *Gelidiella* | *machrisiana* | Dawson | 00819^N^ |
| Gelidiales | Gelidiellaceae | *Gelidiella* | sp. |  | 03325^N^ 02778^M,P^ |
| Gelidiales | unknown | Gelidiales |  |  | 00976^N^ |
| Gigartinales | Caulacanthaceae | *Caulacanthus* | *ustulatus* | (Turner ex Mertens) Kützing | 02569^N,M,P^ 02567^N,M,P^ |
| Gigartinales | Cystocloniaceae | *Hypnea* | *cervicornis* | J. Agardh | 00895^N,M^ 00547^P^ |
| Gigartinales | Cystocloniaceae | *Hypnea* | *chordacea* | Kützing | 02324^N,P^ 03162^N^ |
| Gigartinales | Cystocloniaceae | *Hypnea* | *cornuta* var. *stellulifera* | J. Agardh | 00899^M,P^ |
| Gigartinales | Cystocloniaceae | *Hypnea* | *musciformis* | (Wulfen in Jacquin) Lamouroux | 00900^N,M^ 00901^N,M,P^ 00902^N,P^ 00182^N,M,P^ 01992^N,M,P^ 02323^N,M,P^ 00552^P^ |
| Gigartinales | Cystocloniaceae | *Hypnea* | *nidifica* | J. Agardh | 00903^N,P^ |
| Gigartinales | Cystocloniaceae | *Hypnea* | *pannosa* | J. Agardh | 00905^N,P^ 03670^N,M,P^ |
| Gigartinales | Cystocloniaceae | *Hypnea* | sp. |  | 01489^N,P^ 01772^N,P^ 03110^N,M,P^ 03115^M^ 03542^N,M^ 03568^N,M^ 03587^N,M^ 03723^N,M,P^ |
| Gigartinales | Cystocloniaceae | *Hypnea* | *spinella* | (C. Agardh) Kützing | 00908^N,M,P^ |
| Gigartinales | Cystocloniaceae | *Hypnea* | *valentiae* | (Turner) Montagne | 00909^P^ 00910^N,M,P^ 00207^N,M,P^ |
| Gigartinales | Cystocloniaceae | *Hypneocolax* | *stellaris* ssp. *orientalis* | (Weber-van Bosse) Womersley | 00911^N,M,P^ 01537^N,M,P^ |
| Gigartinales | Dumontiaceae | *Dudresnaya* | *hawaiiensis* | R. K. S. Lee | 00886^N,M,P^ 00887^N,M,P^ 02452^N,M,P^ 03710^N,M,P^ |
| Gigartinales | Dumontiaceae | *Dudresnaya* | *littleri* | Abbott | 00359^N^ |
| Gigartinales | Dumontiaceae | *Gibsmithia* | *dotyi* | Kraft et Ricker | 00890^N,M,P^ 02801^N,P^ |
| Gigartinales | Dumontiaceae | *Gibsmithia* | *hawaiiensis* | Doty | 00893^N,P^ 00218^N,P^ 02624^N,P^ |
| Gigartinales | Gigartinaceae | *Chondracanthus* | *acicularis* | (Roth) Fredericq | 00880^N,P^ 02611^N,P^ |
| Gigartinales | Gigartinaceae | *Chondracanthus* | sp. |  | 03253^N^ |
| Gigartinales | Gigartinaceae | *Chondracanthus* | *tenellus* | (Harvey) Hommersand | 00881^N,P^ |
| Gigartinales | Gigartinaceae | *Chondrus* | *ocellatus* | Holmes | 00883^N,M,P^ 02209^N^ |
| Gigartinales | Gigartinaceae | *Mazzaella* | *volans* | (C. Agardh) J. Agardh | 00921^N,P^ |
| Gigartinales | Gloiosiphoniaceae | *Peleophycus* | *multiprocarpium* | Abbott | 00924^N,P^ |
| Gigartinales | Kallymeniaceae | *Kallymenia* | *sessilis* | Okamura | 00913^N,P^ 02874^N,P^ 01414^N^ |
| Gigartinales | Kallymeniaceae | *Kallymenia* | sp. |  | 02796^P^ 02803^N,P^ 01513^N,P^ |
| Gigartinales | Kallymeniaceae | *Kallymenia* | *thompsonii* | Abbott et McDermid | 00914^N^ |
| Gigartinales | Phyllophoraceae | *Ahnfeltiopsis* | *concinna* | (J. Agardh) Silva et DeCew | 00836^N,P^ 00837^N^ 00840^N,P^ 01510^N,P^ 00156^N,P^ 02203^N,M,P^ 02208^N,P^ 02610^N,P^ 03164^N,P^ 03555^N,P^ 03558^N,P^ 03600^N^ 00491^P^ |
| Gigartinales | Phyllophoraceae | *Ahnfeltiopsis* | *divaricata* | (Holmes) Masuda | 00838^N^ |
| Gigartinales | Phyllophoraceae | *Ahnfeltiopsis* | *flabelliformis* | (Harvey) Masuda | 00841^N,P^ 03211^N,M,P^ 03212^N,M,P^ 03556^N,P^ 03620^N^ 00876^P^ |
| Gigartinales | Phyllophoraceae | *Ahnfeltiopsis* | *pygmaea* | (J. Agardh) Silva et DeCew | 00842^N^ 03493^N,M^ |
| Gigartinales | Phyllophoraceae | *Ahnfeltiopsis* | sp. |  | 02212^N^ 03254^N^ 03495^N,M^ 03376^N^ |
| Gigartinales | Rhizophyllidaceae | *Portieria* | *hornemannii* | (Lyngbye) Silva | 00934^N,M,P^ 00188^N,M,P^ 02333^N,P^ 02340^N,P^ 03483^N,M^ 03250^M,P^ 03594^N,P^ 03625^P^ |
| Gigartinales | Solieriaceae | *Eucheuma* | *denticulatum* | (Burman) Collins et Hervey | 00888^N,P^ 00889^P^ 02865^N^ 03953^N,M,P^ |
| Gigartinales | Solieriaceae | *Kappaphycus* | *alvarezii* var. *tambalang* | Doty | 00917^N,P^ 02614^M,P^ |
| Gigartinales | Solieriaceae | *Kappaphycus* | *cottonii* | (Weber-van Bosse) Doty | 00916^N,P^ |
| Gigartinales | Solieriaceae | *Kappaphycus* | sp. |  | 02780^P^ 02861^N^ 03773^N,P^ 03954^N,M,P^ 03955^N,M,P^ 03956^N,M,P^ 03957^N,M,P^ |
| Gigartinales | Solieriaceae | *Kappaphycus* | *striatum* | (Schmitz) Doty ex Silva | 00919^N,P^ |
| Gigartinales | unknown | Gigartinales |  |  | 00884^N,P^ 00885^N,M,P^ 00906^N,M^ 00957^N,M,P^ 01782^N^ 01526^N,M,P^ 01717^N,M,P^ 02021^N,M,P^ 01602^N,M,P^ 02705^N,P^ 00292^N^ 03090^N,M^ 03669^N,P^ 04100^N^ 00929^P^ 01593^N,M,P^ |
| Gracilariales | Gracilariaceae | *Gracilaria* | *abbottiana* | Hoyle | 00939^N,M,P^ 00940^P^ |
| Gracilariales | Gracilariaceae | *Gracilaria* | *coronopifolia* | J. Agardh | 00184^N,M,P^ 01459^P^ 02322^N,M,P^ 01944^N,M^ 02893^N,M,P^ 03578^M,P^ |
| Gracilariales | Gracilariaceae | *Gracilaria* | *dawsonii* | Hoyle | 00941^N,P^ |
| Gracilariales | Gracilariaceae | *Gracilaria* | *dotyi* | Hoyle | 00204^M,P^ 00943^N,M,P^ 03491^N,M^ 03492^N,M^ |
| Gracilariales | Gracilariaceae | *Gracilaria* | *epihippisora* | Hoyle | 00945^N,P^ |
| Gracilariales | Gracilariaceae | *Gracilaria* | *parvispora* | Abbott | 00778^P^ 00949^N,P^ 00950^N,P^ 02756^N^ 02757^N^ 03514^N,M,P^ 03577^N,P^ 03606^P^ |
| Gracilariales | Gracilariaceae | *Gracilaria* | *salicornia* | (C. Agardh) Dawson | 00157^N,M,P^ 00662^N^ 02207^N,M,P^ 03023^N,M,P^ 03201^N,M^ 03200^N,M,P^ 03952^N,M^ 03579^M,P^ |
| Gracilariales | Gracilariaceae | *Gracilaria* | sp. |  | 01173^N,M,P^ 01995^M,P^ 02006^M^ 02012^M,P^ 02400^N,M,P^ 02401^N,M^ 02434^M,P^ 02326^N,M^ 02894^N,M^ 02895^N,M,P^ 00583^N,P^ 03028^M,P^ 03112^N,M,P^ 03323^N,M^ 03486^N,M^ 03501^M^ 03519^N,M^ 03546^N,M^ 03559^N,M,P^ 03561^M^ 03586^N,M^ |
| Gracilariales | Gracilariaceae | *Gracilaria* | *tikvahiae* | McLachlan | 00953^N,P^ |
| Gracilariales | Gracilariaceae | *Gracilariopsis* | *lemaneiformis* | (Bory) Dawson, Acleto et Foldvik | 00947^N,M,P^ 00948^M,P^ 00275^N,M,P^ |
| Halymeniales | Halymeniaceae | *Cryptonemia* | *yendoi* | Weber-van Bosse | 00960^N,P^ 02230^N,P^ |
| Halymeniales | Halymeniaceae | *Grateloupia* | cf*. filcina* |  | 02214^N,P^ 02443^N,M,P^ 02444^N,M,P^ 02447^N,P^ 02448^N,P^ 01999^N,P^ 02445^N,M,P^ 02446^N,P^ 01724^N^ 03966^N^ |
| Halymeniales | Halymeniaceae | *Grateloupia* | *filicina* | (Lamouroux) C. Agardh | 00961^P^ |
| Halymeniales | Halymeniaceae | *Grateloupia* | *hawaiiana* | Dawson | 00882^N,P^ 00963^P^ 00964^N,P^ 02004^N,M,P^ |
| Halymeniales | Halymeniaceae | *Grateloupia* | *phuquocensis* | Tanaka et Pham | 00966^N,P^ 02064^N^ 02606^N^ 02619^N^ 02431^N,P^ 01703^N^ 02442^N,M,P^ 02441^N,M,P^ 03365^N^ 04521^N,P^ |
| Halymeniales | Halymeniaceae | *Halymenia* | *formosa* | Harvey ex Kützing | 00970^N,M,P^ 00971^N,M,P^ 03838^N,M,P^ |
| Halymeniales | Halymeniaceae | *Halymenia* | *stipitata* | Abbott | 00972^P^ |
| Halymeniales | Halymeniaceae | *Polyopes* | *hakalauensis* | (Tilden) Abbott | 03251^N^ 00975^P^ 00166^P^ |
| Halymeniales | unknown | Halymeniales |  |  | 00956^P^ 00976^P^ |
| Hildenbrandiales | Hildenbrandiaceae | *Hildenbrandia* | *angolensis* | Welwitsch ex West et West | 00258^N,P^ 03844^N^ |
| Hildenbrandiales | Hildenbrandiaceae | *Hildenbrandia* | *rubra* | (Sommerfelt) Meneghini | 02594^N,M,P^ 02688^N,M^ 03139^N,P^ 03533^M^ |
| Nemaliales | Galaxauraceae | *Actinotrichia* | *fragilis* | (Forsskål) Børgesen | 00983^N,M^ 01478^N,M^ 02362^M,P^ |
| Nemaliales | Galaxauraceae | *Dichotomaria* | *marginata* | (Ellis et Solander) Lamarck | 00062^N,M,P^ 02532^N^ 02613^N,M,P^ 03560^N,M^ 01991^M^ 02331^M^ 03502^M^ 03379^M^ 03793^M^ 03795^M^ 03792^M^ |
| Nemaliales | Galaxauraceae | *Dichotomaria* | cf*. marginata* |  | 00998^M^ 02367^N^ 02368^N^ 02369^N^ 02371^N^ 02372^N^ 02377^N^ 02402^N^ 02291^N,M^ 03329^N,M^ 03330^N,M^ 03619^M^ |
| Nemaliales | Galaxauraceae | *Galaxaura* | *divaricata* | (Linnaeus) Huisman & Townsend | 00995^M^ |
| Nemaliales | Galaxauraceae | *Galaxaura* | *filamentosa* | Chou | 03116^N^ 03487^N,M^ |
| Nemaliales | Galaxauraceae | *Galaxaura* | *rugosa* | (Ellis et Solander) Lamouroux | 02213^N,M^ 03485^N,M^ 02608^M^ 02290^M^ 02206^M^ 03500^M^ 00984^P^ 01002^P^ 00064^P^ 00065^P^ 03839^P^ 03840^P^ |
| Nemaliales | Galaxauraceae | *Tricleocarpa* | *cylindrica* | (Ellis et Solander) Huisman et Borowitzka | 01096^N^ |
| Nemaliales | Galaxauraceae | *Tricleocarpa* | sp. |  | 02631^N,M^ 02309^N,M^ 02310^N,M^ 01135^N^ 03484^N,M^ 03499^M^ |
| Nemaliales | Liagoraceae | *Akalaphycus* | *setchelliae* | (Yamada) Huisman, Abbott et Sherwood | 00070^N,M,P^ 00049^N,P^ 00074^N,P^ 00986^P^ |
| Nemaliales | Liagoraceae | *Dermonema* | *pulvinatum* | (Grunow ex Holmes) Fan | 00987^P^ 00988^P^ 03552^N,M,P^ 03730^N^ |
| Nemaliales | Liagoraceae | *Dotyophycus* | *yamadae* | (Ohmi et Itono) Abbott et Yoshizaki | 00991^M,P^ |
| Nemaliales | Liagoraceae | *Ganonema* | *farinosum* | (Lamouroux) Fan et Wang | 00037^N,M,P^ 01005^M^ |
| Nemaliales | Liagoraceae | *Ganonema* | *papenfussii* | (Abbott) Huisman, Abbott et Sherwood | 00033^N,M,P^ |
| Nemaliales | Liagoraceae | *Ganonema* | *pinnatum* | (Harvey) Huisman | 01009^N,M^ 00041^N,M^ 00041^P^ 02807^P^ |
| Nemaliales | Liagoraceae | *Ganonema* | *samaense* | (C. K. Tseung) Huisman | 00068^M,P^ |
| Nemaliales | Liagoraceae | *Ganonema* | *yoshizakii* | Huisman, Abbott et Sherwood | 00054^N,M,P^ 01012^P^ |
| Nemaliales | Liagoraceae | *Helminthocladia* | *rhizoidea* | Doty et Abbott | 03686^N^ 03858^M^ |
| Nemaliales | Liagoraceae | *Izziella* | *orientalis* | (J. Agardh) Huisman et Schils | 01017^N,M,P^ 01038^N,P^ 02577^N,M,P^ 02578^N,M,P^ 03529^N,P^ |
| Nemaliales | Liagoraceae | *Liagora* | *albicans* | Lamouroux | 01019^N^ 00056^N,M,P^ 00057^N,M^ 03695^N,M,P^ 01018^P^ 00057^P^ 03873^P^ |
| Nemaliales | Liagoraceae | *Liagora* | *boergesenii* | Yamada | 01021^M^ |
| Nemaliales | Liagoraceae | *Liagora* | *ceranoides* | Lamouroux | 02629^N^ 00051^P^ |
| Nemaliales | Liagoraceae | *Liagora* | *divaricata* | Tseng | 00053^N,P^ 02620^M,P^ |
| Nemaliales | Liagoraceae | *Liagora* | *donaldiana* | Abbott et Huisman | 00044^N,M,P^ |
| Nemaliales | Liagoraceae | *Liagora* | *julieae* | Abbott et Huisman | 00050^N,M,P^ |
| Nemaliales | Liagoraceae | *Liagora* | *perennis* | Abbott | 00042^N,P^ 00052^N,P^ 00206^N,P^ 00205^N,P^ 01031^P^ |
| Nemaliales | Liagoraceae | *Liagora* | *robusta* | Yamada | 01035^N,P^ |
| Nemaliales | Liagoraceae | *Liagora* | sp. |  | 02205^N,P^ 02329^N,P^ 01502^N^ 02918^N,P^ 02919^N,P^ 02924^N,P^ 03335^N,M,P^ 03498^N^ 03383^N^ 03536^N,M^ 03538^N,M^ 02001^M^ 02629^P^ |
| Nemaliales | Liagoraceae | *Stenopeltis* | *gracilis* | (Yamada et Tanaka) Itono et Yoshizaki | 00071^N,P^ 00072^N,M,P^ 03722^N,M,P^ 03721^N,P^ |
| Nemaliales | Liagoraceae | *Titanophycus* | *validus* | (Harvey) Huisman, G.W. Saunders et A.R. Sherwood | 00035^M,P^ |
| Nemaliales | Liagoraceae | *Trichogloea* | *lubrica* | J. Agardh | 01044^N,M,P^ 00055^N,M,P^ 03706^N,P^ |
| Nemaliales | Liagoraceae | *Trichogloea* | *requienii* | (Montagne) Kützing | 03554^N^ 03679^N,P^ |
| Nemaliales | Liagoraceae | *Trichogloea* | sp. |  | 03426^N^ |
| Nemaliales | Liagoraceae | *Yamadaella* | *caenomyce* | (Decaisne) Abbott | 00047^N,P^ 03967^N^ 02576^M^ |
| Nemaliales | Scinaiaceae | *Scinaia* | *furcata* | Zablackis | 00264^N^ 03681^N,M,P^ 03682^N,M,P^ |
| Nemaliales | Scinaiaceae | *Scinaia* | *hormoides* | Setchell | 03680^N,M,P^ |
| Nemastomatales | Nemastomataceae | *Predaea* | *laciniosa* | Kraft | 01103^N,P^ |
| Nemastomatales | Nemastomataceae | *Predaea* | sp. |  | 02664^N,M,P^ 01137^N,M,P^ 01413^N^ 00666^N^ 04092^N^ |
| Nemastomatales | Nemastomataceae | *Predaea* | *weldii* | Kraft et Abbott | 01105^N^ |
| Nemastomatales | Schizymeniaceae | *Platoma* | *ardreanum* | Kraft et Abbott | 00931^N,M,P^ 00932^N,M,P^ |
| Nemastomatales | Schizymeniaceae | *Platoma* | sp. |  | 02665^N^ 02863^N,M,P^ 03099^N,M,P^ |
| Nemastomatales | Schizymeniaceae | *Titanophora* | *pikeana* | (Dickie) J. Feldmann | 00937^M,P^ |
| Nemastomatales | unknown | Nemastomatales |  |  | 00273^N^ 00267^N^ 03130^N,M,P^ 03219^N,M,P^ 03100^N^ 03745^N^ |
| Peyssonneliales | Peysonneliaceae | *Peyssonnelia* | *conchicola* | Piccone et Grunow | 00926^N,P^ |
| Peyssonneliales | Peysonneliaceae | *Peyssonnelia* | *inamoena* | Pilger | 00927^P^ 00928^N^ 00162^N,P^ 00137^N,P^ |
| Peyssonneliales | Peysonneliaceae | *Peyssonnelia* | *rubra* | (Greville) J. Agardh | 00929^N^ 00930^N,M,P^ 00180^N,P^ 02595^N,P^ |
| Peyssonneliales | Peysonneliaceae | *Peyssonnelia* | sp. |  | 01238^N,P^ 01240^N,P^ 01472^N,P^ 01987^N,P^ 03036^N^ 03066^N^ 03078^N^ 03079^N^ |
| Pihiellales | Pihiellaceae | *Pihiella* | *liagoraciphila* | Huisman, Sherwood et Abbott | 04484^P^ |
| Plocamiales | Plocamiaceae | *Plocamium* | *sandvicense* | J. Agardh | 00240^N,M,P^ 00933^M,P^ 02337^N^ 02932^N^ |
| Rhodachlyales | Rhodachlyaceae | *Rhodachlya* | sp. |  | 03778^N,P^ |
| Rhodymeniales | Champiaceae | *Champia* | *parvula* | (C. Agardh) Harvey | 01115^M,P^ 02758^N^ 02759^N^ 02707^N^ 03027^N^ 03124^N,M^ 03158^M^ 03397^M^ |
| Rhodymeniales | Champiaceae | *Champia* | *vieillardii* | Kützing | 01527^N,M,P^ 01776^N,M,P^ 03737^N,M,P^ |
| Rhodymeniales | Champiaceae | *Champia* | sp. |  | 03170^N,P^ |
| Rhodymeniales | Faucheaceae | *Gloiocladia* | *iyoensis* | (Okamura) R. E. Norris | 03705^N,M,P^ |
| Rhodymeniales | Faucheaceae | *Halichrysis* | *coalescens* | (Farlow) R. E. Norris et Millar | 01169^N,P^ |
| Rhodymeniales | Lomentariaceae | *Gelidiopsis* | *scoparia* | (Montagne ex Millardet) DeToni | 01129^N,M,P^ 01705^N,M,P^ |
| Rhodymeniales | Lomentariaceae | *Gelidiopsis* | sp. |  | 03494^N,M^ 03797^M^ |
| Rhodymeniales | Lomentariaceae | *Lomentaria* | *hakodatensis* | Yendo | 03403^N,M^ |
| Rhodymeniales | Rhodymeniaceae | *Botryocladia* | *skottsbergii* | (Børgesen) Levring | 02363^N,M,P^ 00245^N,P^ 02603^N,M,P^ |
| Rhodymeniales | Rhodymeniaceae | *Chrysymenia* | *kairnbachii* | Grunow | 01118^N^ 01119^N^ |
| Rhodymeniales | Rhodymeniaceae | *Chrysymenia* | *okamurae* | Yamada et Sagawa | 01120^P^ |
| Rhodymeniales | Rhodymeniaceae | *Chrysymenia* | sp. |  | 02430^N,P^ |
| Rhodymeniales | Rhodymeniaceae | *Coelarthrum* | *cliftonii* | (Harvey) Kylin | 01231^N^ |
| Rhodymeniales | Rhodymeniaceae | *Coelothrix* | *irregularis* | (Harvey) Børgesen | 01122^N,M,P^ 01123^N,M,P^ 01128^N,M,P^ 02530^N,M,P^ 03111^N,P^ 01996^M^ |
| Rhodymeniales | Rhodymeniaceae | *Erythrocolon* | *podagricum* | (J. Agardh. ex Grunow) J. Agardh ex Kylin | 01124^P^ |
| Rhodymeniales | Rhodymeniaceae | *Rhodymenia* | sp. |  | 02432^N,M,P^ |
| Rhodymeniales | unknown | Rhodymeniales |  |  | 01112^N^ 01171^N,P^ 01116^N,P^ 01719^N,M,P^ 01531^N,M,P^ 01536^N,M^ 02540^N,M,P^ 04089^N,M,P^ 04090^N,M,P^ 01988^M^ 04146^M^ |
| Sporolithales | Sporolithaceae | *Sporolithon* | *ptychoides* | Heydrich | 02349^N,P^ 02819^N,M,P^ |
| Sporolithales | Sporolithaceae | *Sporolithon* | sp. |  | 02833^N^ |
| Stylonematales | Stylonemataceae | *Chroodactylon* | *ornatum* | (C. Agardh) Basson | 04177^P^ 04179^P^ 04178^P^ 04461^P^ 04462^P^ |
| Stylonematales | Stylonemataceae | *Stylonema* | *alsidii* | (Zanardini) Drew | 04163^P^ 04176^P^ 04464^P^ 04164^P^ 04465^P^ 04466^P^ 04468^P^ 04469^P^ 04470^P^ 04475^P^ 04476^P^ 04477^P^ 04479^P^ 04478^P^ 04480^P^ 04483^P^ |
| Stylonematales | Stylonemataceae | *Stylonema-like* |  |  | 04184^P^ 04165^P^ |
| Stylonematales | unknown Stylonematales |  |  |  | 04139^P^ |
| Thoreales | Thoreaceae | *Chantransia* phase of *Nemalionopsis* | sp*.* |  | 02685^P^ |
